# Small viruses reveal bidirectional evolution between HK97-fold viruses and encapsulins via procapsids

**DOI:** 10.1038/s41467-026-78136-1

**Published:** 2026-10-14

**Authors:** Abelardo Aguilar, Ingrid Miranda Perez, Omer Nadel, Stephen Nayfach, Luis Ruiz Pestana, Anca M. Segall, Forest L. Rohwer, Simon Roux, Simon White, Antoni Luque

**Affiliations:** 1 Department of Biology, University of Miami, Coral Gables, FL USA; 2 Department of Evolutionary Biology, Universidad Nacional Autónoma de México, Ciudad Universitaria, Mexico City, México; 3 Profluent Bio, Emeryville, CA USA; 4 Department of Civil and Architectural Engineering, University of Miami, Coral Gables, FL USA; 5 Department of Chemical, Environmental, and Materials Engineering, University of Miami, Coral Gables, FL USA; 6 Department of Biology, San Diego State University, San Diego, CA USA; 7 Viral Information Institute, San Diego State University, San Diego, CA USA; 8 Department of Energy Joint Genome Institute, Lawrence Berkeley National Laboratory, Berkeley, CA USA; 9 Department of Molecular and Cell Biology, University of Connecticut, Storrs, CT USA; 10 Department of Physics, University of Miami, Coral Gables, FL USA

**Keywords:** Molecular evolution, Protein sequence analyses, Phage biology

## Abstract

The HK97-fold is an ancient, conserved protein structure that assembles protein shells, forming the capsids of *Duplodnaviria* viruses and prokaryotic biochemical compartments called encapsulsins. It has been proposed that encapsulins evolved from HK97-fold viruses. But the higher complexity of viral capsids represents a biophysical paradox in the evolution of HK97-fold protein shells. To resolve this problem, we hypothesize the existence of simpler HK97-fold viral capsids associated with smaller genomes than those of known HK97-fold viruses. Such small viral genomes are searched in environmental metagenomes across public databases, including IMG/M and MGnify. Using hidden Markov models and protein language models, we identify and characterize small viral genomes encoding HK97-fold proteins that exhibit molecular similarities to encapsulins yet lack portal and tail genes. The phylogenetic analyses of HK97-fold proteins reveal independent mixed groups of viruses and encapsulins, and structural analyses identify similarities between encapsulins and procapsids, the immature state of viral capsids. The observations support a bidirectional evolution between encapsulins and HK97-fold viruses enabled by the viral procapsid state. Using biophysical and genomic arguments, we introduce an evolutionary model of HK97-fold protein shells, predicting that they emerged as lower-symmetry biochemical compartments and that HK97-fold viral capsids may retain biochemical functions.

## Abstract (web summary)

In this study, mining environmental metagenomics data, the authors show that small HK97-fold-encoding viral genomes bridge Duplodnaviria viral capsids and cellular protein shells, revealing bidirectional evolution in which viral procapsids enable the transitions between viral and metabolic compartments.

## Introduction

**Fig. 1:**
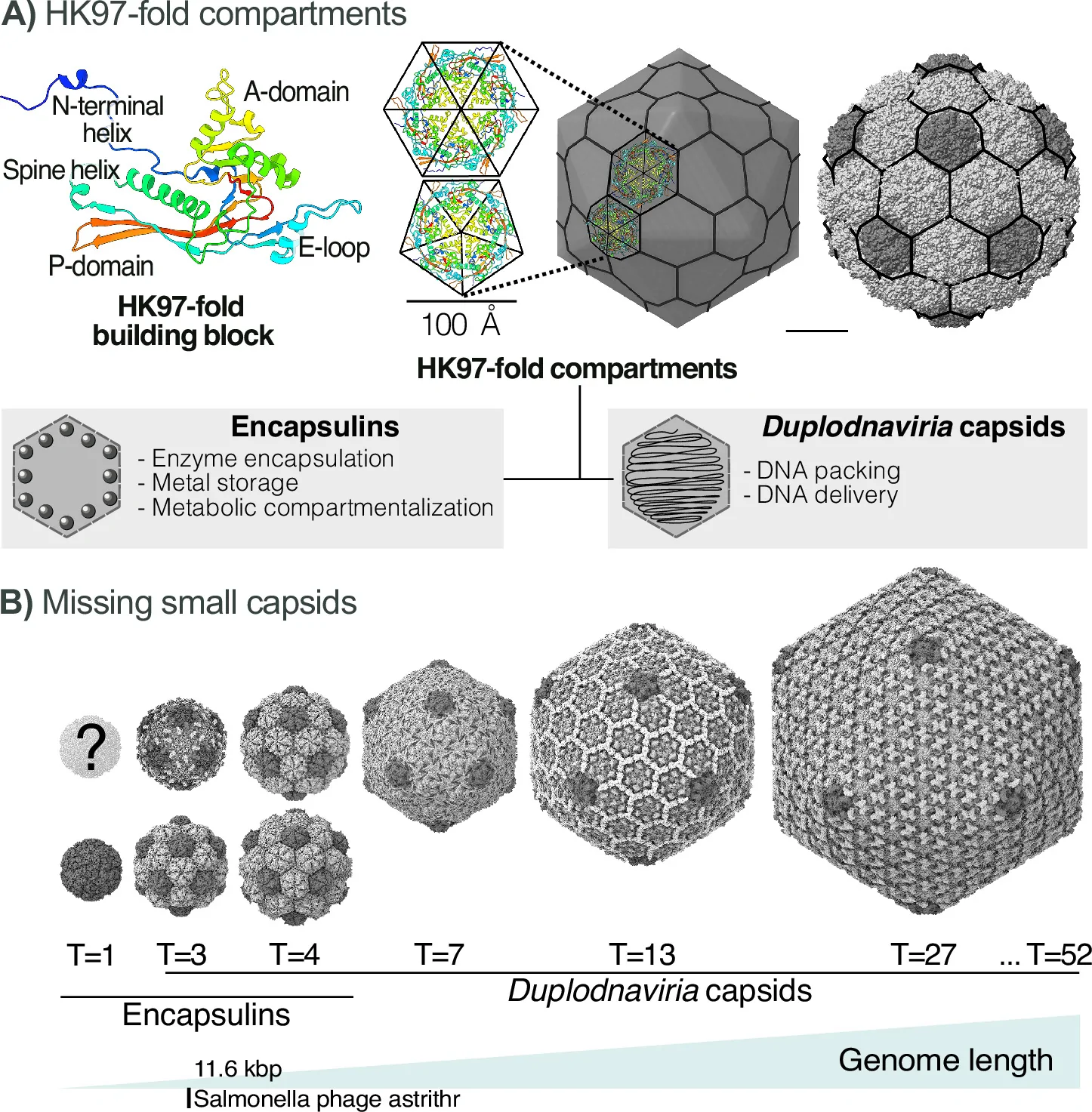
Size distribution of HK97-fold biochemical and genomic compartments. **A** Schematic representation of the HK97-fold and its canonical domains displayed with different colors. The geometric and rendered capsids illustrate the distribution of capsomers as pentamers (five proteins) and hexamers (six proteins). The gray boxes describe the two main compartments made of HK97-fold proteins. **B** Size range of HK97-fold compartments showcasing geometric capsid architectures (T-numbers). The T-number (or triangulation number) is the number of major capsid proteins in the asymmetric unit, defined by the equation T = h^2^ + h·k + k^2^, where h and k are two non-negative integers counting the steps needed to join two nearby pentamers along the underlying hexagonal lattice. The first elements of the canonical sequence of T are 1, 3, 4, 7, and 60·T is the number of major capsid proteins in the icosahedral shell^47,48^. Reconstructed *Duplodnaviria* capsids and encapsulins are displayed in order by T-number. The blue gradient indicates the increase in genome length or capsid packaging capacity. The vertical line marks the smallest known *Duplodnaviria* genome as a reference.

One longstanding debate on the origin of life addresses whether life began primarily through the emergence of self-replicating informational molecules endowed with catalytic activity (a genetics-first model), like the RNA world hypothesis^1,2^, or as a cooperative network of autocatalytic reactions (a metabolism-first model) that preceded genetic polymers^3–6^. Since modern cells require both metabolism and genetics, recent formulations on the origin of life explore hybrid scenarios in which primordial replicators and metabolic systems co-evolved and became functionally integrated^7–9^. This view acknowledges that informational and metabolic processes likely became linked early on, rather than one strictly preceding the other. A critical step in any origin-of-life scenario is the emergence of compartments that could concentrate reactants, protect fragile molecules, and favor the exchange of molecules^10–12^. Indeed, life at its core is about creating distinct chemical spaces through compartmentalization. Contemporary biological compartments can be interpreted as imprints or milestones in the evolution of life and are invaluable for extracting clues on the origin of life^13^. HK97-fold proteins represent an intriguing group found in all domains of life^14,15^, and form icosahedral shells capable of performing both genomic and metabolic functions^16–19^ (Fig. 1A). Although HK97-fold proteins are among the most prevalent on Earth^20,21^ and serve as key links between biochemical and genetic compartmentalization, they have been overlooked in the debate about the origin of life.

HK97-fold proteins are a highly diverse group of proteins in sequence space^22^, and they are characterized by displaying the conserved HK97-fold or Johnson fold^23,24^ (Fig. 1A). The subset of HK97-fold proteins with genomic functions forms the capsids protecting the genome of viruses in the *Duplodnaviria* realm^15,25^. This includes tailed prokaryotic viruses that infect Bacteria and Archaea^17,19,26,27^, related prokaryotic mobile elements, like phage inducible chromosomal islands (PICIs) and gene transfer agents (GTAs)^28–30^, and non-tailed eukaryotic viruses infecting plankton (mirusviruses)^31^ and animals (herpesviruses)^32,33^. The other subset of HK97-fold proteins forms encapsulins in Bacteria and Archaea, cellular compartments that carry metabolic functions including iron storage^34,35^, sulfur metabolism^36^, and oxidative stress response^18,37^. These functions have been proposed as ancient^38–41^.

Initial sequence and structural analyses of HK97-fold proteins were either inconclusive about their origin or favored viral HK97-fold proteins as ancestral, given their presence across more lineages^15^. A more recent and exhaustive phylogenetic and structural evolutionary study of HK97-fold proteins, using an expanded set of encapsulins, indicated that encapsulins are likely derived from HK97-fold viruses^42^. However, the evolutionary model of encapsulins emerging from viruses does not provide a rationale for how HK97-fold shells evolved metabolic functions from shells that carried genomic functions, or for how known HK97-fold viruses, which form far more complex structures than encapsulins^43^, would have emerged before encapsulins. Prior evolutionary models noted but did not explicitly incorporate the differences in structural complexity between viral capsids and encapsulins, which typically form small *T* = 1 or *T* = 3 shells^15,42^.

The HK97-fold proteins in encapsulins co-assemble around several copies of a protein called cargo, encoded in the same operon and responsible for catalyzing the biochemical reactions^37,44^. Most encapsulins form protein shells built from 60 HK97-fold proteins distributed in 12 pentamers (oligomers of five proteins) as the vertices of the simplest icosahedral architecture, *T* = 1, several form *T* = 3, and a few *T* = 4 icosahedral architectures combining pentamers at the vertices and hexamers on the faces^42,44,45^ (Fig. 1B). For some encapsulins, the presence of cargo proteins promotes the assembly of a larger icosahedral T-number shell than without them^46^. T is the triangulation number or T-number, which indicates the number of major capsid proteins (MCPs) in the asymmetric unit and indexes icosahedral capsid size and geometry, with larger T corresponding to larger HK97-fold shells^47,48^ (Fig. 1B).

Unlike encapsulin assembly, which often involves only the major capsid protein-like and the cargo protein, the maturation of HK97-fold viruses is more complex. In addition to HK97-fold proteins, it involves scaffold proteins (sometimes fused with the HK97-fold), portal proteins to facilitate DNA translocation, a terminal complex to ensure packaging, and often minor proteins that reinforce the structure^29,49–53^.

The HK97-fold proteins, guided by the scaffold proteins, assemble around a portal complex that is responsible for the translocation of the double-stranded (ds) DNA genome within the capsid^54–57^. This forms an empty shell called a procapsid, which, as the double-stranded (ds) DNA is packaged through the portal, expands, releases the scaffold proteins, and becomes more faceted^24,58–62^. Bacterial and archaeal viruses, along with prokaryotic mobile elements that build capsids with HK97-fold proteins, append to the portal a protein tail apparatus that enables the delivery of dsDNA into the host^63–65^. HK97-fold viral genomes display a wide range of lengths due to the versatility of HK97-fold proteins forming capsid architectures of different sizes^17,26,43,66^. Most *Duplodnaviria* viruses form primarily *T* = 7 structures followed by *T* = 13 and *T* = 16 structures^17,32,43,67–69^ (Fig. 1B). The largest HK97-fold viral capsid reconstructed corresponds to Lysinbacillus Phage G, forming a *T* = 52 architecture, containing more than 3,000 major capsid proteins and packaging a dsDNA genome that is 626 kbp (kilo base pairs) long^26,70^. The smallest reconstructions correspond to isometric *T* = 3 capsid variants of the Bacillus phage ϕ29^71^, which packages its genome in elongated *T* = 3, *Q* = 5 capsids^72^. Quasi-spherical *T* = 4 capsid reconstructions have been obtained for Streptococci C1 phage^73^, the satellite Escherichia phage P4^74^, and the *Staphylococcus aureus* pathogenic island 1 or SaPI1^29^. The smallest tailed phage genomes are 11.6 kbp long from the non-satellite Salmonella phage astrithr^75^ and the satellite phage P4^76^. The smallest HK97-fold capsid characterized in association with genetic material packaging functions corresponds to the *Rhodobacter capsulatus* gene transfer agent (RcGTA), which is encoded in a 15 kbp genomic region but instead packages 4 to 4.5 dsDNA fragments derived from the host genome. This RcGTA assembles a shortened, oblate *T *= 3-like head^28^. To our knowledge, there is no documented evidence of *Duplodnaviria* viruses or HK97-fold gene transfer agents forming *T* = 1 capsids (Fig. 1B)^43^.

The greater structural complexity of HK97-fold viral capsids compared with encapsulins makes it tempting, under a parsimony argument, to consider the possibility of viruses evolving from encapsulins^43^, in apparent tension with the “viruses-to-encapsulins” scenario^42^. We hypothesize that if encapsulins emerged from viruses, smaller HK97-fold viruses may still be undiscovered. Regardless, finding such viruses or confirming their absence would help illuminate ancestral HK97-fold compartments. We built our approach on three key elements. First, genomes associated with HK97-fold viruses are prevalent when sequencing environmental samples and display a much higher diversity than the genomes from isolated *Duplodnaviria* viruses^20,21,77–79^. Second, the genome length of HK97-fold viruses follows a well-established allometric relationship with their capsid size and architecture^22,43,80^. Third, HK97-fold viruses can encode direct terminal repeats (DTRs) in their genomes, providing a bioinformatic proxy for identifying complete genomes among uncultured viruses^81–83^. A previous study combined these elements to search for simple HK97-fold viral genomes in human gut metagenomes^43^. It found 14 candidates encoding HK97-fold proteins in genomes below the isolated threshold of 11.6 kbp, which we defined here as the twilight zone of HK97-fold viral genomes. The genomes ranged from 6.9 kbp to 10.0 kbp, with no candidates in the range of 1 kbp to 5 kbp predicted to be compatible with *T* = 1 architectures.

**Fig. 2:**
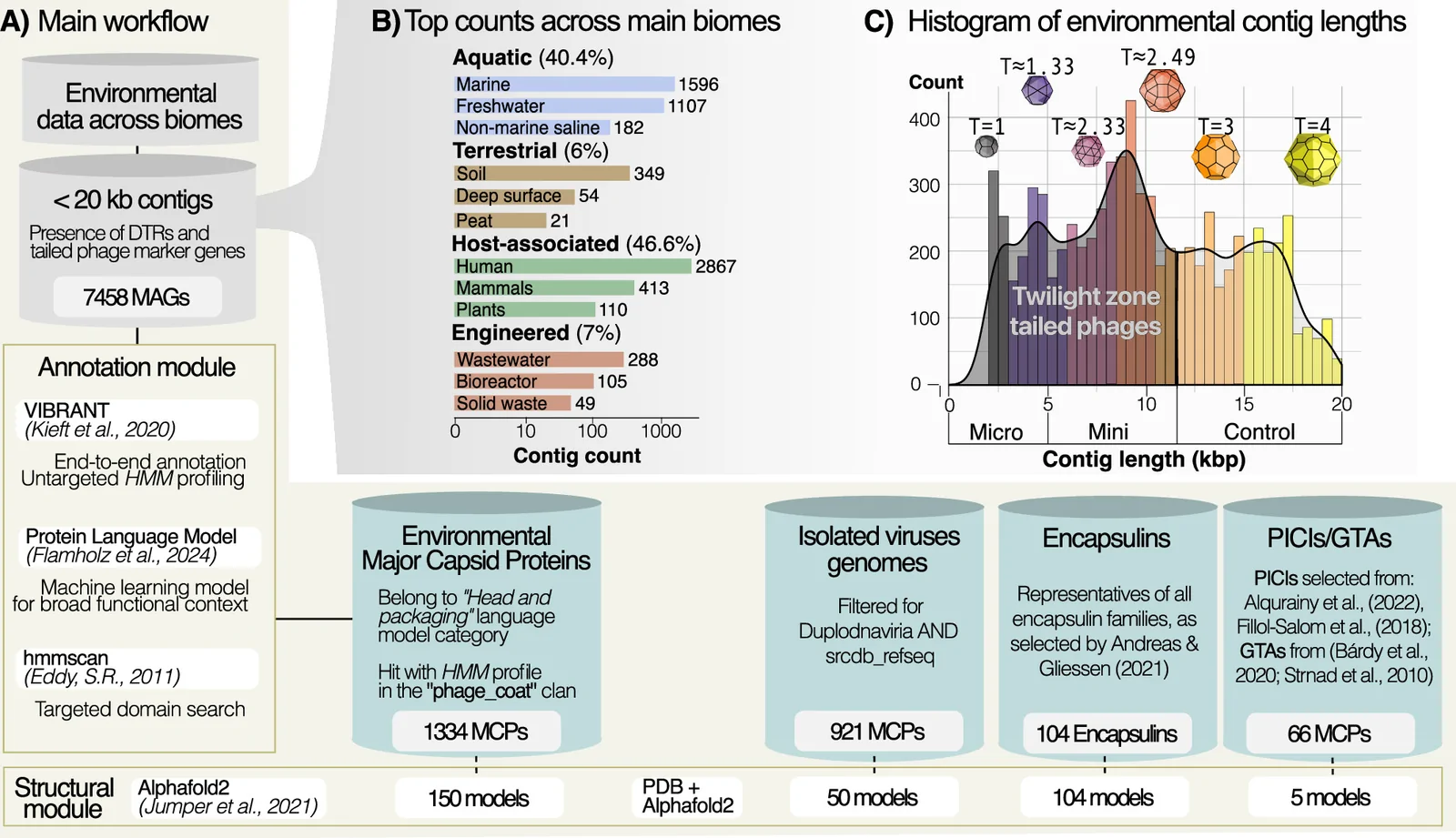
Discovery and comparison pipeline for twilight zone viruses. **A** The three main steps for environmental contigs are shown: filtering criteria, functional annotation, and identification of HK97-fold proteins. An overview of HK97-fold protein datasets is included. The structural module indicates the number of structures predicted with AlphaFold2 or obtained from the Protein Data Bank (PDB). **B** Number of contigs (log scale) of the three most frequent biomes of each Level 1 category. **C** Histogram of contig lengths. The solid line is a kernel density distribution with a bandwidth of 700 bp. The predicted T-numbers are shown above and include both canonical icosahedral architectures and non-canonical icosahedral lattices from the generalized icosahedral framework^48^, such as the trihexagonal (T ≈ 1.33), snub hexagonal (T ≈ 2.33), and rhombitrihexagonal (T ≈ 2.49) lattices, which incorporate minor capsid proteins. The Twilight Zone is defined as the region below 11.6 kbp, associated with the smallest isolated *Duplodnaviria* genome. It includes mini (5 kbp to 11.6 kbp) and micro (<5 kbp) contigs. Source data are provided as a Source Data file.

Here, we expanded the analysis to include samples across ecosystems (Fig. 2A, B and S1), refined the HK97-fold detection strategies, introduced a new network-based method to analyze the co-occurrence of HK97-fold sequence profiles and generated large-scale structural and sequence-based phylogenetic analyses. Our investigation identified 523 genomes with HK97-fold proteins below the genome length associated with the shortest HK97-fold-encoding genome of a phage isolate. Among these small genomes, 93 encoded HK97-fold proteins with encapsulin hidden Markov protein profiles. The newly identified HK97-fold proteins helped to reveal at least three transition events between encapsulins and HK97-fold viruses, suggesting bidirectional evolution. Further molecular and structural comparisons led us to propose that viral procapsids are the evolutionary nexus of genomic and biochemical HK97-fold compartments, and we discuss that modern procapsids could carry both genomic and biochemical functions. We conclude by proposing an evolutionary model of increasing complexity in early HK97-fold protein shells. We argue that they emerged as lower-symmetry biochemical compartments and that the evolution of specialized domains favored their expansion in size, thereby facilitating genome encapsidation.

## Results

### Key terminology

The following terms are summarized here to facilitate the interpretation of the results. Twilight zone: metagenomically assembled genomes with lengths < 11.6 kbp; the threshold is defined by the smallest genome from isolated HK97-fold viruses. Due to the biology of HK97-fold viruses and the prevalence of dsDNA data from standard sequencing methods, the investigated contigs were initially interpreted as dsDNA in origin, using kilobase pairs (kbp) units. Fringe zone: genomes with lengths between 11.6 kbp and 20 kbp, including both isolated and metagenomically assembled genomes; this region was used as a control. Micro genomes: twilight-zone genomes < 5 kbp. Mini genomes: twilight-zone genomes 5–11.6 kbp. Major capsid protein (MCP): the HK97-fold protein that builds the capsid shell in *Duplodnaviria*; in the context of viruses, we use MCP and HK97-fold protein equivalently. Structural domains: canonical structural regions of the HK97-fold defined from its three-dimensional configuration (Fig. 1A and Supplementary Fig.  S2). Sequence domains: domains identified in protein sequences by matches to hidden Markov model (HMM) profiles in the Pfam database (Supplementary Fig. S2); encapsulin domains are Pfam domains built from encapsulin shell proteins and are used here as encapsulin-like sequence signatures within HK97-fold proteins.

### Twilight viral genomes are frequent, and a fraction contain identifiable HK97-fold proteins

We identified 4730 putatively complete viral genomes in the twilight zone (< 11.6 kbp) from metagenomic datasets, which contained direct terminal repeats (DTRs) and characteristic *Duplodnaviria* genes. Twilight zone genomes represented 63% of the total viral contigs when including the control region (11.6 – 20 kbp), which contained 2728 viral contigs (Fig. 2C). The twilight zone genomes were frequent across biomes (Supplementary Fig. S1). However, the fraction of identified HK97-fold major capsid proteins (MCPs) was significantly smaller in the twilight zone (11% or 536 MCPs) than in the control region (29% or 798) (Supplementary Fig. S1 and Supplementary Table S1).

### MCPs from mini viral genomes were significantly enriched in encapsulin domains

**Fig. 3:**
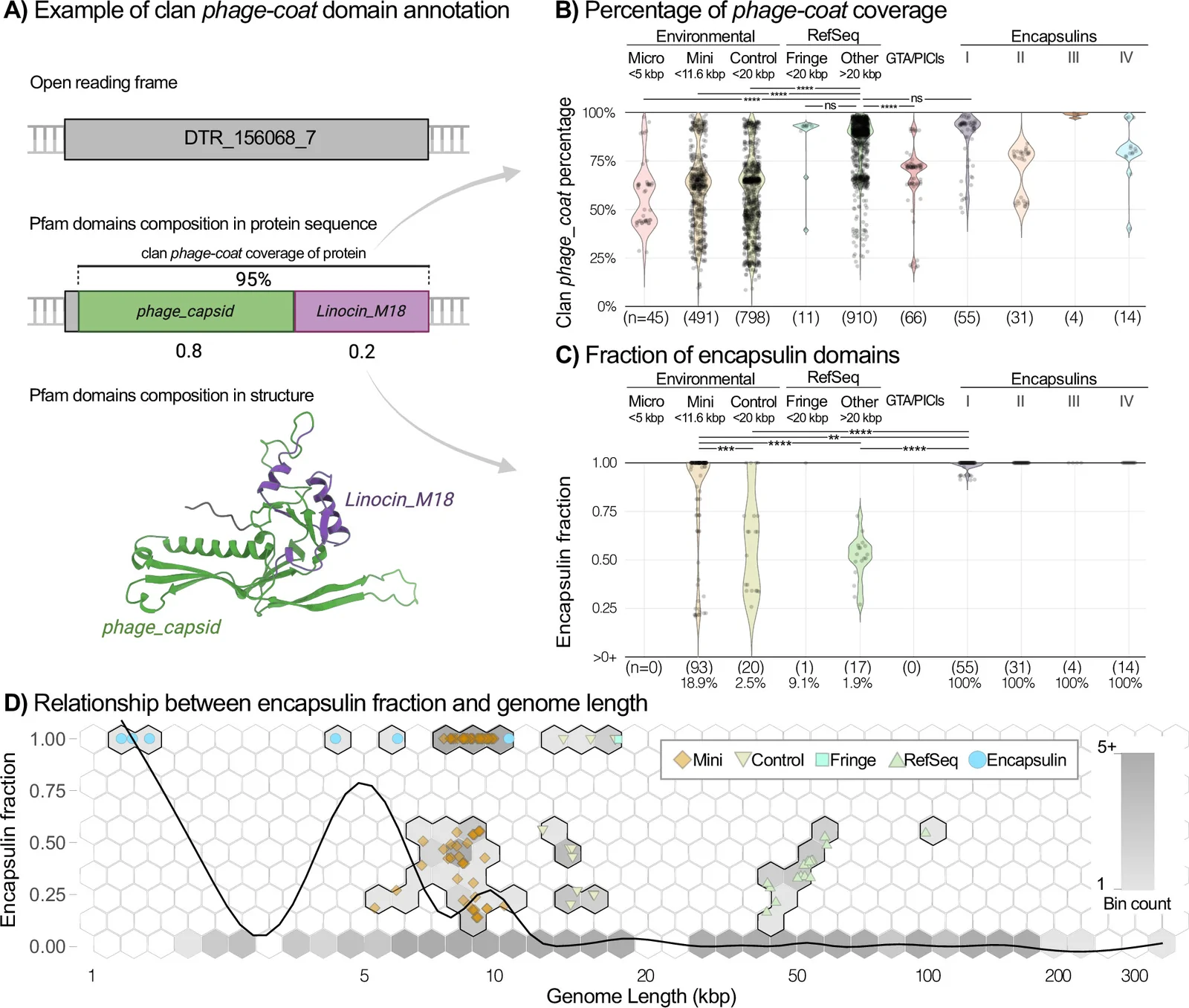
Encapsulin domain analysis across viral entities. **A** Schematic representation of the domain analysis. The *Phage-coat* sequence domains identified are displayed in different colors. The percentage of *Phage-coat* coverage is defined as the fraction of residues captured by the combined domains. The predicted structure is colored based on the region associated with each identified domain. **B** Phage-coat coverage of HK97-fold proteins from viral and encapsulin groups. Dots represent individual proteins, and group sizes (*n*) are shown in parentheses. RefSeq genomes are divided into fringe (11.6–20 kbp) and other (> 20 kbp) groups. Positive values from groups with *n* ≥ 5 were compared by Welch’s one-way ANOVA (*F*(8, 101.46) = 160.16, *P* = 5.48 × 10^−54^), followed by two-sided Games–Howell tests. For the displayed mini versus RefSeq comparison, adjusted *P* < 2.22 × 10^−16^ and Cliff’s *δ* = −0.65 (95% CI, − 0.69 to − 0.60). **C** Encapsulin domain fractions above zero. Positive values from groups with *n* ≥ 5 were compared by a Kruskal–Wallis test (*χ*^2^(5) = 90.02, *P* = 6.65 × 10^−18^), followed by two-sided Dunn tests with Benjamini–Hochberg adjustment. For the displayed mini versus control comparison, adjusted *P* = 2.22 × 10^−4^ and *δ* = 0.46 (95% CI, 0.22 to 0.67). Confidence intervals for *δ* were calculated from 2,000 bootstrap replicates. Complete pairwise results are provided in Supplementary Tables S3, S4. **D** Encapsulin domain fraction versus genome length. Symbols indicate groups, with colors matching (**B**, **C**). Encapsulin genome lengths are projected from their packaging capacity. The black curve is a descriptive generalized additive model using a thin-plate regression spline. Source data are provided as a Source Data file.

The working hypothesis of the study was that among HK97-fold viral proteins, those from the twilight zone would be more closely related to encapsulin proteins. This was tested by scanning HK97-fold proteins for the presence of encapsulin hidden Markov model (HMM) sequence domains (Fig. 3 and Supplementary Fig.  S2). The analysis mapped the HK97-fold’s hidden Markov model (HMM) profiles from the *Phage-coat* Pfam clan, which includes previously defined HMM profiles from viral and encapsulin HK97-fold proteins^84^. The analysis assessed the percentage of each HK97-fold protein sequence covered by *Phage-coat* profiles and the fraction of coverage associated with encapsulin domains (Fig. 3A).

The HK97-fold proteins from the NCBI Reference Sequence Database (RefSeq) group displayed the most extensive coverage, with a median of 95% (Fig. 3B). This was expected because the *Phage-coat* Pfam clan has been historically built from proteins of isolated viruses in RefSeq, whereas HK97-fold proteins from uncultured viruses are more divergent and therefore less likely to be fully captured by existing Pfam profiles. By contrast, the HK97-fold proteins that showed the greatest enrichment in encapsulin domains were from twilight-zone genomes, in agreement with our working hypothesis (Fig. 3C). Encapsulin domain hits concentrated in MCPs from mini genomes (5 kbp to 11.6 kbp). However, in micro genomes (<5 kbp), none of the 45 identified MCPs displayed encapsulin domains.

Nearly 19% of MCPs in mini genomes (93 out of 491) carried encapsulin domains, and in more than half of these cases (57 out of 93), encapsulin domains were their only *Phage-coat* profiles. These MCPs are encoded on contigs that pass CheckV viral filters, carry *Duplodnaviria* hallmark genes, and lack the operon context typical of encapsulins. We therefore treated them as viral MCPs with encapsulin-like domains, although some metagenomic elements may represent misclassified systems. The encapsulin-domain enrichment of MCPs from mini genomes was significantly higher than in the environmental control group (11.6 kbp to 20 kbp), where only 2.5% (20 out of 798) of the MCPs contained encapsulin domains, although 4 displayed an encapsulin domain fraction of 1 (Fig. 3C). In the RefSeq group, only 1.9% (17 out of 910) MCPs contained a trace of encapsulin domains. Their encapsulin-enriched MCPs, defined as those displaying one or more encapsulin domains, displayed encapsulin fractions below 0.75, and were found in genomes of lengths around ~ 50 kbp and ~ 100 kbp (Fig. 3D), except for one MCP with encapsulin fraction 1 from the deep-sea Asgard archaea virus VerdaV2 (NC_074645.1) with a genome length of 19.5 kbp belonging to the “fringe zone” (11.6–20 kbp)^85^. The RefSeq genomes encoded hallmark HK97-fold MCPs, portal proteins, and tail proteins. No HK97-fold proteins from the viral mobile elements (GTAs or PICIs) were found to contain encapsulin domains (Fig. 3C).

The prevalence of encapsulin domains across viral MCPs decreased with the viral genome length (Fig. 3D). The encapsulin-enriched twilight zone mini genomes (5 − 11.6 kbp) occupied the genome-length region between the projected genome capacities of the two largest encapsulin compartments, *T* = 3 and *T* = 4, which had projected genome capacities of ~ 5 kbp and ~ 10 kbp, respectively. The few encapsulin-enriched MCPs in the environmental control group had genome lengths ranging from 13 kbp to 19 kbp, which exceeded the maximum projected packaging capacity of known encapsulins. The even smaller fraction of viral genomes from RefSeq containing MCPs with encapsulin domains ranged from 19 kbp to 58 kbp, except for one virus with a 103 kbp genome (Fig. 3D).

### The dominant encapsulin-enriched viral genomes encode lysogenic genes and lack portal or tail proteins

**Fig. 4:**
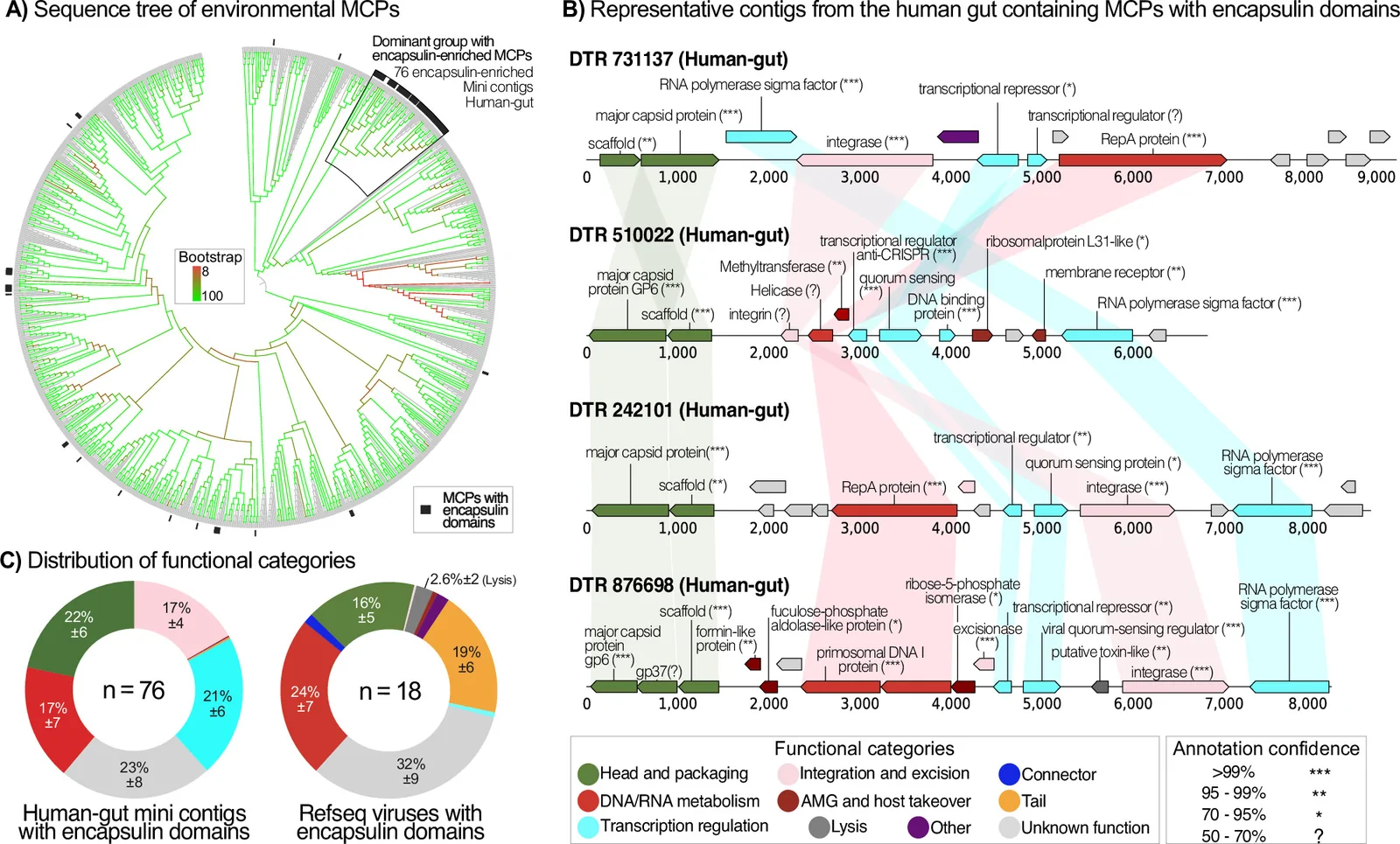
Genome composition of encapsulin-enriched mini viral entities. **A** Phylogeny of all identified environmental HK97-fold proteins (*n* = 1334). Black symbols adjacent to the tips indicate proteins containing encapsulin domains. Branches are colored by bootstrap support, from red (low) to green (high), with values ranging from 8 to 100. **B** Gene maps of representative mini genomes with encapsulin-enriched MCPs. The horizontal line represents the putative genome, and the numbers are the length in bp. The open reading frames are displayed as thick-colored arrows. The colors correspond to high-level genome functions listed in the legend at the bottom. The predicted function is included with its statistical significance in parentheses (see “Methods”). The label above each genome includes the contig identifier (DTR ######) and the biome of origin in parentheses. **C** Distribution of functional categories for the encapsulin-enriched mini viral group (left) and the encapsulin-enriched representatives from RefSeq (right) among viruses with encapsulin fractions. The colors correspond to the functional categories, as shown in panel B. The pie sections include the average percentages. Source data are provided as a Source Data file.

To assess whether encapsulin-enriched MCPs from environmental genomes formed a single group, a sequence phylogeny was conducted. The analysis indicated that encapsulin-enriched MCPs do not cluster into a single group and are instead distributed across 8 putative groups and 9 singletons (Fig. 4A). The largest group, recovered with bootstrap support (BS = 100), contained 58% (76) of the encapsulin-enriched MCPs. The genomic functional analysis of this large group revealed common characteristics (Fig. 4B). They originated from host-associated (human gut) samples. Their average genome length was 8.75 (±0.7) kbp. A substantial fraction of each genome encodes proteins of unknown function (~23% on average). Transcription-regulation genes were also frequent (e.g., sigma factors and transcriptional repressors), along with DNA/RNA metabolism genes such as RepA and DNA helicase. Notably, lysogeny functions were present, including integrase and, in some cases, excisionase. In contrast, no terminases or other packaging genes were detected. No portal, tail connector, or structural tail genes were found either. The only structural proteins identified were the HK97-fold MCP and scaffold protein. The only trace of a tail-related function among encapsulin-enriched genomes corresponded to a single open reading frame (DTR_497289_3), annotated as a tail tape measure protein.

Among the few *Duplodnaviria* viruses from the NCBI Reference Sequence Database (RefSeq) that contained encapsulin domains in their MCPs, 3 also encode lysogeny-related genes, suggesting they may be temperate. Two infect bacteria in the microbiomes of mammals, including humans, and one infects archaea from the order Poseidoniales. Their genomes encoded tail genes, comprising an average of 19( ± 6)% of the genome (Fig. 4C). The twilight viral genomes that did not encode encapsulin-enriched MCPs did not form a homogeneous group. However, tail genes were uncommon (Supplementary Table S2). Only 4.3% (23 out of 536) of twilight zone genomes with MCPs encoded tail genes, and the majority (20 out of 23) encoded just one tail gene. Among the control group, 12.2% (97 out of 798) encoded tail genes, but the control genomes containing encapsulin-enriched MCPs did not encode tail genes.

### Network and phylogenetic analyses identify independent virus-encapsulin transitions

To infer the evolutionary relationship between encapsulins and HK97-fold viruses, the HK97-fold proteins were analyzed using three complementary methods: co-occurrence of viral and encapsulin sequence profiles (Supplementary Fig. S2), structural phylogenetic analysis, and sequence-based phylogenetic analysis. The results suggest bidirectional evolution between HK97-fold viruses and encapsulins.

**Fig. 5:**
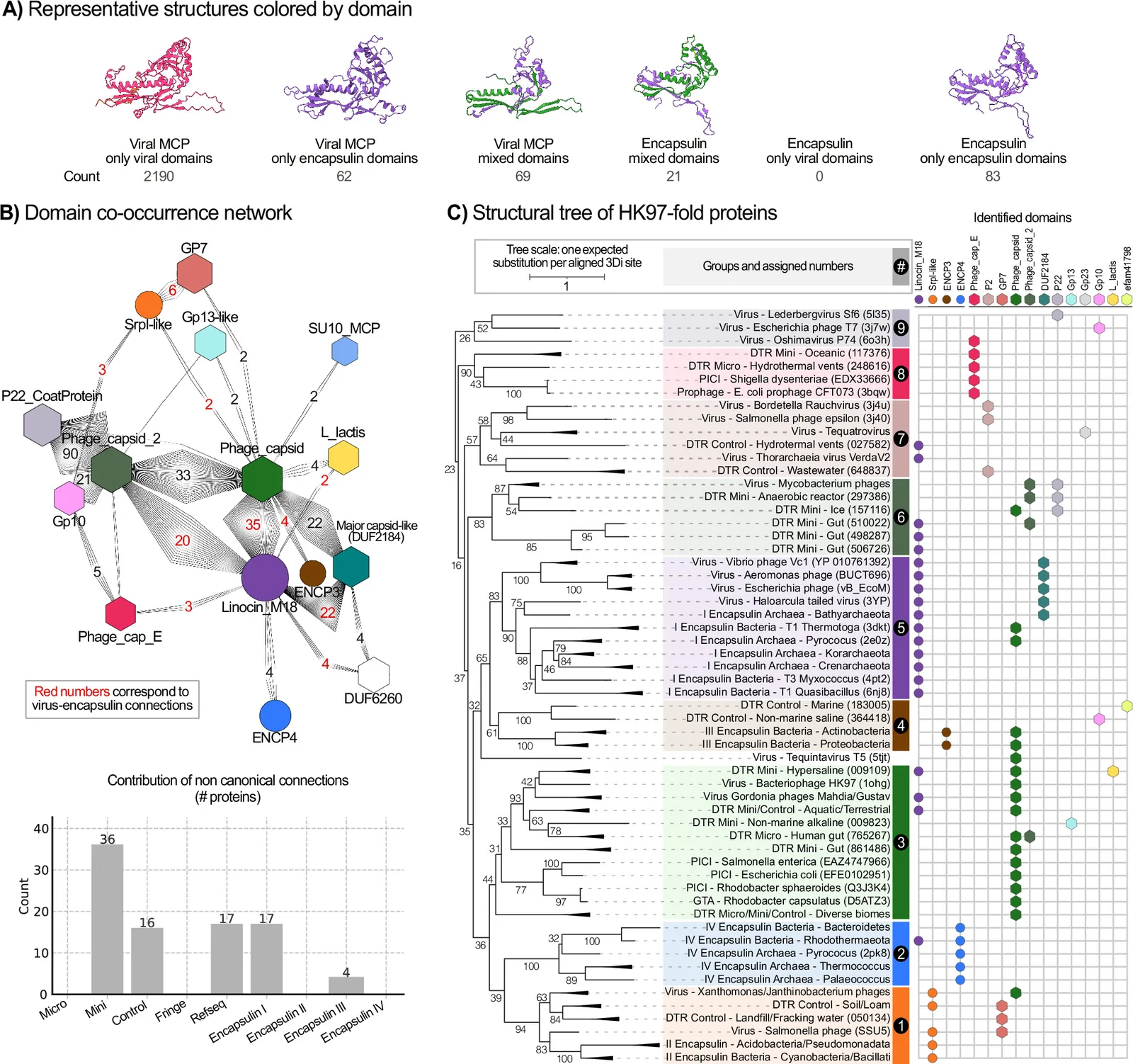
Structural phylogenetics and domain co-occurrence network. **A** Representative structures colored by Pfam domain. Six groups and their counts are shown. Each model is colored according to its Pfam profiles (see legend in **C**); mixed-domain proteins display multiple colors. **B** Pfam domain co-occurrence network. Nodes represent individual Pfam profiles (color-coded as in **C**); node size scales with total degree. Edges link profiles found in the same protein, with edge labels showing co-occurrence counts (red numbers denote counts of non-canonical virus–encapsulin links). Below, a bar plot breaks down the categories contributing to non-canonical connections (micro, mini, control, fringe, RefSeq, Encapsulins I–IV). **C** Structural tree of HK97-fold proteins. Maximum-likelihood midpoint-rooted tree of HK97-fold proteins. Branch lengths are reported in units of expected substitutions per aligned 3Di site, and node labels indicate bootstrap support values. Tip symbols represent the associated domains from the *Phage-coat* clan to the right, based on the color coding displayed in the legend on top (hexagon = virus-associated, circle = encapsulin-associated). Source data are provided as a Source Data file.

In the co-occurrence analysis, HK97-fold proteins were labeled as viral or encapsulin proteins based on their genomic origin. HK97-fold proteins encoded in viral genomes (environmental contigs passing the CheckV viral filters, or *Duplodnaviria* genomes, GTAs, or PICIs from RefSeq) were labeled as viral. Proteins encoded in curated encapsulin operons were labeled as encapsulins. A sub-category was assigned to each protein based on the identity of its HK97-fold sequence profiles in the *Phage-coat* Pfam clan prior to this study: viral domain (when displaying only viral domains) or encapsulin domain (when displaying only encapsulin domains). The term “mixed domains” was used when both viral and encapsulin domains were found in the same protein. The labeling of proteins yielded representatives for five out of the six possible groups (Fig. 5A). Viral HK97-fold MCPs (2321) formed one dominant group with only viral domains (*n* = 2190 or 94%) and two minor groups: one with mixed viral and encapsulin domains (*n* = 69 or 3%) and the other containing only encapsulin domains (*n* = 62 or 3%). Encapsulin HK97-fold proteins (104) formed two groups: the dominant group displayed only encapsulin domains (*n* = 83, 80%), and the smaller one combined viral and encapsulin domains (*n* = 21, 20%). No encapsulin HK97-fold proteins were found to contain only viral domains.

The co-occurrence network of *Phage-coat* domains revealed that MCPs from small uncultured genomes (control and mini) were necessary to fully connect the network of HK97-fold protein domains (Fig. 5B and S3). They also significantly increased the robustness of the existing non-canonical, that is, viral-encapsulin connections, present in the RefSeq subset (Supplementary Fig. S3). In the full network, the *Linocin_M18* and *Srpl-like* encapsulin domains exhibited, respectively, 86 and 11 non-canonical connections with viral domains (Fig. 5B). *Linocin_M18* (Family I encapsulins) was primarily connected to *Phage_capsid* domains (35 out of 86), while *Srpl-like* (Family II encapsulins) mostly connected to *GP7* domains (6 out of 11).

The large-scale structural phylogenetic analysis was performed with FoldTree, which was recently validated as an optimal approach for identifying distant homology among proteins sharing a conserved structure^86^. The analysis revealed three instances in which viral MCPs displayed a close evolutionary relationship with encapsulins. These instances corresponded to Structural Groups 1, 4, and 5 in the structural evolutionary analysis of HK97-fold MCPs (Fig. 5C). The Pfam sequence domains (HMM profiles) mentioned below were used for interpretation but were not included as an input for the phylogenetic inference. Encapsulin families (I–IV) are referred to according to the classification proposed by Andreas & Giessen (2021).

Structural Group 1 grouped Family II encapsulins with MCPs from terrestrial (soil/fracking) viral genomes from the environmental control group, and isolated phages infecting predominantly soil bacteria (*Xanthomonas* and *Janthinobacterium*) and host-associated bacteria (*Pseudomonas*). This mixed group was recovered with strong bootstrap support (BS = 94). The *Srpl-like* encapsulin domain and *GP7* virus domains were widespread in this group. Notably, the *SrpI-like* encapsulin domain was present in both encapsulins and viral MCPs, whereas the *GP7* viral domain was restricted to viral MCPs. At the sequence domain level, and under the most parsimonious interpretation, this pattern is consistent with the encapsulin-associated domain predating recruitment of the *GP7* viral domain along the viral lineage. Structural Group 4 clustered encapsulins from Family III, characteristic of soil bacteria, and environmental viral genomes from marine biomes. This association was recovered with moderate bootstrap support (BS = 61). Viral and encapsulin representatives in this group did not share a dominant sequence domain. Instead, the viral MCPs contained only virus-associated domains, including *efam41798*, which has been reported in a recently described MCP lineage^87^. Structural Group 5 clustered all Family I encapsulins with viral MCPs from phages infecting Gammaproteobacteria and an archaeal virus. This mixed group was strongly supported (BS = 83), and all members displayed *Linocin_M18* encapsulin domains. Notably, the archaeal virus Haloarcula tailed virus 3YP grouped with an archaeal encapsulin from Bathyarchaeota, rather than with other viruses.

The remaining structural groups were exclusive to either viruses or encapsulins. All Family IV encapsulins formed an encapsulin-exclusive group (Structural Group 2), displaying only encapsulin domains. Structural Groups 8 and 9 were the only viral-exclusive groups displaying only viral domains. MCPs with an encapsulin fraction of 1 were assigned to the virus-exclusive Structural Groups 6 and 7 (Fig. 5C); these groups displayed both virus and encapsulin domains.

The sequence-based phylogeny was performed as an independent method to identify viral-encapsulin transitions (Supplementary Fig. S4). Despite differing from the structure-based tree in overall topology, the sequence-based tree also recovered three mixed virus-encapsulin groups (Supplementary Fig. S5). Two of them were similar to the virus-encapsulin Structural Groups 1 and 5. As in Structural Group 4, the third virus-encapsulin sequence-based group comprised Family III encapsulins, but in this case, it contained a mix of reference and environmental viruses, including mini contigs with *Phage_capsid* domains rather than *Gp10* and *efam41798* domains. This group had stronger support (BS = 85) than Structural Group 4 (BS = 61). The sequence-based tree also recovered the virus-exclusive Structural Group 6, which contains virus and encapsulin domains. As in the structural tree, all Family IV encapsulins clustered together (Supplementary Fig. S6). Conversely, the viral-exclusive Structural Groups 7, 8, and 9 lacked counterparts in the sequence tree, likely reflecting the limited resolution of deeper relationships and the different information captured by structure- and sequence-based inference (Supplementary Fig. S6).

### Viral procapsids and encapsulins show close structural similarity

**Fig. 6:**
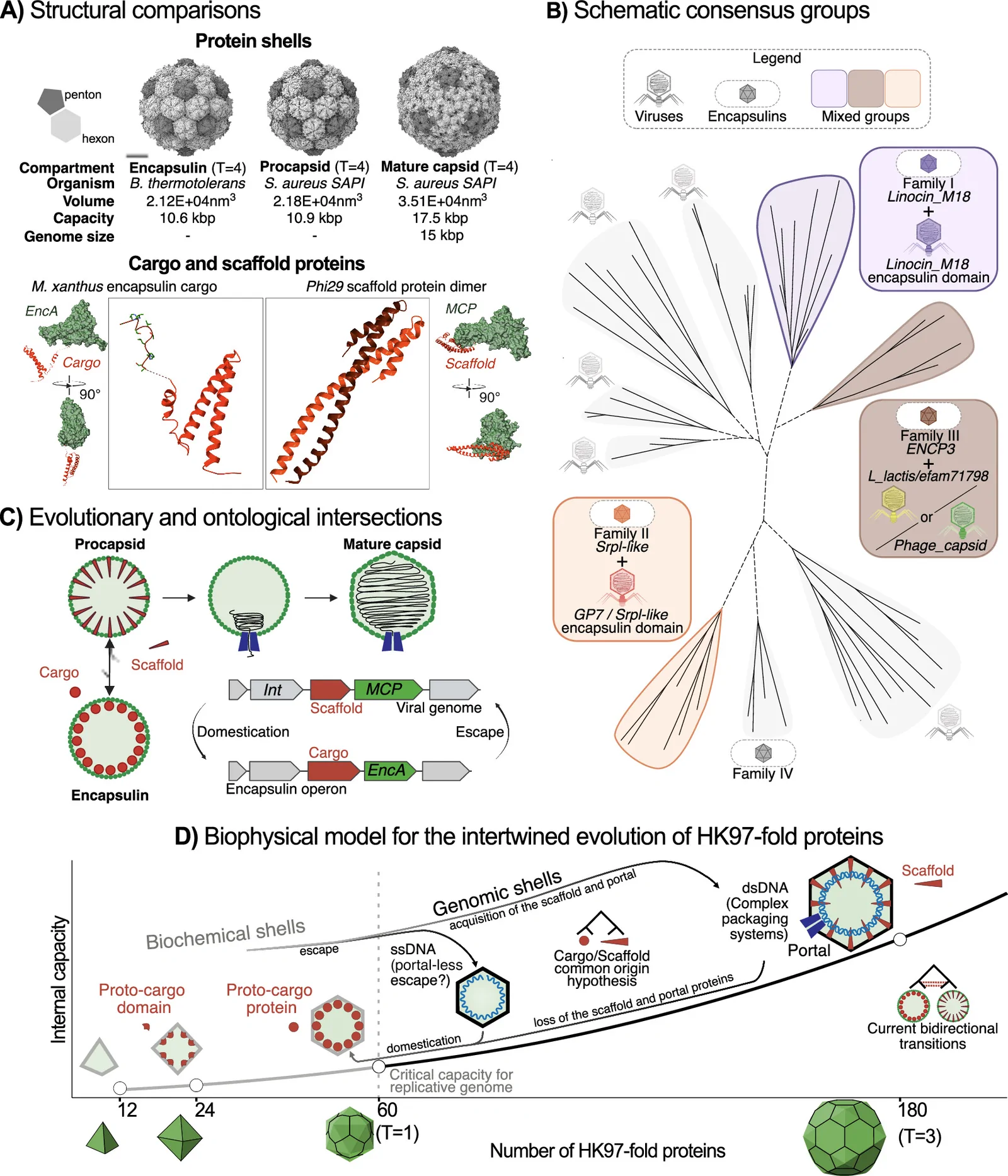
Model of HK97 fold protein evolution. **A** Structural comparisons of HK97-fold compartments (top) and cargo/scaffold comparisons (bottom). The structural resemblance between an encapsulin (PDB: 6NJ8) and a procapsid (PDB: 7RWZ) is highlighted, in contrast to an expanded mature capsid (PDB: 8VD4). This comparison emphasizes a key constraint in viral evolution: at the procapsid stage, the internal volume is insufficient to accommodate the genome that the mature capsid will encapsulate. Representative structures of the encapsulin cargo protein from *Myxococcus xanthus* (EncA; PDB 7S8T) and the bacteriophage Φ29 scaffold protein dimer (PDB 8F2M), each adopting an alpha-helical hairpin motif. **B** Schematic summary of multiple inferred transitions between viral MCPs and encapsulins. **C** Evolutionary and ontological intersections between viral MCPs and encapsulins. The procapsid is shown as a potential bifurcation point in HK97-fold evolution, illustrating how internal partners could bias the function of the compartment. **D** Proposed biophysical model of the origin and evolution of HK97-fold compartments. Internal capacity (y-axis; qualitative) is plotted against the number of HK97-fold proteins per shell (x-axis). Small, low-symmetry shells (12–24 subunits) are depicted as biochemical compartments whose volume is insufficient to enclose a self-replicating genome. Crossing a critical capacity allows *T* = 1 icosahedral shell (~ 60 subunits) to accommodate minimal genomes, most plausibly ssDNA, without a dedicated packaging system. Further increases in shell size, together with the emergence of scaffold proteins and portal-based packaging machinery, favor larger *T* = 3 dsDNA capsids (~ 180 subunits), linking biochemical and genomic regimes in HK97-fold evolution.

To infer the potential mechanisms underlying the bidirectional evolution of viruses and encapsulins, the structures of encapsulins were compared with those of viral HK97-fold capsids and their immature state, procapsids. This was possible thanks to the existence of reconstructed *T* = 4 architectures for encapsulins, capsids, and procapsids (Fig. 6A and Supplementary Fig. S7).

The analysis revealed that viral procapsids are more structurally similar to encapsulins than to the mature capsid state (Fig. 6A). The comparison included the encapsulin from *Bacillus thermotolerans* (PDB 6NJ8)^34^ and the procapsid and capsid structures assembled by *Staphylococcus aureus* Pathogenicity Islands (PDB 7RWZ and 8VD4)^29,88^. The encapsulin and viral HK97-fold protein sequences were poorly conserved, displaying a 14.4% identity over 473 aligned residues (23.9% similarity), yet their quaternary structures (protein shells) are strikingly similar. Both entities assemble into a quasi-spherical structure, exhibiting comparable inner diameters: ~ 322 Å for the encapsulin and ~ 330 Å for the procapsid. Their shells enclose a comparable volume (~ 2.15·10⁴ nm³) with a projected capacity of ~10.75 kbp. This capacity is 60% smaller than the internal volume (~ 3.51·10⁴ nm³) and packaging capacity (~ 15.2 kbp) observed in the mature capsid (Fig. 6A). The expansion in volume from the procapsid to the mature capsid stage is common among *Duplodnaviria* viruses (Supplementary Fig. S7) and facilitates scaffold protein release^26,61^.

Since encapsulins contain cargo proteins and procapsids contain scaffold proteins, cargo and scaffold proteins with available structures were compared. This included encapsulin cargos from *Bacillus thermotolerans* and *Myxococcus xanthus*, and scaffold proteins from phage P22 and phage Φ29. Global pairwise alignments were computed with EMBOSS Needleman–Wunsch^89^, revealing low overall sequence identity (4.4–18.0%) and similarity (6.9–30.9%), with the highest similarity observed for the *M. xanthus* cargo versus the Φ29 scaffold (Fig. 6A). However, common structural traits were identified (Fig. 6A and Supplementary Fig. S8). Both proteins are alpha-rich and contain an alpha-helical hairpin motif, although their orientation inside the capsid may differ (Fig. 6A). These results indicate structural parallels between cargo and scaffold proteins.

## Discussion

### Procapsid as an evolutionary nexus of HK97-fold compartments

The structural and sequence phylogenetic analyses of HK97-fold proteins revealed at least three mixed virus-encapsulin groups that, together with shared domains, support the intertwined evolution of encapsulins and viruses, as summarized in Fig. 6B. This bidirectional evolutionary paradigm is further supported by our observations indicating that HK97-fold protein domains, traditionally assigned to viruses and encapsulins, form a fully connected co-occurrence network, as revealed by the addition of HK97-fold proteins from small uncultured viruses (Fig. 5A, B and Supplementary Fig. S3). Taken together, these patterns indicate that modern HK97-fold viruses and encapsulins have evolved from each other multiple times, providing a scenario that has not been considered before for HK97-fold shells^42,90^.

Yet bidirectional or mixed ancestry between viruses and virus‑derived elements is not unprecedented, as multiple studies have documented gene exchange and recombination between viruses and genetic elements such as GTAs, plasmids, and related mobile elements^91–95^. For example, plasmids with replication initiator proteins (Reps) closely related to those of ssDNA viruses have repeatedly given rise to new virus lineages through the acquisition of single jelly-roll fold capsid protein genes, while some plasmids have been proposed to derive from viruses via the loss of the capsid proteins^93^. Thus, bidirectionality between HK97-fold viral and cellular compartments aligns with that observed in other viral capsid folds.

These parallels make it relevant to ask which molecular mechanisms could enable HK97-fold shells to transition repeatedly between viral and encapsulin states. One compelling possibility is that the procapsid state observed in HK97-fold viruses has facilitated such transitions (Fig. 6C). This hypothesis is based on the structural similarity of encapsulin and procapsid compartments (Fig. 6A), the co-assembly and structural parallels of cargo and viral scaffold proteins (Fig. 6C), and the presence of lysogenic genes in the subset of mini genomes encoding encapsulin-enriched HK97-fold proteins discovered in this study (Fig. 4). The presence of lysogeny-associated genes indicates the potential for genomic integration into host chromosomes^96,97^. Integration increases the opportunity for recombination between viral HK97-fold shells and host-encoded cargo-like proteins, favoring the emergence of encapsulin compartments from viral ancestors. The same setting could also facilitate recombination events that link encapsulin shells to scaffolding and portal genes, allowing encapsulin compartments to reacquire genome-packaging functions and move back toward a virus-like lifestyle. Together with the shared alpha-helical hairpin motif of cargo and scaffold proteins (Fig. 6A), these observations support a mechanistic model in which the internal protein in the HK97-fold shell may act as a switch between biochemical and genetic roles (Fig. 6C). The observation that the few isolated *Duplodnaviria* viruses containing encapsulin domains also encode lysogeny-related genes further supports this scenario. Additionally, encapsulins and procapsids form shells that are more porous and display bulgier capsomers (pentamers and hexamers) than mature capsids^98–101^. The pores enable encapsulins to transport reactants (ions or other molecules) necessary for biochemical functions^44,102,103^. In fact, the pores in procapsids have facilitated their use as biochemical reactors in engineering applications^100,104^. The parallels between viral procapsids and encapsulins suggest a flexible, bidirectional model in which the identity of the internal functional protein (cargo/scaffold) helps determine the role of an HK97-fold shell (Fig. 6C). If the internal protein is an enzymatic cargo, the compartment acts as a biochemical reactor, concentrating substrates and protecting intermediates. If it is a scaffold that favors the interaction with the portal proteins, the shell is instead expanded, the scaffold is released, and a dsDNA genome can be packaged. Thus, switching the identity of the internal protein may help determine if an HK97-fold compartment operates as a biochemical reactor (encapsulin-like) or a genome storage and delivery entity (virus-like). Under such a bidirectional regime, repeated transitions between these states are expected to blur a simple, single-origin signal for either purely biochemical or purely genetic HK97-fold compartments.

This virus-encapsulin transition is consistent with frameworks considering that viruses function as integral components of larger biological systems (holobionts), facilitating extensive genetic and functional exchanges with cellular hosts^105^. It is also compatible with the proposal of Koonin and Krupovic (2017) that many capsid proteins evolved repeatedly by recruitment from cellular enzymes and nanocompartment proteins. In this context, the cargo/scaffold proteins provide a natural candidate for enzyme-derived precursors, whose activities can be redeployed in viral or cellular settings depending on how they interact with HK97-fold shells. Given the similarities between encapsulins and procapsids, if viral genomes or the hosts encode cargo-related proteins that are co-assembled in procapsids, then procapsids could support biochemical functions during viral infections. It is not impossible, either, that extant scaffold proteins could retain biochemical functions. The observation that viral infections often generate empty capsids or procapsids^106,107^ makes this potential biochemical role amenable to experimental testing. Demonstrating such activities would broaden current views of capsids as purely protective shells and motivate a more nuanced picture of their contribution during infection.

### The DNA packaging possibilities in portal-less and non-tailed HK97-fold twilight viruses

The group of encapsulin-enriched mini genomes reported here lacked a recognizable portal, which is essential for the dsDNA genome packaging in all known *Duplodnaviria* viruses^108^ (Fig. 4). They also lacked the main proteins that assemble the tail apparatus, which is essential for translocating the dsDNA genome in prokaryotic *Duplodnaviria* and for associated HK97-fold mobile elements^28,108^. The lack of these genes raises the question of how these entities could replicate, pack, and deliver a dsDNA genome. One hypothesis is that these entities are satellites of other viral entities that carry the more common HK97-fold virus machinery.

The alternative hypothesis is that these twilight zone viruses deviate from the canonical replication and packaging strategy of *Duplodnaviria*. Several genomes encode proteins annotated as rolling-circle replication initiators (Rep/RepA-like). The proteins belong to the broader HUH endonuclease superfamily that includes plasmid and ssDNA viral replicases^93,109^, and RepA proteins participate in the plasmid-like phase of HK97-fold phages P4 and N15^110^. RepA proteins share the canonical HUH endonuclease mechanism of site‑specific nicking and subsequent strand transfer/ligation on ssDNA that can, in principle, couple rolling-circle replication to directed DNA translocation. In small ssDNA viruses, genome packaging occurs through 3-fold or 5-fold pores in the capsid, without a dedicated portal–tail complex^111–114^. The existence of small ssDNA viruses with capsids built from double jelly-roll proteins in lineages traditionally associated with dsDNA genomes^113–116^, together with chimeric viruses that combine circular ssDNA genomes with capsid proteins most similar to those of ssRNA viruses^117^, underscores that genome type and capsid fold can be decoupled. In this broader context, the co-occurrence of HK97-fold major capsid proteins and Rep/RepA-like initiators in portal-less, tail-less mini genomes supports the possibility that some of these entities might package circular ssDNA via a Rep-mediated mechanism. Thus, it is mechanically possible that some twilight-zone viruses could represent HK97-fold viruses with a ssDNA lifestyle. This hypothesis remains untested and will require experimental confirmation.

Either the need for helper viral elements or an alternative packaging and infection strategy could explain the lack of isolated twilight zone HK97-fold viruses described here. Among isolated HK97-fold viruses, satellite lifestyles seem more common in those with relatively short genomes (12 kbp to 20 kbp) and HK97-type mobile elements (11 kbp to 18 kbp)^118–121^. The presence of tail genes in a small fraction of twilight zone viruses may be a remnant of a more canonical tailed phage structure and replication, possibly in combination with helper viruses. Nonetheless, it is important to highlight that the characteristic small ssDNA viruses (< 6 kb) in the *Microviridae* family, which form tailless viruses, can generate a dynamic tail during infection^122,123^. Therefore, the few tail genes observed in the HK97-fold twilight viruses could operate similarly to dynamic tails instead of the canonical *Duplodnaviria* tails.

### The ancestry and evolution of HK97-fold compartments

Building on the procapsid nexus model (Fig. 6C), which could explain the bidirectional transition between viruses and encapsulins, and the possibility of ssDNA HK97-fold twilight viruses (Fig. 4), the final question concerns the ancestral form of HK97-fold compartments. The lack of an optimal rooting strategy, together with the low support values for the deep branches, prevents reliable inference of a virus-like or encapsulin-like ancestor and precludes strong claims of directionality based on the phylogenetic analyses. However, we speculate a biophysically grounded evolutionary model of increasing complexity to complement the unresolved relationships of the phylogenetic analyses (Fig. 6D). We propose that HK97-fold shells emerged as small, low-symmetry tetrahedral or octahedral shells. It has been observed that single-point mutations in the HK97-fold protein of the T = 3 (180-mer) encapsulin of *Myxococcus xanthus* can lead to the formation of T = 1 (60-mer) and a tetrahedral (36-mer) architecture^124^. Tetrahedral and octahedral capsids form local thermodynamic minima in biophysical models, and their reduced number of capsid proteins makes them kinetically advantageous^125–127^. Thus, the early evolution of tetrahedral or octahedral HK97-fold capsids is biophysically sound. However, such shells have a small internal volume; the structurally reconstructed tetrahedral HK97-fold shell displays an internal volume of ~ 300 nm^3^, which is ~ 6.5 times smaller than the equivalent T = 1 shells and 40 times less than the volume of the wild-type *T* = 3^124^. At quasicrystal densities, such a tetrahedral shell could accommodate 150 bp of dsDNA or 300 bases of ssDNA^43^. This could encode a protein, but it is at least three times smaller than the smallest DNA viral genome known^128,129^. However, there is evidence of biochemical compartments with volumes below 300 nm^3^ that enclose Fe^2+^ oxidation^130,131^, which is also present in encapsulins^42,132^. Thus, low-symmetry HK97-fold compartments would likely have carried out biochemical reactions before they could accommodate a genome. On the other hand, larger capsids are thermodynamically more favorable^127^. This, along with the formation of specialized biochemical domains in the interior-facing regions of HK97-fold compartments that later became cargo proteins (“proto-cargo” proteins), would have favored increases in size, yielding T = 1 capsids with sufficient volume to enclose minimal genomes (Fig. 6D). Consistent with patterns of protein domain evolution, catalytic domains within such ancestral proto-cargo proteins could then have emancipated as independent proteins, forming early encapsulin-like nanocompartments^133,134^. The internal *T* = 1 volume may have simultaneously favored the translocation of single-stranded DNA, as in other *T* = 1 capsid systems^111,112,114^, leading to early HK97-fold twilight zone viruses. Eventually, scaffold-like proteins may have favored further increases in capsid size and interactions with portal proteins, giving rise to the more complex dsDNA *Duplodnaviria* viruses (Fig. 6D). At the same time, because HK97-fold compartments appear to have repeatedly switched between encapsulin- and virus-states, our current sampling cannot determine whether HK97-fold compartments for genome packaging or biochemical roles emerged first, and recurrent transitions may have partially eroded the signature of the earliest steps. The speculative yet parsimonious biophysical scenario for the early evolution of HK97-fold compartments in Fig. 6D integrates the various elements identified in this study, providing an evolutionary model with specific predictions and testable hypotheses for future studies.

In conclusion, the relationship revealed between HK97-fold proteins from small viral genomes and encapsulins supports a change of paradigm in the evolution of HK97-fold compartments, favoring a bidirectional evolution in which the procapsid is the most likely nexus. The potential genomic and biochemical functions of procapsids make this system particularly interesting for studying the evolution of compartmentalization of metabolic reactions and genetic material in early life. In particular, the HK97-fold major capsid proteins displaying encapsulin-like domains in the twilight zone genome range provide a tractable set of candidates for follow-up theoretical and experimental work to probe their architecture, potential novel packaging strategies, and evolution of HK97-fold compartments.

## Methods

The links to access the Data Files referenced in the Methods and Results sections are available in the Supplementary Information file. Code and analysis workflows related to this study are available at https://github.com/luquelab/hk97-viruses-encapsulins-evolution.

### Identifying environmental HK97-fold viral genomes in the twilight zone

Small, complete HK97-fold viral genomes were identified from viral contigs containing direct terminal repeats (DTRs) in public environmental omics data (metagenomes, metatranscriptomes, and metaviromes) used in the viral pipeline CheckV^83^. This included over 14.4 billion contigs from IMG/M^135^, Mgnify^136^, human microbiomes^137–139^, and ocean virome^140^. The complete list of IMG datasets used in this study is provided in Data File S1. The standard hierarchical metagenomic biome classification schema was used^141^, available in Supplementary Data File S2. The assignment of contigs to biomes is provided in Supplementary Data File S3.

The bioinformatics pipeline CheckV estimated the completeness of viral genomes and removed non-viral regions from integrated proviruses^83^. The viral contigs selected for analysis (Data File S4) satisfied three criteria. First, the contigs contained a direct terminal repeat (DTR) window of at least 20 base pairs (bp), which was used as a bioinformatic proxy for a complete or closed viral genome^43,81–83^. Second, each selected genome contained at least one marker gene associated with tailed phages, including major capsid protein, portal protein, terminase, baseplate, or tail genes^25^. Open reading frames were identified using Prodigal v2.6.3 (option ‘-p’ for metagenome mode)^142^. Third, the contigs analyzed were filtered to have a length of 20 kbp or less. This criterion captured the twilight zone (0 kbp to 11.6 kbp) below the smallest isolated-tailed phage containing a major capsid protein, Salmonella phage astrithr^43,75^, and a control region above it (11.6 kbp and 20 kbp). The twilight zone was divided into micro genomes (<5 kbp), which had not been previously reported among HK97-fold entities, and mini genomes (5–11.6 kbp).

The capsid phenotype was predicted using the genome-to-capsid model for HK97-fold protein shells^22,43^. Predictions were interpreted within the generalized icosahedral framework of Twarock and Luque (2019), which includes both canonical T = 1 icosahedral capsids and non-canonical architectures associated with alternative lattices that accommodate minor proteins, such as the trihexagonal (*T* = 4/3), snub hexagonal (*T* = 7/3), and rhombitrihexagonal (*T* = 5/2) lattices. In the absence of minor proteins, these non-canonical icosahedral capsids should be interpreted instead as elongated *T* = 1 capsids^143,144^ or shrunken T = 3 capsids, consistent with a reported gene transfer agent (GTA) HK97-fold capsid^28^.

### Genome annotation

The open reading frames (Data File S5) were annotated in three complementary steps (Fig. 2A). First, high-level functional categories were predicted using the viral protein families-protein language model, VPF-PLM^87^. The VPF-PLM had been trained on the prokaryotic virus remote homologous groups (PHROGs) database^145^. Functions were assigned when obtaining likelihood scores greater than 60%; the highest-ranked function was selected. The method achieves a weighted F1-score (harmonic mean of the precision and recall) of 85% at this threshold for assigning correct high-level functional categories to viral proteins validated on ocean virome sequences (see annotations in Supplementary Data File S6).

Second, the VIBRANT (Virus Identification By iteRative aNnoTation) tool, which combines neural networks and hidden Markov models (HMMs)^146^, was applied to predict specific functions for each open reading frame. Version 1.2.1 of VIBRANT was executed with default parameters, which included curated HMM profiles from three major databases: KEGG (March 2019 release), Pfam (v32), and Virus Orthologous Groups (VOG; release 94) (see annotations in Data File S7).

Third, all predicted open reading frames, regardless of prior functional assignments, were scanned with hmmscan against the Pfam database^147^, clan CL0373 (“*Phage-coat*”), which contains curated HMM profiles for domains characteristic of the HK97 fold. The clan comprised 17 HMM profiles, including viral capsid protein domains (14) and encapsulin shell domains (3). An additional viral capsid protein HMM profile (41798) from the efam database^148^ was included, as it represents a recently proposed HK97-fold MCP lineage identified using protein language models^87^. Hits were accepted at a cutoff of E-value < 10⁻³. The HMM profile list and annotations are provided in Data File S8.

Finally, HHpred^149^ was used for targeted manual curation and contextual annotation of selected genomes of interest. We examined 639 open reading frames from 60 genomes, focusing primarily on proteins that remained unannotated by the automated methods or on genes from genomes showcased in figures. These ORFs largely corresponded to accessory or hypothetical proteins. HHpred searches were run against Pfam-A (v37), NCBI Conserved Domains (v3.18), and PHROGs (v4) with default server parameters and a MAC realignment threshold of 0.3. The Protein Data Bank (PDB) library was included to enhance structural evolutionary insights. Hits below 20% probability were discarded. For each open reading frame, the top hit was selected unless a longer hit of comparable significance was present. Functions were labeled according to statistical confidence: very high (> 99%, ***), high (95–99%, **), moderate (70–95%, *), and low (50–70%,?). Annotations with a probability below 50% were considered hypothetical. Manual annotations took precedence over automated assignments when they conflicted. The HHpred annotations are provided in Supplementary Data File S9.

#### Clustering of environmental major capsid proteins

To test whether environmental major capsid proteins form a single group or multiple distinct groups, the amino-acid sequences of the environmental HK97-fold proteins (Supplementary Data File S10) were clustered using a maximum-likelihood tree. The multiple sequence alignment was performed using MAFFT v6.240^150^. The alignment of conserved cores was optimized by the L-INS-i algorithm, which adds a generalized gap cost to accommodate variable regions and is recommended for proteins with one conserved domain and variable flanking regions, matching the architecture of HK97-fold MCPs^151^. The alignment was refined, and artifacts were reduced by increasing the number of iterative cycles up to 1000 using the --maxiterate parameter. Gap penalties were adjusted with gap thresholds of 0.05 and 0.15, which are standard when aligning highly variable viral proteins^93^. The phylogenetic inference was performed using maximum likelihood as implemented in IQ-TREE 2 v2.3.6^152^. The best-fit substitution model was based on the Bayesian Information Criterion (BIC) and obtained with ModelFinder, integrated into IQ-TREE 2. The tree was inferred with ultrafast bootstrap approximation using 1000 replicates, and the default consensus tree was generated. The output files, including the maximum-likelihood tree, log file, and likelihood distances, are provided in Supplementary Data File S11. The tree was visualized and annotated using iTOL v6^153^. The groups derived from the phylogeny were explored by partitioning the tree into groups using TreeCluster v1.0.3^154^, which identifies biologically meaningful groups based on tree distances. Clustering was performed using the avg_clade method, which groups leaves so that the average pairwise distance between leaves does not exceed a specified threshold while ensuring each cluster forms a clade. A threshold of 0.001 was applied to identify highly specific groupings.

### Comparative analysis across HK97-fold proteins

#### Reference HK97-fold protein database

The HK97-fold proteins obtained from environmental samples were compared with a reference database composed of HK97-fold proteins from curated sources, including viral genomes from NCBI RefSeq, viral-related mobile elements (gene transfer agents, GTAs, and phage-inducible chromosomal islands, PICIs), and encapsulins (Supplementary Data Files S12, S13). Viral MCP sequences belonging to the *Duplodnaviria* group were retrieved from the NCBI Viral Genomes Resource^155^ in December 2023 using the Boolean query (“MCP” OR “Major Capsid Protein”) AND “viral” NOT “human” AND “refseq”, filtering to select only those belonging to complete genomes. These sequences were further confirmed to contain HK97-fold domains using hmmscan against the Pfam clan CL0373 (“*Phage-coat*”) with an E-value cutoff < 10⁻³. The resulting validated sequences constituted the final reference dataset. Using NCBI identifiers reported in related publications,^156–158^, MCP sequences from 66 PICIs^156–158^, and 1 GTA^28^ were retrieved. The set of encapsulin sequences was obtained from a recent study that expanded the encapsulin universe^42^. In total, 104 encapsulin proteins were retrieved, ensuring representation across different phyla. These correspond to the four encapsulin families: family I (55 proteins), family II (31), family III (4), and family IV (14).

#### Estimating packaging capacity of encapsulins

To compare the packaging capacities of viral entities and encapsulins, the internal volume of encapsulins forming *T* = 1 (PDBs: 3DKT, 6X8T), *T* = 3 (4PT2, 2E0Z), and *T* = 4 (6NJ8) protein shells was measured using the Blob and Surface tools in ChimeraX^159^. The encapsulin structure was built from the asymmetric unit by applying icosahedral symmetry operations; a density map was generated, and the contour level was adjusted to define the capsid boundaries. The Blob Picker tool selected the inner capsid surface and measured the enclosed volume (Supplementary Fig. S9). The encapsulin packaging capacity was projected using the average density of double-stranded DNA in tailed phage capsids, ~0.5 bp/nm^3,^^43^^,^^160,161^.

#### Pfam domain analysis

The HK97-fold hidden Markov model (HMM) profiles captured in the *Phage-coat* Pfam clan (CL0373) were mapped to the major capsid protein sequences in the environmental groups (micro, mini, and control) and the reference HK97-fold database. For each protein, the *Phage-coat* coverage was calculated as the number of residues related to the non-overlapping Pfam domains identified divided by the protein’s amino-acid sequence length. The encapsulin enrichment of a protein was obtained as the fraction of the *Phage-coat* coverage associated with a Pfam encapsulin domain. The list of identifiers corresponding to contigs with encapsulin enriched MCPs is included in the Supplementary Information Data File S13. This domain analysis enabled the identification of viral MCPs exhibiting Pfam domains typically associated with encapsulins. The difference in *Phage-coat* coverage between groups was assessed using Welch’s one-way ANOVA, followed by Games–Howell post hoc pairwise comparisons to accommodate unequal variances and unequal sample sizes. Pairwise significance annotations and Supplementary Table S3 report Games–Howell adjusted p-values. Because the distribution of encapsulin fraction showed strong non-normality with many tied values at 100%, group differences were tested using Kruskal–Wallis, followed by Dunn’s post hoc pairwise comparisons with Benjamini–Hochberg correction. Pairwise adjusted p-values are reported in Supplementary Table S4. To visualize the relationship between encapsulin domain presence and genome length, we fitted a generalized additive model using a thin-plate regression spline for the smooth term. The response variable was the encapsulin fraction, and the predictor was the log10-transformed genome length. The smoother was used only as a descriptive aid and was not used for hypothesis testing.

#### Full compartment comparisons

To test whether encapsulin compartments resemble viral procapsids, we quantitatively compared representative *T* = 4 shells. We analyzed an encapsulin from *Bacillus thermotolerans* (PDB 6NJ8) and the *Staphylococcus aureus* Pathogenicity Island system, using its procapsid (PDB 7RWZ) and mature capsid structures (PDB 8VD4). Internal volumes were measured in ChimeraX using surface/volume tools by defining the enclosed lumen for each assembled shell, and packaging capacities were estimated assuming a dsDNA packing density of ~0.5 kbp/nm³ (Supplementary Fig. S9). Representative internal partner structures were compared using an encapsulin cargo protein (EncA; PDB 7S8T) and the bacteriophage Φ29 scaffold protein dimer (PDB 8F2M).

### Evolutionary analyses of HK97-fold proteins

#### Protein-domain co-occurrence analysis

Proteins containing more than one *Phage-coat* Pfam domain were used to build a co-occurrence network using the Python NetworkX library^162^. Each node corresponded to a distinct Pfam domain. Edges were drawn between nodes (Pfam domains) co-occurring in the same protein. The edges were categorized as canonical when connecting viral-viral or encapsulin-encapsulin Pfam domains and non-canonical when connecting viral-encapsulin Pfam domains. The placement of the nodes was determined with a spring layout algorithm. Nodes with a larger degree of connectivity (number of connections) were positioned closer to each other. The algorithm adjusted the position of the nodes iteratively until equilibrium was reached, minimizing the overall spring tensions in the network. Node size was proportional to that node’s degree of connectivity, scaled between 100 and 500 units. The networks for RefSeq, control, mini, and micro subsets were compared (Supplementary Fig. S3).

#### Structure-based phylogeny

Viral phylogenetic analyses based on fold-centric structural comparisons have been used to elucidate deep evolutionary relationships among viruses and cells^163^. Due to the lack of protein sequence similarities, the evolutionary relationships of HK97-fold proteins were primarily inferred from their protein structures. First, HK97-fold candidates were selected. This included 220 major capsid proteins (MCPs) from environmental contigs, ensuring representation across the length-based groups of twilight viral genomes (micro and mini) and control environmental viral contigs. This set included 113 MCPs enriched in encapsulin domains, indicative of the presence of ≥ 1 encapsulin domain. An additional set of 147 MCPs was selected from the reference HK97-fold database. This included 16 proteins whose structure had been obtained experimentally and deposited in the Protein Data Bank (PDB), and had been used in prior evolutionary studies^15^, 14 MCPs from isolated viruses encoding encapsulin-enriched HK97-fold domains identified in this study, 6 encapsulin shell proteins with available structures in the PDB, encompassing families I, II, and IV, 4 MCPs from PICIs, and 1 MCP encoded in a gene transfer agent (GTA). Second, for protein structures not available in the PDB, we predicted them using AlphaFold2 in ColabFold^164^. Structures were evaluated using the pLDDT (predicted Local Distance Difference Test) confidence metric. Folded protein structures scoring above 40 were retained for phylogenetic analysis. The empirical and predicted protein structures, including the pLDDT scores, are provided in Data File S14. Third, the structural phylogenetic tree for the HK97-fold proteins was constructed using FoldTree, which provides an alternative to sequence-based phylogenetic methods for distantly related proteins that share a conserved structure^86,165^. FoldTree utilizes Foldseek-derived 3Di encodings^166^ to represent local tertiary geometry as residue-level structural states, providing residue-level structural features as an alternative to global structural metrics. The resulting 3Di alignment was analyzed in IQ-TREE under a maximum-likelihood framework using empirical 3Di-state frequencies and allowing a fraction of invariant sites (F + I), with 3000 bootstrap replicates. The structural tree file is available in Data File S15. Given the absence of an appropriate external outgroup for HK97-fold proteins, the resulting structural tree is displayed midpoint-rooted for visualization.

#### Sequence-based phylogeny

To provide an independent validation of the evolutionary patterns identified through structural comparisons, we conducted a standard amino-acid sequence phylogenetic analysis using the same set of HK97-fold proteins included in the structural tree. Protein sequences were aligned using MAFFT (v7.520) with the L-INS-i algorithm, which is suitable for aligning globally similar proteins that may harbor modular rearrangements, such as domain insertions or deletions. MAFFT internally applied automated gap penalties and iterative refinement procedures. The alignment was trimmed using TrimAl (v1.4.rev15) with the “gappyout” parameter to remove poorly aligned positions while preserving informative regions. Maximum-likelihood inference was performed in IQ-TREE (v2.3.6) using ModelFinder for substitution model selection under the Bayesian Information Criterion. Branch support was calculated using ultrafast bootstrap approximation with 1000 replicates. Because no clear external outgroup could be defined for the HK97-fold dataset, the maximum-likelihood tree was rooted using midpoint rooting. The resulting tree is available in Supplementary Fig. S4 and Supplementary Data File S16.

### Reporting summary

Further information on research design is available in the Nature Portfolio Reporting Summary linked to this article.

## Supplementary information

Supplementary Information

Reporting Summary

Transparent Peer Review file

## Source data

Source Data

## Data Availability

Source data are provided with this paper (Source Data file). The data are also available as a permanent repository in Figshare, as indicated below. The environmental viral genomes and associated biome metadata generated and compiled in this study (Supplementary Data Files S1–S4) have been deposited in Figshare under https://doi.org/10.6084/m9.figshare.30943028. The predicted open reading frames and protein annotation data (Supplementary Data Files S5–S9) have been deposited in Figshare under https://doi.org/10.6084/m9.figshare.30948863. The HK97-fold sequence, structure, and phylogenetic analysis data (Supplementary Data Files S10–S17) have been deposited in Figshare under https://doi.org/10.6084/m9.figshare.30948881. Public reference data used in this study are available from the NCBI Viral Genomes Resource, NCBI RefSeq, and the Protein Data Bank; the relevant identifiers are provided in the Supplementary Information. Links to Supplementary Data Files S1–S17 are also provided in the Supplementary Information. Source data are provided with this paper. Protein Data Bank accession codes cited in the main text of this study: 3DKT (*Thermotoga maritima* encapsulin *T* = 1 shell); 6X8T (apo-SrpI encapsulin complex from *Synechococcus elongatus* PCC 7942 T = 1 shell); 4PT2 (*Myxococcus xanthus* encapsulin protein *T* = 3 shell); 2E0Z (*Pyrococcus furiosus* encapsulin T = 3 shell); 6NJ8 (*Bacillus thermotolerans* encapsulin, *T* = 4 shell); 7RWZ (*S. aureus* Pathogenicity Island 1 procapsid); 8VD4 (*S. aureus* Pathogenicity Island 1 mature capsid); 7S8T (*Myxococcus xanthus* encapsulin cargo protein EncA); 8F2M (bacteriophage Φ29 scaffold protein dimer). Additional PDB accession codes referenced in the Supplementary Information and used in the structural phylogenetics analyses are provided in Data File S14 [https://doi.org/10.6084/m9.figshare.30948881]. Source data are provided in this paper.

## Code availability

The code and analysis workflows used in this study are available in the GitHub repository at https://github.com/luquelab/hk97-viruses-encapsulins-evolution and are permanently archived as version 1.0.0 on Zenodo at https://doi.org/10.5281/zenodo.20802501.

## References

[1] Gilbert, W. Origin of life: The RNA world. *Nature* **319**, 618–618 (1986).

[2] Pressman, A., Blanco, C. & Chen, I. A. The RNA world as a model system to study the origin of life. *Curr. Biol.* **25**, R953–R963 (2015). 10.1016/j.cub.2015.06.016 26439358

[3] Kauffman, S. A. Cellular homeostasis, epigenesis and replication in randomly aggregated macromolecular systems. *J. Cybern.* **1**, 71–96 (1971).

[4] Smith, E. & Morowitz, H. J. Universality in intermediary metabolism. *Proc. Natl. Acad. Sci. USA* **101**, 13168–13173 (2004). 10.1073/pnas.0404922101 PMC516543 15340153

[5] Takagi, Y. A., Nguyen, D. H., Wexler, T. B. & Goldman, A. D. The coevolution of cellularity and metabolism following the origin of life. *J. Mol. Evol.* **88**, 598–617 (2020). 10.1007/s00239-020-09961-1 PMC7445158 32809045

[6] Wächtershäuser, G. The origin of life and its methodological challenge. *J. Theor. Biol.* **187**, 483–494 (1997). 10.1006/jtbi.1996.0383 9299293

[7] Becerra, A., Delaye, L., Islas, S. & Lazcano, A. The very early stages of biological evolution and the nature of the last common ancestor of the three major cell domains. *Annu. Rev. Ecol. Evol. Syst.* **38**, 361–379 (2007).

[8] Preiner, M. et al. The future of origin of life research: bridging decades-old divisions. *Life* **10**, 20 (2020). 10.3390/life10030020 PMC7151616 32110893

[9] Vincent, L. et al. Chemical ecosystem selection on mineral surfaces reveals long-term dynamics consistent with the spontaneous emergence of mutual catalysis. *Life* **9**, 80 (2019). 10.3390/life9040080 PMC6911371 31652727

[10] Cornell, C. E. et al. Prebiotic amino acids bind to and stabilize prebiotic fatty acid membranes. *Proc. Natl. Acad. Sci. USA* **116**, 17239–17244 (2019). 10.1073/pnas.1900275116 PMC6717294 31405964

[11] Mansy, S. S. & Szostak, J. W. Reconstructing the emergence of cellular life through the synthesis of model protocells. *Cold Spring Harb. Symp. Quant. Biol.* **74**, 47–54 (2009). 10.1101/sqb.2009.74.014 19734203

[12] Segré, D., Ben-Eli, D., Deamer, D. W. & Lancet, D. The lipid world. *Orig. Life Evol. Biosphere* **31**, 119–145 (2001). 10.1023/a:1006746807104 11296516

[13] Caetano-Anollés, G., Wang, M., Caetano-Anollés, D. & Mittenthal, J. E. The origin, evolution and structure of the protein world. *Biochem. J.* **417**, 621–637 (2009). 10.1042/BJ20082063 19133840

[14] Duda, R. L. & Teschke, C. M. The amazing HK97 fold: versatile results of modest differences. *Curr. Opin. Virol.* **36**, 9–16 (2019). 10.1016/j.coviro.2019.02.001 PMC6626583 30856581

[15] Krupovic, M. & Koonin, E. V. Multiple origins of viral capsid proteins from cellular ancestors. *Proc. Natl. Acad. Sci. USA* **114**, E2401–E2410 (2017). 10.1073/pnas.1621061114 PMC5373398 28265094

[16] Giessen, T. W. Encapsulins: microbial nanocompartments with applications in biomedicine, nanobiotechnology and materials science. *Curr. Opin. Chem. Biol.* **34**, 1–10 (2016). 10.1016/j.cbpa.2016.05.013 27232770

[17] Suhanovsky, M. M. & Teschke, C. M. Nature׳s favorite building block: Deciphering folding and capsid assembly of proteins with the HK97-fold. *Virology* **479–480**, 487–497 (2015). 10.1016/j.virol.2015.02.055 PMC4424165 25864106

[18] Sutter, M. et al. Structural basis of enzyme encapsulation into a bacterial nanocompartment. *Nat. Struct. Mol. Biol.* **15**, 939–947 (2008). 10.1038/nsmb.1473 19172747

[19] Wikoff, W. R. et al. Topologically Linked Protein Rings in the Bacteriophage HK97 Capsid. *Science* **289**, 2129–2133 (2000). 10.1126/science.289.5487.2129 11000116

[20] Cobián Güemes, A. G. et al. Viruses as Winners in the Game of Life. *Annu. Rev. Virol.* **3**, 197–214 (2016). 10.1146/annurev-virology-100114-054952 27741409

[21] Koonin, E. V., Kuhn, J. H., Dolja, V. V. & Krupovic, M. Megataxonomy and global ecology of the virosphere. *ISME J.* **18**, wrad042 (2024). 10.1093/ismejo/wrad042 PMC10848233 38365236

[22] Lee, D. Y. et al. Predicting the capsid architecture of phages from metagenomic data. *Comput. Struct. Biotechnol. J.* **20**, 721–732 (2022). 10.1016/j.csbj.2021.12.032 PMC8814770 35140890

[23] Hendrix, R. W. Bacteriophage HK97: Assembly of the Capsid and Evolutionary Connections. in *Advances in Virus Research* (Academic Press, 2005). 10.1016/S0065-3527(05)64001-8 16139590

[24] Wikoff, W. R. et al. Time-resolved molecular dynamics of bacteriophage HK97 capsid maturation interpreted by electron cryo-microscopy and X-ray crystallography. *J. Struct. Biol.* **153**, 300–306 (2006). 10.1016/j.jsb.2005.11.009 16427314

[25] Koonin, E. V. et al. Global organization and proposed megataxonomy of the virus world. *Microbiol. Mol. Biol. Rev.* **84**, e00061–19 (2020). 10.1128/MMBR.00061-19 PMC7062200 32132243

[26] Hua, J. et al. Capsids and genomes of jumbo-sized bacteriophages reveal the evolutionary reach of the HK97 fold. *mBio* **8**, e01579–17 (2017). 10.1128/mBio.01579-17 PMC5646251 29042498

[27] Pietilä, M. K. et al. Structure of the archaeal head-tailed virus HSTV-1 completes the HK97 fold story. *Proc. Natl. Acad. Sci. USA* **110**, 10604–10609 (2013). 10.1073/pnas.1303047110 PMC3696780 23733949

[28] Bárdy, P. et al. Structure and mechanism of DNA delivery of a gene transfer agent. *Nat. Commun.* **11**, 3034 (2020). 10.1038/s41467-020-16669-9 PMC7296036 32541663

[29] Dearborn, A. D. et al. Competing scaffolding proteins determine capsid size during mobilization of Staphylococcus aureus pathogenicity islands. *Elife* **6**, e30822 (2017). 10.7554/eLife.30822 PMC5644958 28984245

[30] Strnad, H. et al. Complete genome sequence of the photosynthetic purple nonsulfur bacterium Rhodobacter capsulatus SB 1003. *J. Bacteriol.* **192**, 3545–3546 (2010). 10.1128/JB.00366-10 PMC2897665 20418398

[31] Gaïa, M. et al. Mirusviruses link herpesviruses to giant viruses. *Nature* **616**, 783–789 (2023). 10.1038/s41586-023-05962-4 PMC10132985 37076623

[32] Dai, X. & Hong Zhou, Z. Structure of the herpes simplex virus 1 capsid with associated tegument protein complexes. *Science* **360**, eaao7298 (2018). 10.1126/science.aao7298 PMC5959011 29622628

[33] Heymann, J. B. et al. Dynamics of herpes simplex virus capsid maturation visualized by time-lapse cryo-electron microscopy. *Nat. Struct. Mol. Biol.* **10**, 334–341 (2003). 10.1038/nsb922 12704429

[34] Giessen, T. W. et al. Large protein organelles form a new iron sequestration system with high storage capacity. *Elife* **8**, e46070 (2019). 10.7554/eLife.46070 PMC6668986 31282860

[35] LaFrance, B. J. et al. The encapsulin from Thermotoga maritima is a flavoprotein with a symmetry matched ferritin-like cargo protein. *Sci. Rep.* **11**, 22810- (2021). 10.1038/s41598-021-01932-w PMC8610991 34815415

[36] Nichols, R. J. et al. Discovery and characterization of a novel family of prokaryotic nanocompartments involved in sulfur metabolism. *Elife* **10**, e59288 (2021). 10.7554/eLife.59288 PMC8049743 33821786

[37] Jones, J. A., Andreas, M. P. & Giessen, T. W. Structural basis for peroxidase encapsulation inside the encapsulin from the Gram-negative pathogen Klebsiella pneumoniae. *Nat. Commun.* **15**, 2558 (2024). 10.1038/s41467-024-46880-x PMC10960027 38519509

[38] Cardenas, J. P., Quatrini, R. & Holmes, D. S. Aerobic lineage of the oxidative stress response protein rubrerythrin emerged in an ancient microaerobic, (hyper)thermophilic environment. *Front. Microbiol.* **7**, 223475 (2016). 10.3389/fmicb.2016.01822 PMC5114695 27917155

[39] Kim, K. M. et al. Protein domain structure uncovers the origin of aerobic metabolism and the rise of planetary oxygen. *Structure* **20**, 67–76 (2012). 10.1016/j.str.2011.11.003 22244756

[40] Mateos, K. et al. The evolution and spread of sulfur cycling enzymes reflect the redox state of the early Earth. *Sci. Adv.* **9**, eade4847 (2023). 10.1126/sciadv.ade4847 PMC10328410 37418533

[41] Williams, R. J. P. Iron in evolution. *FEBS Lett.* **586**, 479–484 (2012). 10.1016/j.febslet.2011.05.068 21704034

[42] Andreas, M. P. & Giessen, T. W. Large-scale computational discovery and analysis of virus-derived microbial nanocompartments. *Nat. Commun.* **12**, 4748 (2021). 10.1038/s41467-021-25071-y PMC8346489 34362927

[43] Luque, A., Benler, S., Lee, D. Y., Brown, C. & White, S. The missing tailed Phages: prediction of small capsid candidates. *Microorganisms* **8**, 1944 (2020). 10.3390/microorganisms8121944 PMC7762592 33302408

[44] Chmelyuk, N. S., Oda, V. V., Gabashvili, A. N. & Abakumov, M. A. Encapsulins: structure, properties, and biotechnological applications. *Biochemistry* **88**, 35–49 (2023). 10.1134/S0006297923010042 PMC9937530 37068871

[45] Kashif-Khan, N., Savva, R. & Frank, S. Mining metagenomics data for novel bacterial nanocompartments. *NAR Genom. Bioinform.* **6**, lqae025 (2024).

[46] Gómez-Barrera, S. N. et al. Surface engineering of the encapsulin nanocompartment of *Myxococcus xanthus* for cell-targeted protein delivery. *ACS Omega* **10**, 7142–7152 (2025). 10.1021/acsomega.4c10285 PMC11866011 40028083

[47] Caspar, D. L. D. & Klug, A. Physical principles in the construction of regular viruses. *Cold Spring Harb. Symp. Quant. Biol.* **27**, 1–24 (1962). 10.1101/sqb.1962.027.001.005 14019094

[48] Twarock, R. & Luque, A. Structural puzzles in virology solved with an overarching icosahedral design principle. *Nat. Commun.* **10**, 4414 (2019). 10.1038/s41467-019-12367-3 PMC6765026 31562316

[49] Duda, R. L., Martincic, K., Xie, Z. & Hendrix, R. W. Bacteriophage HK97 head assembly. *FEMS Microbiol. Rev.* **17**, 41–46 (1995). 10.1111/j.1574-6976.1995.tb00186.x 7669350

[50] Fokine, A. & Rossmann, M. G. Molecular architecture of tailed double-stranded DNA phages. *Bacteriophage* **4**, e28281 (2014). 10.4161/bact.28281 PMC3940491 24616838

[51] Hardy, J. M. et al. The architecture and stabilisation of flagellotropic tailed bacteriophages. *Nat. Commun.* **11**, 3748 (2020). 10.1038/s41467-020-17505-w PMC7385642 32719311

[52] Morais, M. C. et al. Bacteriophage φ29 scaffolding protein gp7 before and after prohead assembly. *Nat. Struct. Mol. Biol.* **10**, 572–576 (2003). 10.1038/nsb939 12778115

[53] Podgorski, J. M. et al. Stabilization mechanism accommodating genome length variation in evolutionarily related viral capsids. *Nat. Commun.* **16**, 3145 (2025). *2025 16:1*. 10.1038/s41467-025-58298-0 PMC11965321 40175362

[54] Chen, D. H. et al. Structural basis for scaffolding-mediated assembly and maturation of a dsDNA virus. *Proc. Natl. Acad. Sci. USA* **108**, 1355–1360 (2011). 10.1073/pnas.1015739108 PMC3029737 21220301

[55] Ibarra, B. et al. Topology of the components of the DNA packaging machinery in the phage φ29 prohead. *J. Mol. Biol.* **298**, 807–815 (2000). 10.1006/jmbi.2000.3712 10801350

[56] Ignatiou, A. et al. Structural transitions during the scaffolding-driven assembly of a viral capsid. *Nat. Commun.* **10**, 1–11 (2019). 10.1038/s41467-019-12790-6 PMC6813328 31649265

[57] Woodson, M. et al. A viral genome packaging motor transitions between cyclic and helical symmetry to translocate dsDNA. *Sci. Adv.* **7**, eabc1955 (2021). 10.1126/sciadv.abc1955 PMC8104870 33962953

[58] Ionel, A. et al. Molecular rearrangements involved in the capsid shell maturation of bacteriophage T7. *J. Biol. Chem.* **286**, 234–242 (2011). 10.1074/jbc.M110.187211 PMC3012979 20962334

[59] Oh, B., Moyer, C. L., Hendrix, R. W. & Duda, R. L. The delta domain of the HK97 major capsid protein is essential for assembly. *Virology* **456–457**, 171–178 (2014). 10.1016/j.virol.2014.03.022 PMC4044616 24889236

[60] Roos, W. H. et al. Mechanics of bacteriophage maturation. *Proc. Natl. Acad. Sci. USA* **109**, 2342–2347 (2012). 10.1073/pnas.1109590109 PMC3289345 22308333

[61] Veesler, D. & Johnson, J. E. Virus maturation. *Annu. Rev. Biophys.* **41**, 473–496 (2012). 10.1146/annurev-biophys-042910-155407 PMC3607295 22404678

[62] Aznar, M., Luque, A. & Reguera, D. Relevance of capsid structure in the buckling and maturation of spherical viruses. *Phys. Biol.* **9**, 036003 (2012). 10.1088/1478-3975/9/3/036003 22555262

[63] Hawkins, D. E. D. P. et al. Insights into a viral motor: the structure of the HK97 packaging termination assembly. *Nucleic Acids Res*. **51**, 7025–7035 (2023). 10.1093/nar/gkad480 PMC10359639 37293963

[64] Rao, V. B., Fokine, A. & Fang, Q. The remarkable viral portal vertex: structure and a plausible model for mechanism. *Curr. Opin. Viro*l. **52**, 65–73 (2021). 10.1016/j.coviro.2021.09.004 PMC9579958 34619513

[65] Xu, J., Wang, D., Gui, M. & Xiang, Y. Structural assembly of the tailed bacteriophage ϕ29. *Nat. Commun.* **10**, 2366 (2019). 10.1038/s41467-019-10272-3 PMC6542822 31147544

[66] Berg, M. & Roux, S. Extreme dimensions — how big (or small) can tailed phages be?. *Nat. Rev. Microbiol.* **19**, 407–407 (2021). 2021 19:7. 10.1038/s41579-021-00574-z 33981030

[67] Helgstrand, C. et al. The Refined Structure of a Protein Catenane: The HK97 Bacteriophage Capsid at 3.44 Å Resolution. *J. Mol. Biol.* **334**, 885–899 (2003). 10.1016/j.jmb.2003.09.035 14643655

[68] Lander, G. C. et al. DNA bending-induced phase transition of encapsidated genome in phage λ. *Nucleic Acids Res.* **41**, 4518–4524 (2013). 10.1093/nar/gkt137 PMC3632124 23449219

[69] Yap, M. L. & Rossmann, M. G. Structure and function of bacteriophage T4. *Future Microbiol.* **9**, 1319–1337 (2014). 10.2217/fmb.14.91 PMC4275845 25517898

[70] González, B. et al. Phage G structure at 6.1 Å resolution, condensed DNA, and host identity revision to a lysinibacillus. *J. Mol. Biol.* **432**, 4139–4153 (2020). 10.1016/j.jmb.2020.05.016 PMC7316594 32454153

[71] Morais, M. C. et al. Conservation of the capsid structure in tailed dsDNA bacteriophages: the pseudoatomic structure of ϕ29. *Mol. Cell* **18**, 149–159 (2005). 10.1016/j.molcel.2005.03.013 15837419

[72] Choi, K. H., Morais, M. C., Anderson, D. L. & Rossmann, M. G. Determinants of bacteriophage ϕ29 head morphology. *Structure* **14**, 1723–1727 (2006). 10.1016/j.str.2006.09.007 17098197

[73] Aksyuk, A. A. et al. Structural investigations of a *Podoviridae streptococcus* phage C1, implications for the mechanism of viral entry. *Proc. Natl. Acad. Sci. USA* **109**, 14001–14006 (2012). 10.1073/pnas.1207730109 PMC3435189 22891295

[74] Kizziah, J. L., Rodenburg, C. M. & Dokland, T. Structure of the capsid size-determining scaffold of “Satellite” bacteriophage P4. *Viruses* **12**, 953 (2020). 10.3390/v12090953 PMC7552001 32867300

[75] Olsen, N. S., Hendriksen, N. B., Hansen, L. H. & Kot, W. A new high-throughput screening method for phages: enabling crude isolation and fast identification of diverse phages with therapeutic potential. *PHAGE* **1**, 137–148 (2020). 10.1089/phage.2020.0016 PMC9041460 36147828

[76] Haggård-Liungquist, E. et al. Bacteriophage P2:genes involved in baseplate assembly. *Virology* **213**, 109–121 (1995). 10.1006/viro.1995.1551 7483254

[77] Paez-Espino, D. et al. Uncovering Earth’s virome. *Nature* **536**, 425–430 (2016). 10.1038/nature19094 27533034

[78] Wigington, C. H. et al. Re-examination of the relationship between marine virus and microbial cell abundances. *Nat. Microbiol.* **1**, 15024 (2016). 10.1038/nmicrobiol.2015.24 27572161

[79] Wommack, K. E. & Colwell, R. R. Virioplankton: viruses in aquatic ecosystems. *Microbiol. Mol. Biol. Rev.* **64**, 69–114 (2000). 10.1128/mmbr.64.1.69-114.2000 PMC98987 10704475

[80] Cui, J., Schlub, T. E. & Holmes, E. C. An allometric relationship between the genome length and virion volume of viruses. *J. Virol.* **88**, 6403–6410 (2014). 10.1128/JVI.00362-14 PMC4093846 24672040

[81] Benler, S. et al. Thousands of previously unknown phages discovered in whole-community human gut metagenomes. *Microbiome* **9**, 78 (2021). 10.1186/s40168-021-01017-w PMC8008677 33781338

[82] Casjens, S. R. & Gilcrease, E. B. Determining DNA packaging strategy by analysis of the termini of the chromosomes in tailed bacteriophage virions. *Methods Mol. Biol.* **502**, 91–111 (2009). 10.1007/978-1-60327-565-1_7 PMC3082370 19082553

[83] Nayfach, S. et al. CheckV assesses the quality and completeness of metagenome-assembled viral genomes. *Nat. Biotechnol.* **39**, 578–585 (2021). 10.1038/s41587-020-00774-7 PMC8116208 33349699

[84] Finn, R. D. et al. Pfam: clans, web tools and services. *Nucleic Acids Res.* **34**, D247–D251 (2006). 10.1093/nar/gkj149 PMC1347511 16381856

[85] Medvedeva, S. et al. Three families of Asgard archaeal viruses identified in metagenome-assembled genomes. *Nat. Microbiol.* **7**, 962–973 (2022). 10.1038/s41564-022-01144-6 PMC11165672 35760839

[86] Moi, D. et al. Structural phylogenetics unravels the evolutionary diversification of communication systems in gram-positive bacteria and their viruses. *Nat. Struct. Mol. Biol.* **32**, 2492–2502 (2025). *2025 32:12*. 10.1038/s41594-025-01649-8 PMC12700828 41073779

[87] Flamholz, Z. N., Biller, S. J. & Kelly, L. Large language models improve annotation of prokaryotic viral proteins. *Nat. Microbiol.* **9**, 537–549 (2024). 10.1038/s41564-023-01584-8 PMC11311208 38287147

[88] Hawkins, N. C., Kizziah, J. L., Penadés, J. R. & Dokland, T. Shape shifter: redirection of prolate phage capsid assembly by staphylococcal pathogenicity islands. *Nat. Commun.* **12**, 6408 (2021). 10.1038/s41467-021-26759-x PMC8569155 34737316

[89] Rice, P., Longden, L. & Bleasby, A. EMBOSS: The European molecular biology open software suite. *Trends Genet.* **16**, 276–277 (2000). 10.1016/s0168-9525(00)02024-2 10827456

[90] Krupovic, M., Dolja, V. V. & Koonin, E. V. Origin of viruses: primordial replicators recruiting capsids from hosts. *Nat. Rev. Microbiol.* **17**, 449–458 (2019). 10.1038/s41579-019-0205-6 31142823

[91] Butkovic, A., Dolja, V. V., Koonin, E. V. & Krupovic, M. Plant virus movement proteins originated from jelly-roll capsid proteins. *PLoS Biol.* **21**, e3002157 (2023). 10.1371/journal.pbio.3002157 PMC10306228 37319262

[92] Huang, S., Zhang, Y., Chen, F. & Jiao, N. Complete genome sequence of a marine roseophage provides evidence into the evolution of gene transfer agents in alphaproteobacteria. *Virol. J.* **8**, 124- (2011). 10.1186/1743-422X-8-124 PMC3070671 21414219

[93] Kazlauskas, D., Varsani, A., Koonin, E. V. & Krupovic, M. Multiple origins of prokaryotic and eukaryotic single-stranded DNA viruses from bacterial and archaeal plasmids. *Nat. Commun.* **10**, 3425 (2019). 10.1038/s41467-019-11433-0 PMC6668415 31366885

[94] Krupovic, M., Makarova, K. S. & Koonin, E. V. Cellular homologs of the double jelly-roll major capsid proteins clarify the origins of an ancient virus kingdom. *Proc. Natl. Acad. Sci. USA* **119**, e2120620119 (2022). 10.1073/pnas.2120620119 PMC8812541 35078938

[95] Meier-Kolthoff, J. P., Uchiyama, J., Yahara, H., Paez-Espino, D. & Yahara, K. Investigation of recombination-intense viral groups and their genes in the Earth’s virome. *Sci. Rep.* **8**, 11496- (2018). 10.1038/s41598-018-29272-2 PMC6068154 30065291

[96] Howard-Varona, C., Hargreaves, K. R., Abedon, S. T. & Sullivan, M. B. Lysogeny in nature: mechanisms, impact and ecology of temperate phages. *ISME J*. 1–10 (2017). 10.1038/ismej.2017.16 PMC5520141 28291233

[97] Luque, A. & Silveira, C. B. Quantification of lysogeny caused by phage coinfections in microbial communities from biophysical principles. *mSystems* **5**, e00353–20 (2020). 10.1128/mSystems.00353-20 PMC7498681 32934113

[98] Giessen, T. W. & Silver, P. A. Widespread distribution of encapsulin nanocompartments reveals functional diversity. *Nat. Microbiol.* **2**, 17029 (2017). 10.1038/nmicrobiol.2017.29 28263314

[99] Hryc, C. F. et al. Accurate model annotation of a near-atomic resolution cryo-EM map. *Proc. Natl. Acad. Sci. USA* **114**, 3103–3108 (2017). 10.1073/pnas.1621152114 PMC5373346 28270620

[100] Selivanovitch, E., LaFrance, B. & Douglas, T. Molecular exclusion limits for diffusion across a porous capsid. *Nat. Commun.* **12**, 2903 (2021). 10.1038/s41467-021-23200-1 PMC8131759 34006828

[101] Wu, W. et al. Internal proteins of the procapsid and mature capsids of herpes simplex virus 1 mapped by bubblegram imaging. *J. Virol.* **90**, 5176–5186 (2016). 10.1128/JVI.03224-15 PMC4859710 26984725

[102] Ross, J. et al. Pore dynamics and asymmetric cargo loading in an encapsulin nanocompartment. *Sci. Adv.* **8**, 4461 (2022). 10.1126/sciadv.abj4461 PMC8791618 35080974

[103] Williams, E. M., Jung, S. M., Coffman, J. L. & Lutz, S. Pore engineering for enhanced mass transport in encapsulin nanocompartments. *ACS Synth. Biol.* **7**, 2514–2517 (2018). 10.1021/acssynbio.8b00295 30376298

[104] Wang, Y. & Douglas, T. Bioinspired approaches to self-assembly of virus-like particles: from molecules to materials. *Acc. Chem. Res.* **55**, 1349–1359 (2022). 10.1021/acs.accounts.2c00056 35507643

[105] Caetano-Anollés, G. Are viruses taxonomic units? a protein domain and loop-centric phylogenomic assessment. *Viruses* **16**, 1061 (2024). 10.3390/v16071061 PMC11281659 39066224

[106] Laurenceau, R., Raho, N., Forget, M., Arellano, A. A. & Chisholm, S. W. Frequency of mispackaging of Prochlorococcus DNA by cyanophage. * ISME J.* **15**, 129–140 (2020). 10.1038/s41396-020-00766-0 PMC7852597 32929209

[107] Subramanian, S., Kerns, H. R., Braverman, S. G. & Doore, S. M. The structure of Shigella virus Sf14 reveals the presence of two decoration proteins and two long tail fibers. *Commun. Biol.* **8**, 1–11 (2025). 10.1038/s42003-025-07668-x PMC11821841 39939755

[108] Dedeo, C. L., Cingolani, G. & Teschke, C. M. Portal protein: the orchestrator of capsid assembly for the dsDNA tailed bacteriophages and herpesviruses. *Annu. Rev. Virol.* **6**, 141–160 (2019). 10.1146/annurev-virology-092818-015819 PMC6947915 31337287

[109] de la Higuera, I. et al. Unveiling crucivirus diversity by mining metagenomic data. *mBio* **11**, 1–17 (2020). 10.1128/mBio.01410-20 PMC7468197 32873755

[110] Ravin, N. V. Replication and maintenance of linear phage-plasmid N15. *Microbiol. Spectr.* **3**, PLAS-0032-2014 (2015). 10.1128/microbiolspec.PLAS-0032-2014 26104561

[111] Mankertz, A. & Hillenbrand, B. Replication of porcine circovirus type 1 requires two proteins encoded by the viral rep gene. *Virology* **279**, 429–438 (2001). 10.1006/viro.2000.0730 11162799

[112] Sonntag, F. et al. The assembly-activating protein promotes capsid assembly of different adeno-associated virus serotypes. *J. Virol.* **85**, 12686–12697 (2011). 10.1128/JVI.05359-11 PMC3209379 21917944

[113] San Martín, C. & Van Raaij, M. J. The so far farthest reaches of the double jelly roll capsid protein fold 06 Biological Sciences 0601 Biochemistry and Cell Biology. *Virol. J.* **15**, 1–6 (2018). 10.1186/s12985-018-1097-1 PMC6260650 30470230

[114] Kejzar, N. et al. Cryo-EM structure of ssDNA bacteriophage ΦCjT23 provides insight into early virus evolution. *Nat. Commun.* **13**, 1–13 (2022). *2022 13:1*. 10.1038/s41467-022-35123-6 PMC9719478 36463224

[115] Laanto, E. et al. Virus found in a boreal lake links ssDNA and dsDNA viruses. *Proc. Natl. Acad. Sci. USA* **114**, 8378–8383 (2017). 10.1073/pnas.1703834114 PMC5547622 28716906

[116] Krupovic, M., Kuhn, J. H., Fischer, M. G. & Koonin, E. V. Natural history of eukaryotic DNA viruses with double jelly-roll major capsid proteins. *Proc. Natl. Acad. Sci. USA* **121**, e2405771121 (2024). 10.1073/pnas.2405771121 PMC11161782 38805295

[117] Diemer, G. S. & Stedman, K. M. A novel virus genome discovered in an extreme environment suggests recombination between unrelated groups of RNA and DNA viruses. *Biol. Direct* **7**, 13 (2012). 10.1186/1745-6150-7-13 PMC3372434 22515485

[118] Agarwal, M., Arthur, M., Arbeit, R. D. & Goldstein, R. Regulation of icosahedral virion capsid size by the in vivo activity of a cloned gene product. *Proc. Natl. Acad. Sci. USA* **87**, 2428–2432 (1990). 10.1073/pnas.87.7.2428 PMC53702 2181437

[119] deCarvalho, T. et al. Simultaneous entry as an adaptation to virulence in a novel satellite-helper system infecting Streptomyces species. *ISME J.* **17**, 2381–2388 (2023). 10.1038/s41396-023-01548-0 PMC10690885 37907733

[120] Penadés, J. R., Seed, K. D., Chen, J., Bikard, D. & Rocha, E. P. C. Genetics, ecology and evolution of phage satellites. *Nat. Rev. Microbiol.* **2025**, 1–13 (2025). 10.1038/s41579-025-01156-z 40148600

[121] Pourcel, C., Essoh, C., Ouldali, M. & Tavares, P. Acinetobacter baumannii satellite phage Aci01-2-Phanie depends on a helper myophage for its multiplication. *J. Virol.* **98**, e00667–24 (2024). 10.1128/jvi.00667-24 PMC11264900 38829140

[122] Kirchberger, P. C. & Ochman, H. Microviruses: A World Beyond phiX174. *Annu. Rev. Virol*. **10**, 99–118 (2023). 10.1146/annurev-virology-100120-011239 37774127

[123] Sun, L. et al. Icosahedral bacteriophage ΦX174 forms a tail for DNA transport during infection. *Nature* **505**, 432–435 (2014). 10.1038/nature12816 24336205

[124] Szyszka, T. N. et al. Point mutation in a virus-like capsid drives symmetry reduction to form tetrahedral cages. *Proc. Natl. Acad. Sci. USA* **121**, e2321260121 (2024). 10.1073/pnas.2321260121 PMC11098114 38722807

[125] Aznar, M. & Reguera, D. Physical ingredients controlling stability and structural selection of empty viral capsids. *J. Phys. Chem. B* **120**, 6147–6159 (2016). 10.1021/acs.jpcb.6b02150 27114062

[126] Luque, A., Reguera, D., Morozov, A., Rudnick, J. & Bruinsma, R. Physics of shell assembly: line tension, hole implosion, and closure catastrophe. *J. Chem. Phys.* **136**, 184507 (2012). 10.1063/1.4712304 22583300

[127] Zandi, R., Reguera, D., Bruinsma, R. F., Gelbart, W. M. & Rudnick, J. Origin of icosahedral symmetry in viruses. *Proc. Natl. Acad. Sci. USA* **101**, 15556–15560 (2004). 10.1073/pnas.0405844101 PMC524849 15486087

[128] Campillo-Balderas, J. A., Lazcano, A. & Becerra, A. Viral genome size distribution does not correlate with the antiquity of the host lineages. *Front. Ecol. Evol.* **3**, 159784 (2015).

[129] Díez-Villaseñor, C. & Rodriguez-Valera, F. CRISPR analysis suggests that small circular single-stranded DNA smacoviruses infect Archaea instead of humans. *Nat. Commun.* **10**, 294- (2019). 10.1038/s41467-018-08167-w PMC6336856 30655519

[130] Hagen, W. R. Quantum magnetism of the iron core in ferritin proteins—A re-evaluation of the giant-spin model. *Molecules* **29**, 2254 (2024). 10.3390/molecules29102254 PMC11123763 38792115

[131] Zhang, Y. & Orner, B. P. Self-Assembly in the Ferritin Nano-Cage Protein Superfamily. *Int. J. Mol. Sci.* **12**, 5406–5421 (2011). 10.3390/ijms12085406 PMC3179174 21954367

[132] He, D. et al. Structural characterization of encapsulated ferritin provides insight into iron storage in bacterial nanocompartments. *Elife* **5**, e18972 (2016). 10.7554/eLife.18972 PMC5012862 27529188

[133] Aziz, M. F. & Caetano-Anollés, G. Evolution of networks of protein domain organization. *Sci. Rep.* **11**, 12075- (2021). *2021 11:1*. 10.1038/s41598-021-90498-8 PMC8187734 34103558

[134] Toll-Riera, M. & Albà, M. M. Emergence of novel domains in proteins. *BMC Evol. Biol.* **13**, 47 (2013). 10.1186/1471-2148-13-47 PMC3599535 23425224

[135] Chen, I.-M. A. et al. IMG/M v.5.0: an integrated data management and comparative analysis system for microbial genomes and microbiomes. *Nucleic Acids Res.* **47**, D666–D677 (2019). 10.1093/nar/gky901 PMC6323987 30289528

[136] Mitchell, A. L. et al. MGnify: the microbiome analysis resource in 2020. *Nucleic Acids Res*. **48**, D570–D578(2019). 10.1093/nar/gkz1035 PMC7145632 31696235

[137] Nayfach, S., Shi, Z. J., Seshadri, R., Pollard, K. S. & Kyrpides, N. C. New insights from uncultivated genomes of the global human gut microbiome. *Nature* **568**, 505–510 (2019). 10.1038/s41586-019-1058-x PMC6784871 30867587

[138] Pasolli, E. et al. Extensive unexplored human microbiome diversity revealed by over 150,000 genomes from metagenomes spanning age, geography, and lifestyle. *Cell* **176**, 649–662 (2019). 10.1016/j.cell.2019.01.001 PMC6349461 30661755

[139] Soto-Perez, P. et al. CRISPR-Cas System of a prevalent human gut bacterium reveals Hyper-targeting against phages in a human virome catalog. *Cell Host Microbe* **26**, 325–335 (2019). 10.1016/j.chom.2019.08.008 PMC6936622 31492655

[140] Paez-Espino, D. et al. IMG/VR v.2.0: an integrated data management and analysis system for cultivated and environmental viral genomes. *Nucleic Acids Res.* **47**, D678–D686 (2019). 10.1093/nar/gky1127 PMC6323928 30407573

[141] Ivanova, N. et al. A call for standardized classification of metagenome projects. *Environ. Microbiol.* **12**, 1803–1805 (2010). 10.1111/j.1462-2920.2010.02270.x 20653767

[142] Hyatt, D. et al. Prodigal: prokaryotic gene recognition and translation initiation site identification. *BMC Bioinform.* **11**, 119 (2010). 10.1186/1471-2105-11-119 PMC2848648 20211023

[143] Luque, A., Zandi, R. & Reguera, D. Optimal architectures of elongated viruses. *PNAS* **107**, 1–6 (2010). 10.1073/pnas.0915122107 PMC2851771 20212146

[144] Luque, A. & Reguera, D. The Structure of Elongated Viral Capsids. *Biophys. J.* **98**, 2993–3003 (2010). 10.1016/j.bpj.2010.02.051 PMC2884239 20550912

[145] Terzian, P. et al. PHROG: families of prokaryotic virus proteins clustered using remote homology. *NAR Genom. Bioinform.* **3**, lqab067 (2021). 10.1093/nargab/lqab067 PMC8341000 34377978

[146] Kieft, K., Zhou, Z. & Anantharaman, K. VIBRANT: automated recovery, annotation and curation of microbial viruses, and evaluation of viral community function from genomic sequences. *Microbiome* **8**, 90 (2020). 10.1186/s40168-020-00867-0 PMC7288430 32522236

[147] Mistry, J. et al. Pfam: The protein families database in 2021. *Nucleic Acids Res.* **49**, D412–D419 (2021). 10.1093/nar/gkaa913 PMC7779014 33125078

[148] Zayed, A. A. et al. efam: an *e* xpanded, metaproteome-supported HMM profile database of viral protein *fam* ilies. *Bioinformatics* **37**, 4202–4208 (2021). 10.1093/bioinformatics/btab451 PMC9502166 34132786

[149] Söding, J. Protein homology detection by HMM–HMM comparison. *Bioinformatics* **21**, 951–960 (2005). 10.1093/bioinformatics/bti125 15531603

[150] Katoh, K. MAFFT: a novel method for rapid multiple sequence alignment based on fast Fourier transform. *Nucleic Acids Res.* **30**, 3059–3066 (2002). 10.1093/nar/gkf436 PMC135756 12136088

[151] Katoh, K. & Toh, H. Recent developments in the MAFFT multiple sequence alignment program. *Brief. Bioinform.* **9**, 286–298 (2008). 10.1093/bib/bbn013 18372315

[152] Minh, B. Q. et al. IQ-TREE 2: New models and efficient methods for phylogenetic inference in the genomic Era. *Mol. Biol. Evol.* **37**, 1530–1534 (2020). 10.1093/molbev/msaa015 PMC7182206 32011700

[153] Letunic, I. & Bork, P. Interactive tree of life (iTOL) v6: recent updates to the phylogenetic tree display and annotation tool. *Nucleic Acids Res.* **52**, W78–W82 (2024). 10.1093/nar/gkae268 PMC11223838 38613393

[154] Balaban, M., Moshiri, N., Mai, U., Jia, X. & Mirarab, S. TreeCluster: Clustering biological sequences using phylogenetic trees. *PLoS ONE* **14**, e0221068 (2019). 10.1371/journal.pone.0221068 PMC6705769 31437182

[155] Brister, J. R., Ako-adjei, D., Bao, Y. & Blinkova, O. NCBI Viral Genomes Resource. *Nucleic Acids Res.* **43**, D571–D577 (2015). 10.1093/nar/gku1207 PMC4383986 25428358

[156] Alqurainy, N. et al. A widespread family of phage-inducible chromosomal islands only steals bacteriophage tails to spread in nature. *Cell Host Microbe* **31**, 69–82 (2023). 10.1016/j.chom.2022.12.001 36596306

[157] Fillol-Salom, A. et al. Phage-inducible chromosomal islands are ubiquitous within the bacterial universe. *ISME J.* **12**, 2114–2128 (2018). 10.1038/s41396-018-0156-3 PMC6092414 29875435

[158] Penadés, J. R. & Christie, G. E. The Phage-Inducible Chromosomal Islands: A Family of Highly Evolved Molecular Parasites. *Annu. Rev. Virol.* **2**, 181–201 (2015). 10.1146/annurev-virology-031413-085446 26958912

[159] Meng, E. C. et al. UCSF ChimeraX: Tools for structure building and analysis. *Protein Sci.* **32**, e4792 (2023). 10.1002/pro.4792 PMC10588335 37774136

[160] Podgorski, J. M. et al. A structural dendrogram of the actinobacteriophage major capsid proteins provides important structural insights into the evolution of capsid stability. *Structure* **31**, 282–294.e5 (2023). 10.1016/j.str.2022.12.012 PMC10071307 36649709

[161] Stone, N. P., Demo, G., Agnello, E. & Kelch, B. A. Principles for enhancing virus capsid capacity and stability from a thermophilic virus capsid structure. *Nat. Commun.* **10**, 4471 (2019). 10.1038/s41467-019-12341-z PMC6775164 31578335

[162] Hagberg, A. A., Schult, D. A. & Swart, P. J. Exploring network structure, dynamics, and Function using NetworkX. In* Proceedings of the** Python in Science Conference* 11–15 (SciPy, 2008).

[163] Nasir, A. & Caetano-Anollés, G. A phylogenomic data-driven exploration of viral origins and evolution. *Sci. Adv.* **1**, e1500527 (2015). 10.1126/sciadv.1500527 PMC4643759 26601271

[164] Jumper, J. et al. Highly accurate protein structure prediction with AlphaFold. *Nature* **596**, 583–589 (2021). 10.1038/s41586-021-03819-2 PMC8371605 34265844

[165] Mutti, G., Ocaña-Pallarès, E. & Gabaldón, T. Newly Developed Structure-Based Methods Do Not Outperform Standard Sequence-Based Methods for Large-Scale Phylogenomics. *Mol. Biol. Evol.* **42**, msaf149 (2025). 10.1093/molbev/msaf149 PMC12290511 40580945

[166] van Kempen, M. et al. Fast and accurate protein structure search with Foldseek. *Nat. Biotechnol.* **42**, 243–246 (2024). 10.1038/s41587-023-01773-0 PMC10869269 37156916

