# Supplementary material for: Small viruses reveal bidirectional evolution between HK97-fold viruses and encapsulins via procapsids
Source: Nat Commun. 2026 Oct 14;17:10571. doi: 10.1038/s41467-026-78136-1 (PMC13669383; doi:10.1038/s41467-026-78136-1)
Supplement: Supplementary file 1 [file 41467_2026_78136_MOESM1_ESM.pdf]

Supplementary materials

Small viruses reveal bidirectional evolution between HK97-fold viruses and encapsulins via procapsids

Abelardo Aguilar[1], Ingrid Miranda Perez[1,2], Omer Nadel[1], Stephen Nayfach[3], Luis Ruiz Pestana[4,5], Anca M. Segall[6,7], Forest L. Rohwer[6,7], Simon Roux[8], Simon White[9], and Antoni Luque[1,5,10,*]

[1]Department of Biology, University of Miami, Coral Gables, FL, USA.

[2]Department of Evolutionary Biology, Universidad Nacional Autónoma de México, Ciudad Universitaria, Mexico City, México.

[3]Profluent Bio, Emeryville, CA, USA.

[4]Department of Civil and Architectural Engineering, University of Miami, Coral Gables, FL, USA.

[5]Department of Chemical, Environmental, and Materials Engineering, University of Miami, Coral Gables, FL, USA.

[6]Department of Biology, San Diego State University, San Diego, CA, USA.

[7]Viral Information Institute, San Diego State University, San Diego, CA, USA.

[8]Department of Energy Joint Genome Institute, Lawrence Berkeley National Laboratory, Berkeley, CA, USA.

[9]Department of Molecular and Cell Biology, University of Connecticut, Storrs, CT, USA.

[10]Department of Physics, University of Miami, Coral Gables, FL, USA.

[*]Corresponding author: antoni.luque@miami.edu

This file contains all the supplementary materials for the manuscript titled "Small viruses reveal bidirectional evolution between HK97-fold viruses and encapsulins via procapsids" by Aguilar *et al*. Supplementary materials are divided into two sections: 1) Supplementary Figures, and 2) Links to data files.

SECTION 1. SUPPLEMENTARY FIGURES

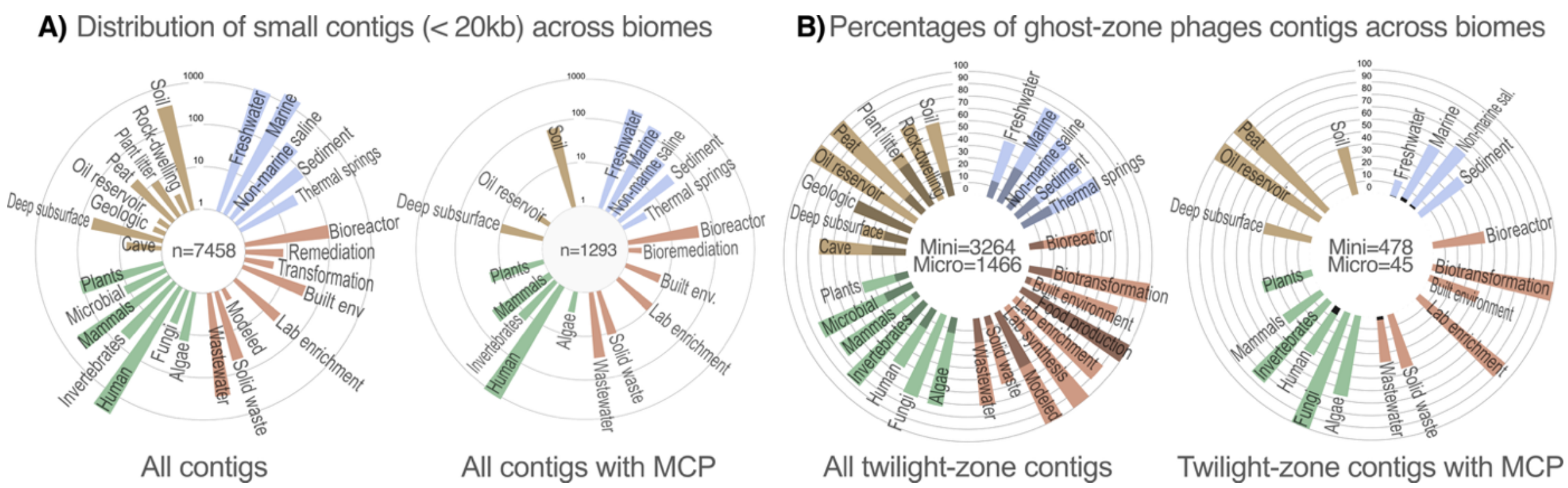

Figure S1. A) Circular bar plots show aggregate viral contig counts (log scale) by biome for n=7,458 viral contigs with level-2 biome annotations: all contigs (left) and MCP-containing contigs (right; n=1,293 viral contigs). B) Similar plot to A, but bars display aggregate percentages and focus on the Twilight Zone region (<20 kbp); darker bars indicate micro contigs (<5 kbp). Panel B includes n=3,264 mini contigs and n=1,466 micro contigs among all contigs, and n=478 mini contigs and n=45 micro contigs among MCP-containing contigs. Source data are provided as a Source Data file.

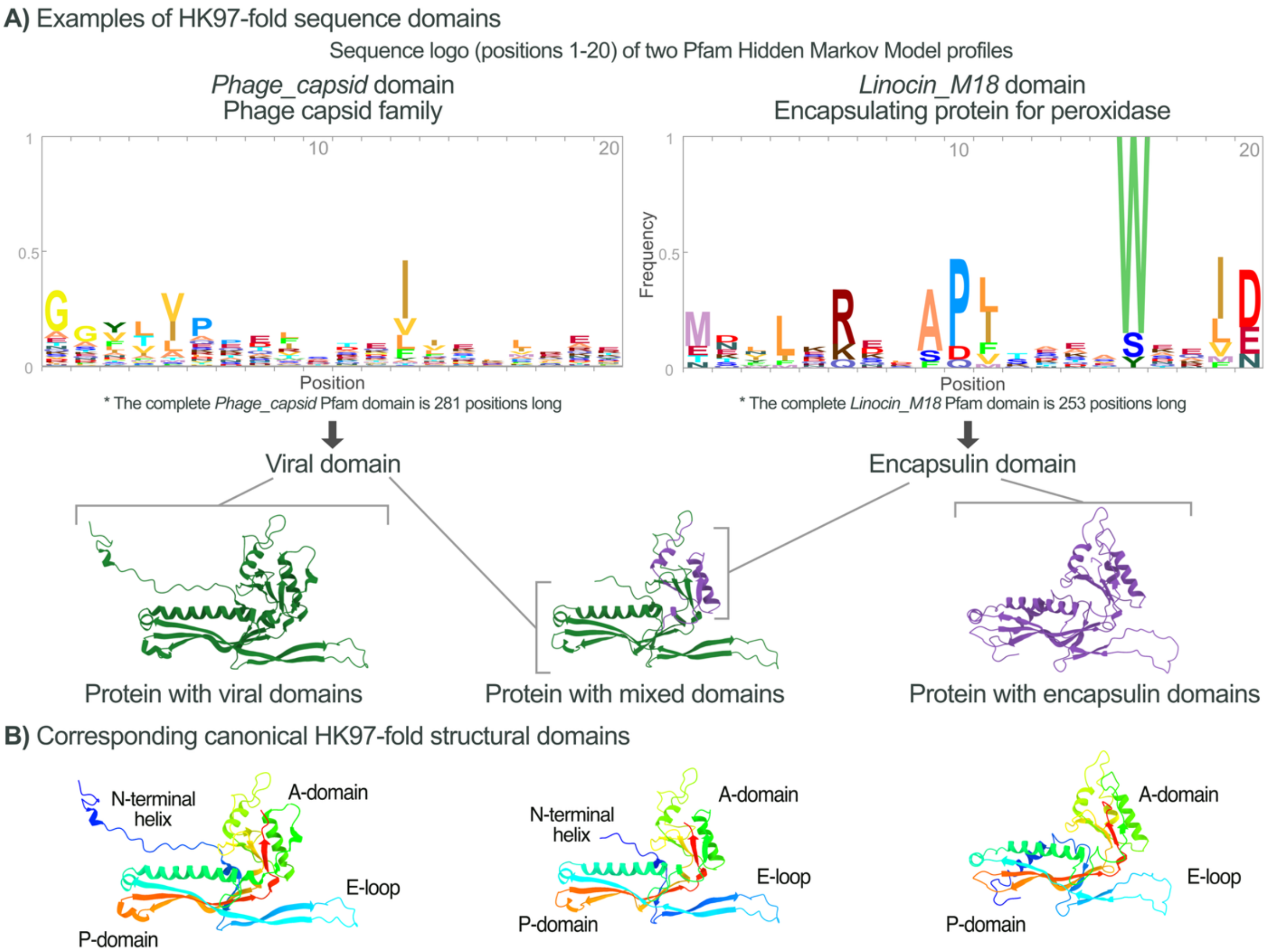

Figure S2. Sequence-bas*ed domains in HK97-fold s*tructural proteins are illustrated as follows: A) Two Pfam domain logos: *Phage_capsid*, linked to viruses, and *Linocin_M18*, linked to Family I encapsulins, with examples of predicted viral, encapsulin, and mixed domain structures. B) Canonical HK97-fold protein domains are shown as rainbow-colored structure predictions.

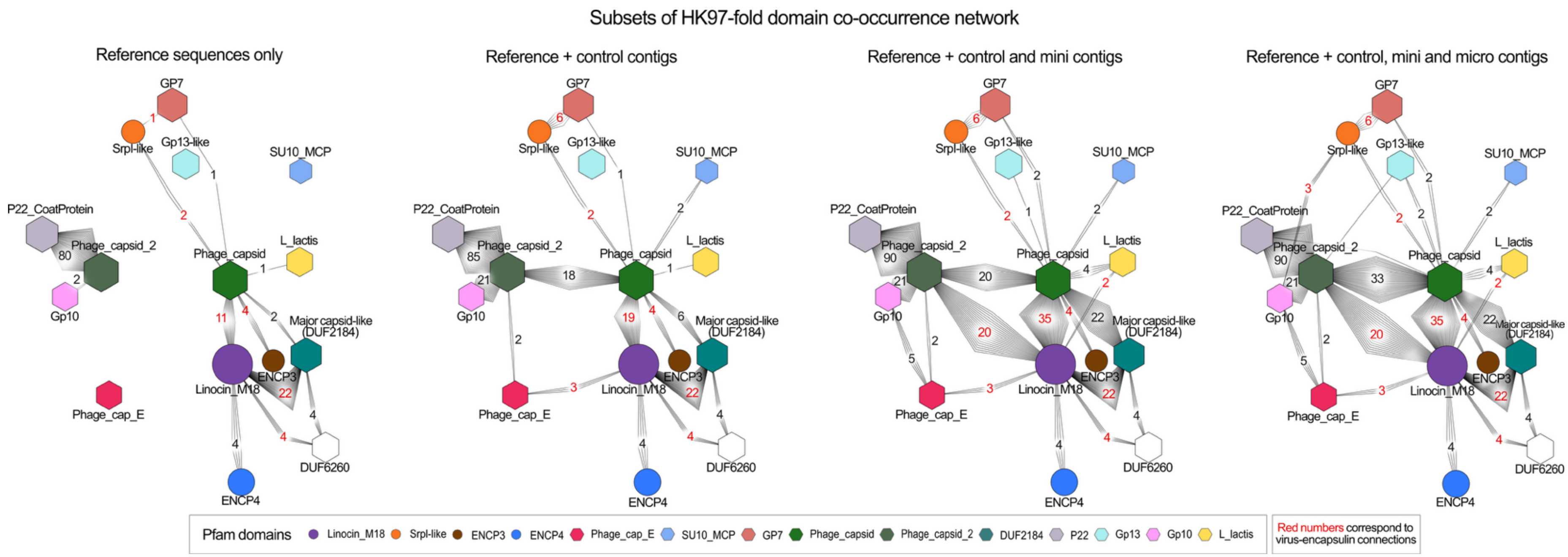

Figure S3. Domain co-occurrence networks across progressively expanded datasets. Networks summarize Pfam profiles that co-occur within a protein, shown for (left to right) reference sequences only, reference + control contigs, reference + control and mini contigs, and reference + control, mini, and micro contigs. Nodes represent individual Pfam domains (colors/shapes as in the Pfam legend), and node size scales with total degree. Edges connect domains observed in the same protein; labels indicate the number of proteins supporting each co-occurrence. Red edge labels highlight co-occurrences between typically viral and typically encapsulin-associated domains (also referred to as "non-canonical connections"). Source data are provided as a Source Data file.

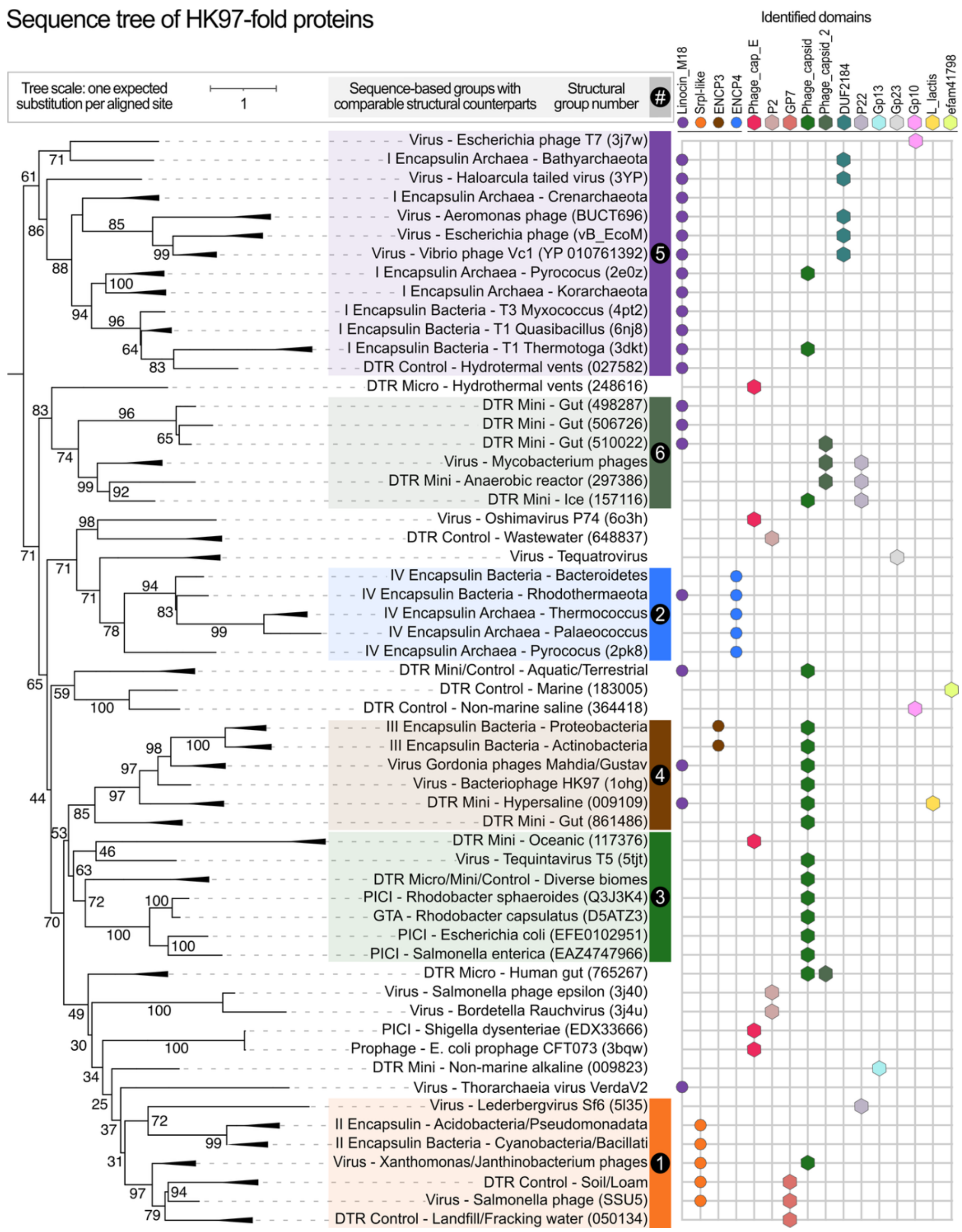

Figure S4. Maximum-likelihood phylogeny of HK97-fold protein sequences. Sequence-based groups comparable to those recovered in the structure-based tree are highlighted by colored boxes behind tip labels and by the adjacent colored strip indicating the corresponding group number in the structure-based tree. Here, comparable denotes groups that recover the same broad encapsulin–virus association across trees, although exact viral membership and internal within-group topology differ. The panel on the right summarizes Pfam domain presence for each protein: columns correspond to domains (symbols and labels at the top), and symbols within the grid indicate detected domains in each sequence. Symbol shape denotes domain type: circles represent domains typically associated with cellular encapsulins, whereas hexagons represent domains typically associated with viruses (as indicated in the domain header). The scale bar indicates the expected amino acid substitutions per aligned site. Source data are provided as a Source Data file.

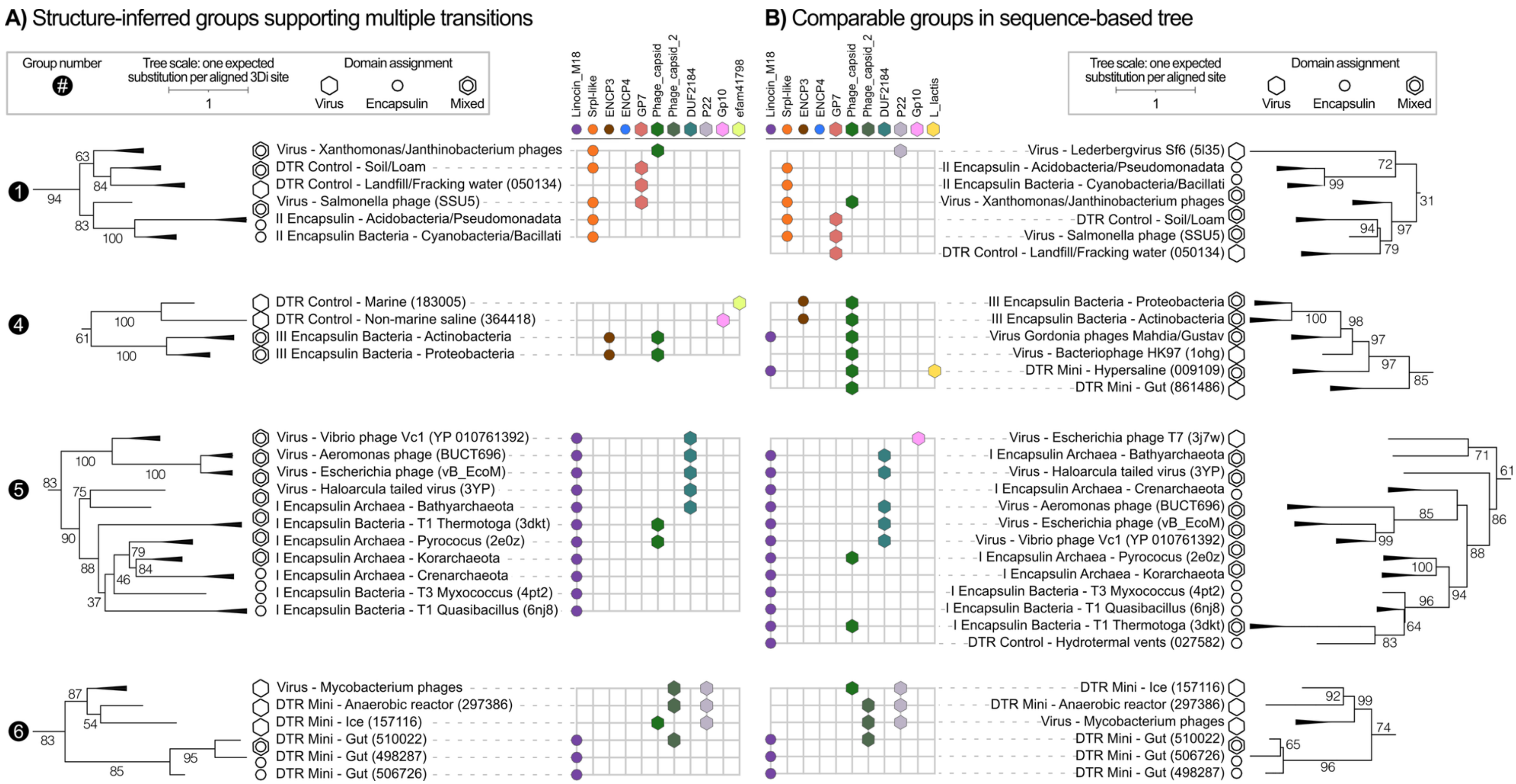

Figure S5. A) Selected groups from the structure-based tree containing viral and encapsulin domains. Tip symbols summarize the Pfam domain composition of each protein: hexagons denote proteins with viral-associated Pfam domains only, circles denote proteins with encapsulin-associated Pfam domains only, and combined symbols denote proteins with mixed domain composition. The Pfam domain presence/absence matrix shows selected domains detected in each protein. Branch lengths are reported as expected substitutions per aligned 3Di site, and node labels indicate bootstrap support values. B) Comparable groups in the sequence-based phylogeny, shown using the same tip set and domain annotations. Here, comparable refers to groups that recover the same broad virus–encapsulin association pattern, although the exact membership and internal topology differ. Branch lengths are reported as expected amino acid substitutions per aligned site, and node labels indicate bootstrap support values. Source data are provided as a Source Data file.

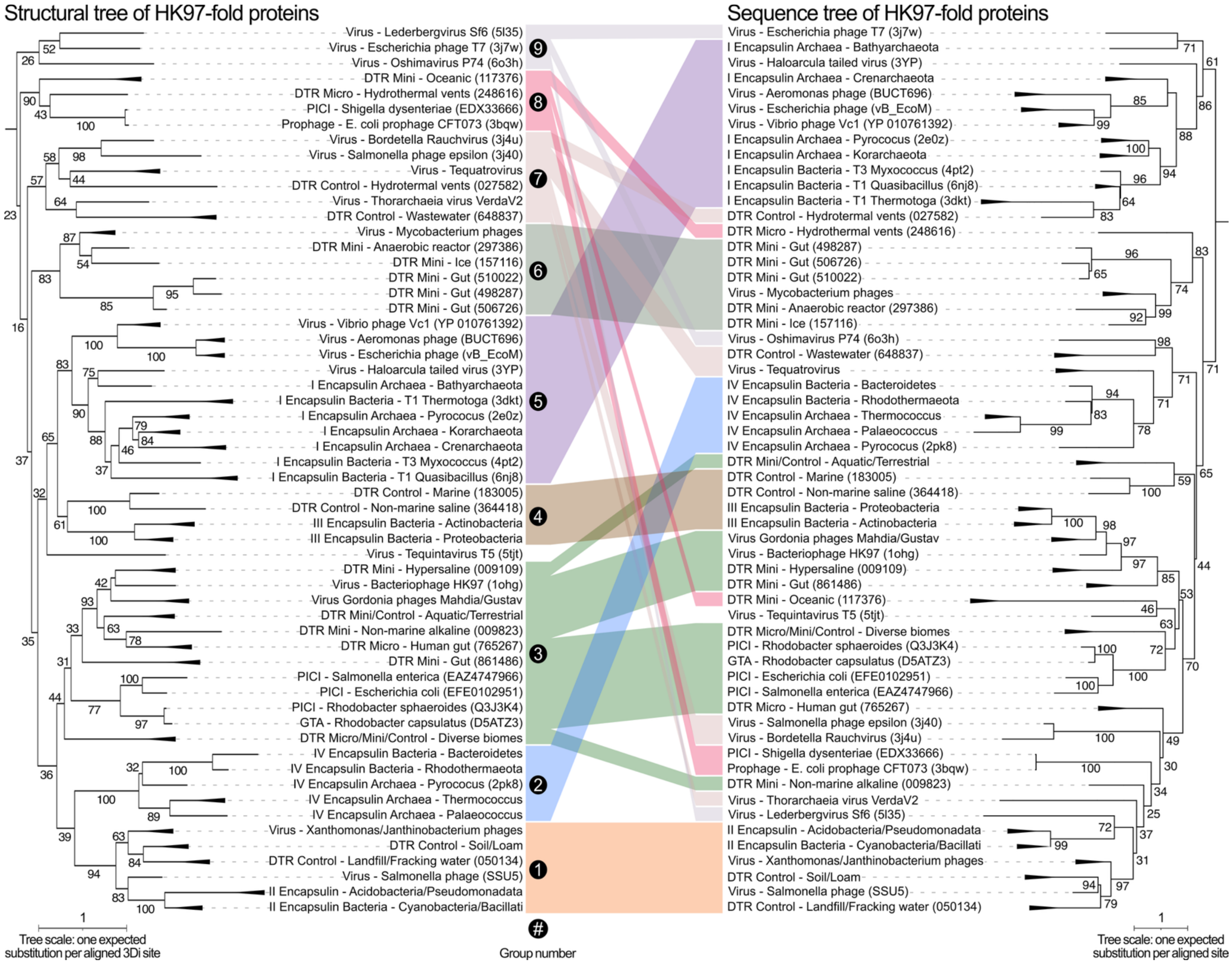

Figure S6. Global comparison of structure- and sequence-based HK97-fold trees. The structure-based tree (left) and the sequence-based tree (right) are shown. Tip labels are shared between trees, and adjacent numbers indicate the proposed structural groups. Colored bands trace the placement of groups from the structure-based tree across the sequence-based tree. Scale bars are shown for each tree. Source data are provided as a Source Data file.

Bacillus thermotolerans iron storage encapsulin | *Staphylococcus* SaPI

T=4 shells

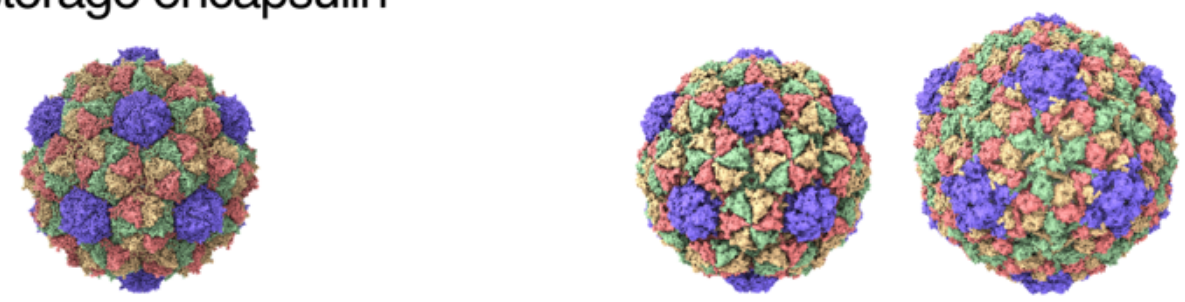

	Encapsulin	Procapsid	Mature capsid
Volume (nm^3)	2.12E+04	2.18E+04	3.50E+04
Projected capacity (kbp)	10.6	10.9	17.5
Genome length (kbp)	-	15.2	15.2

Byrnievirus HK97 | Enterobacteria phage λ

T=7 shells

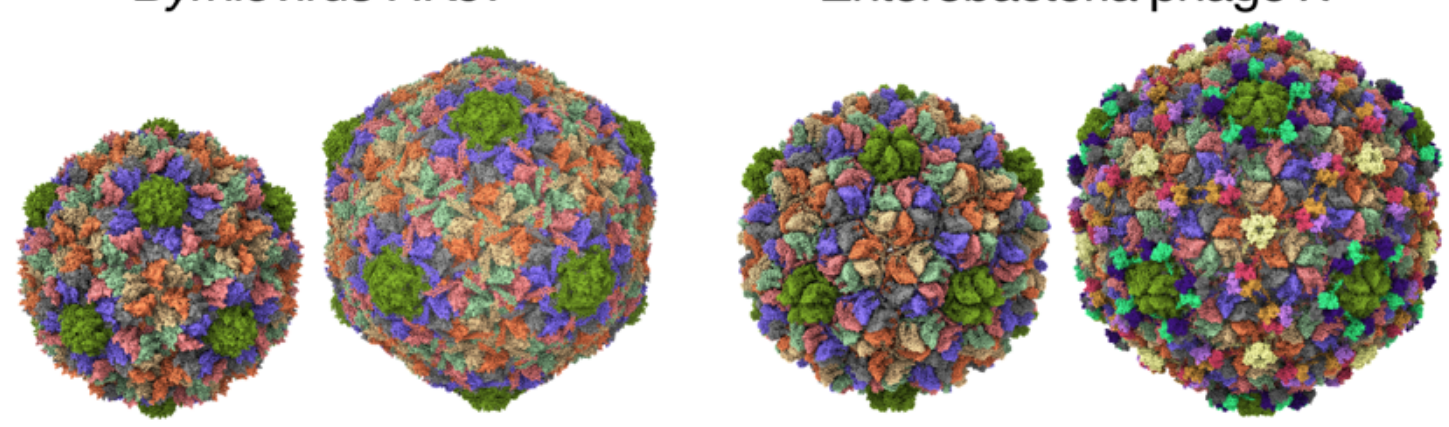

	Procapsid	Mature capsid	Procapsid	Mature capsid
Volume (nm^3)	3.94E+04	8.22E+04	4.78E+04	9.78E+04
Projected capacity (kbp)	19.7	41.1	23.9	48.9
Genome length (kbp)	39.7	39.7	48.5	48.5

Enterobacteria phage T4

T=13 prolate shells

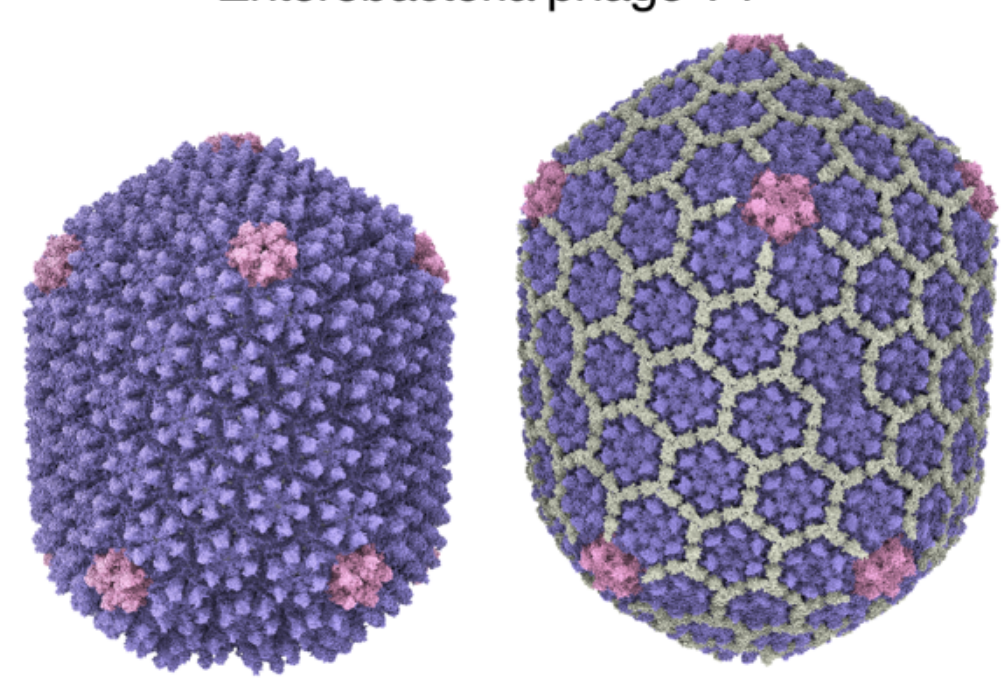

	Procapsid	Mature capsid
Volume (nm^3)	2.10E+05	3.50E+05
Projected capacity (kbp)	105	175
Genome length (kbp)	168.9	168.9

Figure S7. Structural comparison of encapsulin and viral capsid architectures across different triangulation numbers (T). The figure shows atomic reconstructions of an encapsulin (T=4; iron storage encapsulin PDB: 6NJ8), procapsid and mature capsid of Staphylococcus aureus Pathogenicity Island (SaPI, T=4; procapsid: PDB 7RWZ, mature capsid: PDB 8VD4), Enterobacteria phage HK97 (T=7; procapsid: PDB 3E8K, mature capsid: PDB 1OHG), Enterobacteria phage λ (T=7; procapsid: PDB 7VI9, mature capsid: PDB 7VII), and Enterobacteria phage T4 (T=13 prolate; procapsid: PDB 7VRT, mature capsid: PDB 7VS5).

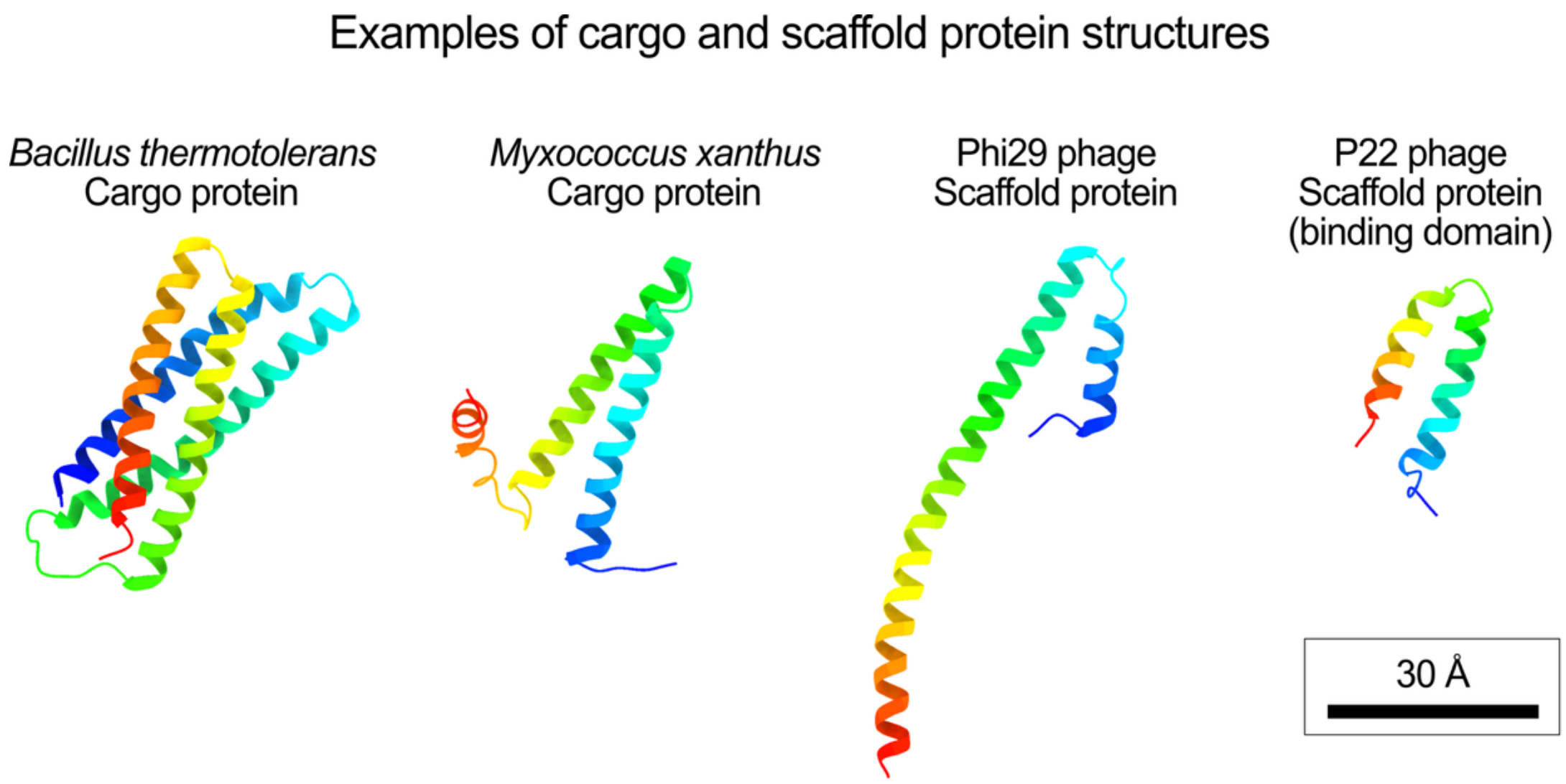

Figure S8. Structural comparison of encapsulin cargo and phage scaffold proteins. Cartoon representations of (left to right) the cargo proteins from *Bacillus thermotolerans* encapsulin (PDB 6N63) and *Myxococcus xanthus* encapsulin (PDB 7S8T), the scaffold protein from bacteriophage Φ29 (PDB 8F2M), and the capsid-binding domain of the P22 scaffold protein (PDB 2GP8). All proteins are shown at the same scale (scale bar, 30 Å). Each adopts an elongated alpha-helical hairpin "hinge-like" architecture.

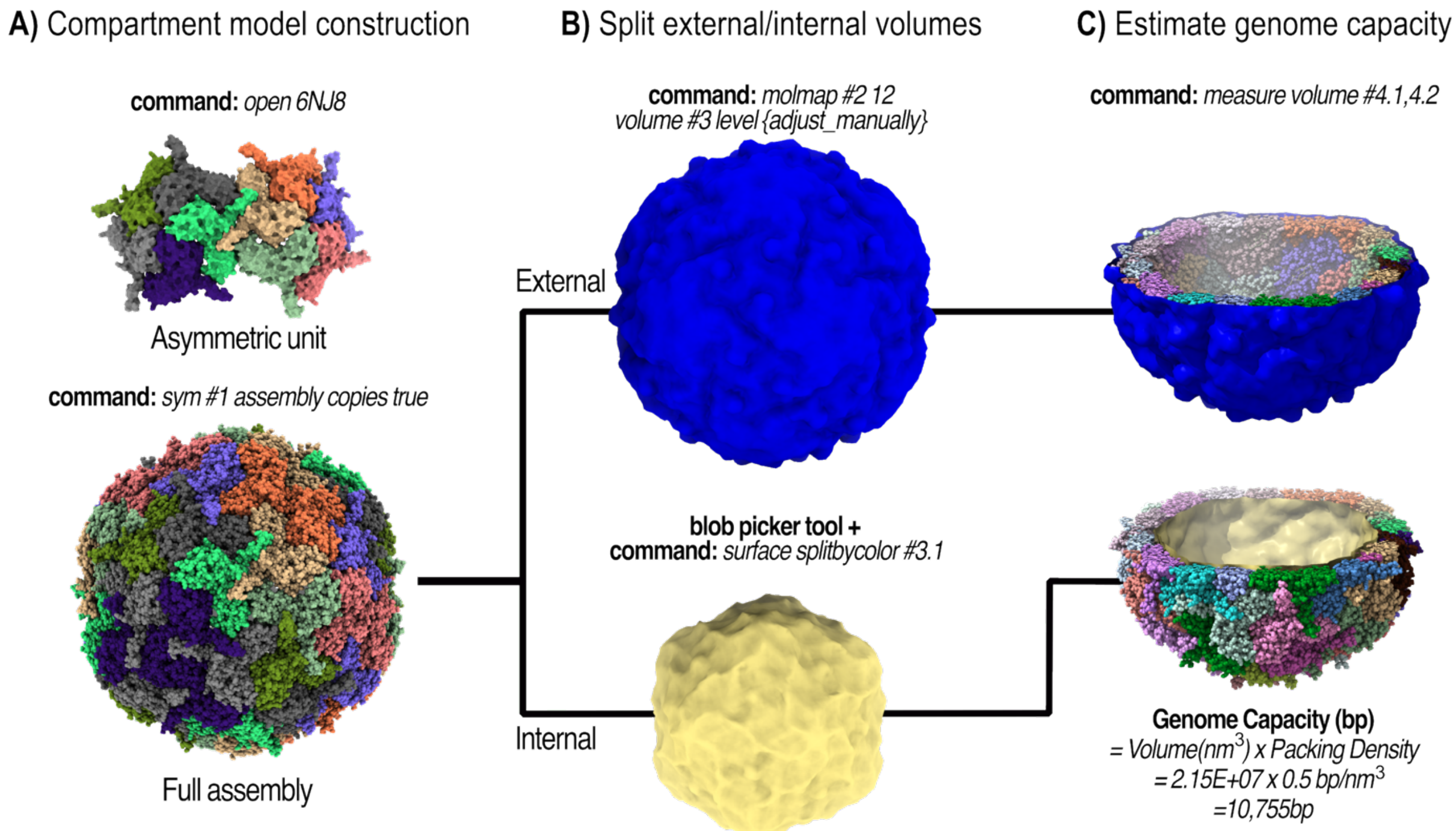

Figure S9. Workflow for measuring the enclosed volume of HK97-fold compartments using ChimeraX. A) ChimeraX's commands used to reconstruct the full capsid assembly from asymmetric unit coordinates, generating the complete surface model. B) Creation of a molecular density map from the surface model to split the external and internal surfaces and isolate the internal cavity for volume analysis. C) The enclosed internal volume is measured using '*measure volume'* command. This estimated volume is then used to calculate the potential genome capacity.

SECTION 2. LINKS TO FIGSHARE FILES

Environmental viral genomes – available for download at:
https://doi.org/10.6084/m9.figshare.30943028

`data_file_S1_IMG_datasets.xlsx`
Complete list of Joint Genome Institute, Integrated Microbial Genomes & Microbiomes associated datasets. This file contains the identifiers of each study, as well as its associated DOIs.

`data_file_S2_biomes.csv`
Hierarchical biome classification schema used to map metagenomic contigs to ecological categories, following Ivanova et al. (2010). Includes all possible biome categories at multiple levels.

`data_file_S3_associated_biomes.csv`
Metadata table links each environmental viral contig to its assigned biome(s), along with relevant sample-level information.

`data_file_S4_genomes.fna`
FASTA file containing all environmental viral contigs analyzed in this study. Each contig has direct terminal repeats (DTRs) and encodes at least one hallmark gene of Duplodnaviria phages.

Protein function annotation – available for download at:
https://doi.org/10.6084/m9.figshare.30948863

`data_file_S5_ORFs.gb`
Multi-GenBank file containing all contigs with their predicted open reading frames (ORFs), including annotations used for downstream protein-level analyses.

`data_file_S6_plm_annotation.zip`
Contains protein function predictions generated with the Virus Protein Function Protein Language Model (VPF-PLM). Includes per-protein `.csv` files with predicted functions and functional probability matrices, as well as `.pkl` embeddings.

`data_file_S7_vibrant_annotation.zip`
VIBRANT-derived annotations for environmental viral contigs. Includes KEGG, Pfam, VOG, and VPFAM HMM scan results, and summary figures describing genome size, quality, and gene content.

`data_file_S8_HK97_hmm_profiles.zip`
Hidden Markov Model (HMM) profiles used to detect HK97-fold proteins. Includes Pfam clan CL0373 (“Phage_coat”) profiles and extended sets from the efam database.

`data_file_S9_hhpred_annotation.zip`
Archive of `.hhr` output files from HHpred, used for remote homology and function inference. These were manually reviewed for key annotations.

HK97-fold proteins evolutionary analyses (sequences, structures, and phylogenies) – available for download at: https://doi.org/10.6084/m9.figshare.30948881

`data_file_S10_environmental_HK97_proteins.faa`
FASTA file of amino acid sequences for all HK97-fold major capsid proteins (MCPs) detected in environmental viral genomes.

`data_file_S11_environmental_MCPs_trees.zip`
IQ-TREE outputs for all environmental HK97 MCPs, including maximum-likelihood trees, model statistics, and TreeCluster groupings at varying distance thresholds.

`data_file_S12_HK97_reference_identifiers.txt`
List of protein and genome identifiers used in the reference HK97-fold dataset, including entries for encapsulins, PICIs, GTAs, and phages.

`data_file_S13_IDs_and_encapsulin_enrichment.csv`
List of HK97-fold protein sequence identifiers (environmental contigs, reference viruses and encapsulins), and phage coat and encapsulin coverage per protein.

`data_file_S14_HK97_protein_structures.zip`
Protein structure models used for structural phylogenetics. Includes both experimentally resolved structures (PDB) and AlphaFold-predicted models with confidence assessments.

`data_file_S15_HK97_large_scale_ml3di_structural_tree.txt`
Newick tree file of structure-based phylogenetic tree of HK97-fold proteins across viruses, encapsulins, PICIs, GTAs, and viral environmental contigs.

`data_file_S16_HK97_large_scale_sequence_tree.txt`
Newick tree file of sequence-based phylogenetic tree of HK97-fold proteins across viruses, encapsulins, PICIs, GTAs, and viral environmental contigs.

`data_file_S17_tail_proteins_non_encapsulin_enriched_genomes.zip`
Annotation summaries for tail-related proteins detected in DTR-containing twilight-zone genomes lacking encapsulin domains in their HK97 MCPs.

SECTION 3. SUPPLEMENTARY TABLES

Table S1. Prevalence of contigs across biomes. The table displays the counts for each biome and genome length group, indicating the number of contigs with MCPs. Source data are provided as a Source Data file.

Biome	n	Micro	Mini	Control	n with MCP	Micro With MCP	Mini with_MCP	Control with_MCP
Freshwater	1107	161	375	571	233	2	31	200
Marine	1596	505	873	218	156	5	75	76
Non-marine saline	182	25	105	52	47	1	28	18
Sediment	92	21	39	32	36	0	16	20
Thermal springs	33	9	14	10	4	0	0	4
Bioreactor	105	13	45	47	44	0	19	25
Bioremediation	8	0	0	8	2	0	0	2
Biotransformation	5	1	4	0	1	0	1	0
Built environment	38	2	28	8	7	0	3	4
Food production	1	1	0	0	0	0	0	0
Lab enrichment	23	0	21	2	10	0	9	1
Lab synthesis	1	0	1	0	0	0	0	0
Modeled	2	1	1	0	0	0	0	0
Solid waste	49	4	24	21	11	0	5	6
Wastewater	288	59	146	83	32	1	11	20
Algae	15	2	9	4	3	0	2	1
Fungi	7	0	5	2	1	0	1	0
Human	2867	515	1033	1319	562	36	212	314
Invertebrates	55	12	36	7	14	0	12	2
Mammals	413	24	291	98	24	0	9	15
Microbial	12	3	7	2	0	0	0	0
Plants	110	13	30	67	19	0	8	11
Cave	7	2	3	2	0	0	0	0
Deep subsurface	54	11	10	33	10	0	4	6
Geologic	2	1	0	1	1	0	0	1
Oil reservoir	2	0	2	0	2	0	2	0
Peat	21	4	17	0	1	0	1	0
Plant litter	10	4	1	5	0	0	0	0
Rock-dwelling (endoliths)	3	0	2	1	0	0	0	0
Soil	349	73	141	135	73	0	29	44
Volcanic	1	0	1	0	0	0	0	0

Table S2. Prevalence of tail genes. The table summarizes the counts of tail genes across the genome length groups (micro, mini, and control), distinguishing between those with and without encapsulin enrichment. Source data are provided as a Source Data file.

Genome length group	Encapsulin domains presence	n	Genomes with tails count	%with tail	n tails = 1	n tails >1	Max tail count	n tail proteins
**mini**	FALSE	398	14	3.52	14	0	1	14
**mini**	TRUE	93	1	1.08	1	0	1	1
**micro**	FALSE	45	8	17.78	5	3	4	13
**micro**	TRUE	0	0	0	0	0	0	0
**control**	FALSE	778	97	12.47	81	16	5	124
**control**	TRUE	20	0	0	0	0	0	0

Table S3. Pairwise statistical comparisons for phage-coat coverage (Figure 3B). Pairwise group comparisons for positive phage-coat coverage values, reporting group sample sizes, group means/medians, bootstrap 95% confidence intervals for mean and median differences, Cliff's delta (probability of superiority), and adjusted p-values from the primary post-hoc test (Games–Howell following Welch's one-way ANOVA). Source data are provided as a Source Data file.

group1	group2	n1	n2	mean 1	mean 2	median 1	median 2	cliffs_delta	delta_lo	delta_hi	prob_ superiority	p_value	p_stars
**Micro**	**Refseq**	45	910	56.7	84.0	59.8	89.9	-0.8	-0.9	-0.6	0.1	0	****
**Mini**	**Refseq**	491	910	60.4	84.0	62.6	89.9	-0.6	-0.7	-0.6	0.2	0	****
**Mini**	**ENC_1**	491	55	60.4	86.2	62.6	94.0	-0.7	-0.8	-0.6	0.2	0	****
**Control**	**Refseq**	798	910	57.1	84.0	59.7	89.9	-0.7	-0.7	-0.7	0.1	0	****
**Control**	**ENC_1**	798	55	57.1	86.2	59.7	94.0	-0.7	-0.8	-0.7	0.1	0	****
**Micro**	**ENC_1**	45	55	56.7	86.2	59.8	94.0	-0.8	-0.9	-0.6	0.1	3.43E-10	****
**Refseq**	**PICIs**	910	66	84.0	66.9	89.9	72.4	0.6	0.5	0.7	0.8	7.58E-10	****
**PICIs**	**ENC_1**	66	55	66.9	86.2	72.4	94.0	-0.7	-0.8	-0.5	0.2	2.42E-08	****
**Refseq**	**ENC_2**	910	31	84.0	69.9	89.9	77.2	0.6	0.5	0.7	0.8	2.09E-05	****
**ENC_1**	**ENC_2**	55	31	86.2	69.9	94.0	77.2	0.7	0.5	0.8	0.8	2.24E-05	****
**Control**	**ENC_2**	798	31	57.1	69.9	59.7	77.2	-0.4	-0.6	-0.3	0.3	0.000106	***
**Micro**	**ENC_4**	45	14	56.7	79.1	59.8	80.0	-0.7	-0.9	-0.4	0.2	0.00062	***
**Control**	**ENC_4**	798	14	57.1	79.1	59.7	80.0	-0.7	-0.8	-0.5	0.2	0.000723	***
**Control**	**PICIs**	798	66	57.1	66.9	59.7	72.4	-0.3	-0.5	-0.2	0.3	0.001	**
**Micro**	**Fringe**	45	11	56.7	85.7	59.8	92.9	-0.7	-1.0	-0.3	0.1	0.003	**
**Mini**	**ENC_4**	491	14	60.4	79.1	62.6	80.0	-0.6	-0.8	-0.4	0.2	0.003	**
**Micro**	**ENC_2**	45	31	56.7	69.9	59.8	77.2	-0.5	-0.7	-0.3	0.3	0.005	**
**Control**	**Fringe**	798	11	57.1	85.7	59.7	92.9	-0.7	-0.9	-0.4	0.1	0.005	**
**Mini**	**ENC_2**	491	31	60.4	69.9	62.6	77.2	-0.3	-0.5	-0.1	0.3	0.009	**
**Mini**	**Fringe**	491	11	60.4	85.7	62.6	92.9	-0.7	-0.9	-0.4	0.2	0.012	*
**Micro**	**PICIs**	45	66	56.7	66.9	59.8	72.4	-0.5	-0.7	-0.3	0.3	0.059	ns
**Mini**	**Control**	491	798	60.4	57.1	62.6	59.7	0.1	0.0	0.2	0.5	0.074	ns
**Fringe**	**PICIs**	11	66	85.7	66.9	92.9	72.4	0.7	0.3	1.0	0.9	0.081	ns
**PICIs**	**ENC_4**	66	14	66.9	79.1	72.4	80.0	-0.6	-0.9	-0.3	0.2	0.128	ns
**Mini**	**PICIs**	491	66	60.4	66.9	62.6	72.4	-0.3	-0.4	-0.2	0.4	0.132	ns
**Fringe**	**ENC_2**	11	31	85.7	69.9	92.9	77.2	0.7	0.3	1.0	0.9	0.203	ns
**ENC_2**	**ENC_4**	31	14	69.9	79.1	77.2	80.0	-0.5	-0.8	-0.1	0.3	0.455	ns
**ENC_1**	**ENC_4**	55	14	86.2	79.1	94.0	80.0	0.4	0.0	0.7	0.7	0.719	ns
**Micro**	**Mini**	45	491	56.7	60.4	59.8	62.6	-0.2	-0.3	0.0	0.4	0.885	ns
**Refseq**	**ENC_4**	910	14	84.0	79.1	89.9	80.0	0.3	0.0	0.6	0.6	0.893	ns
**Fringe**	**ENC_4**	11	14	85.7	79.1	92.9	80.0	0.4	-0.1	0.9	0.7	0.975	ns
**Refseq**	**ENC_1**	910	55	84.0	86.2	89.9	94.0	-0.2	-0.3	0.0	0.4	0.976	ns
**PICIs**	**ENC_2**	66	31	66.9	69.9	72.4	77.2	-0.2	-0.5	0.1	0.4	0.985	ns
**Micro**	**Control**	45	798	56.7	57.1	59.8	59.7	-0.1	-0.2	0.1	0.4	1	ns
**Fringe**	**Refseq**	11	910	85.7	84.0	92.9	89.9	0.2	-0.1	0.5	0.6	1	ns
**Fringe**	**ENC_1**	11	55	85.7	86.2	92.9	94.0	-0.2	-0.5	0.0	0.4	1	ns
**Micro**	**ENC_3**	45	4	56.7	98.8	59.8	99.1	-1.0	-1.0	-0.9	0.0	NA	NA
**Mini**	**ENC_3**	491	4	60.4	98.8	62.6	99.1	-1.0	-1.0	-1.0	0.0	NA	NA
**Control**	**ENC_3**	798	4	57.1	98.8	59.7	99.1	-1.0	-1.0	-1.0	0.0	NA	NA
**Fringe**	**ENC_3**	11	4	85.7	98.8	92.9	99.1	-1.0	-1.0	-1.0	0.0	NA	NA
**Refseq**	**ENC_3**	910	4	84.0	98.8	89.9	99.1	-0.9	-1.0	-0.8	0.0	NA	NA
**PICIs**	**ENC_3**	66	4	66.9	98.8	72.4	99.1	-1.0	-1.0	-1.0	0.0	NA	NA
**ENC_1**	**ENC_3**	55	4	86.2	98.8	94.0	99.1	-0.9	-1.0	-0.8	0.0	NA	NA
**ENC_2**	**ENC_3**	31	4	69.9	98.8	77.2	99.1	-1.0	-1.0	-1.0	0.0	NA	NA
**ENC_3**	**ENC_4**	4	14	98.8	79.1	99.1	80.0	0.9	0.7	1.0	1.0	NA	NA

Table S4. Pairwise statistical comparisons for encapsulin fraction (Figure 3C). Pairwise group comparisons for encapsulin fraction values (>0), reporting group sample sizes, group means/medians, Cliff's delta (probability of superiority), and BH-adjusted p-values from Dunn's post-hoc test following a Kruskal–Wallis omnibus test. Source data are provided as a Source Data file.

group1	group2	n1	n2	mean 1	mean 2	median 1	median 2	cliffs_delta	delta_lo	delta_hi	prob_ superiority	p_value	p_stars
**Refseq**	**ENC_2**	17	31	51.1	100.0	52.8	100.0	-1	-1	-1	0	9.72E-12	****
**Refseq**	**ENC_1**	17	55	51.1	99.0	52.8	100.0	-1	-1	-1	0	1.28E-10	****
**Control**	**ENC_2**	20	31	60.0	100.0	64.5	100.0	-0.8	-0.95	-0.6	0.1	6.44E-09	****
**Refseq**	**ENC_4**	17	14	51.1	100.0	52.8	100.0	-1	-1	-1	0	6.85E-09	****
**Control**	**ENC_1**	20	55	60.0	99.0	64.5	100.0	-0.8	-0.9	-0.5	0.1	1.10E-07	****
**Mini**	**Refseq**	93	17	83.4	51.1	100.0	52.8	0.7	0.5	0.8	0.8	5.05E-07	****
**Control**	**ENC_4**	20	14	60.0	100.0	64.5	100.0	-0.8	-1.0	-0.6	0.1	1.26E-06	****
**Mini**	**ENC_2**	93	31	83.4	100.0	100.0	100.0	-0.4	-0.5	-0.3	0.3	0.00021	***
**Mini**	**Control**	93	20	83.4	60.0	100.0	64.5	0.5	0.2	0.7	0.7	0.00022	***
**Mini**	**ENC_1**	93	55	83.4	99.0	100.0	100.0	-0.2	-0.4	-0.1	0.4	0.0052	**
**Mini**	**ENC_4**	93	14	83.4	100.0	100.0	100.0	-0.4	-0.5	-0.3	0.3	0.0072	**
**ENC_1**	**ENC_2**	55	31	99.0	100.0	100.0	100.0	-0.2	-0.3	-0.1	0.4	0.2222	ns
**Control**	**Refseq**	20	17	60.0	51.1	64.5	52.8	0.2	-0.2	0.6	0.6	0.2227	ns
**ENC_1**	**ENC_4**	55	14	99.0	100.0	100.0	100.0	-0.2	-0.3	-0.1	0.4	0.3343	ns
**ENC_2**	**ENC_4**	31	14	100.0	100.0	100.0	100.0	0	0	0	0.5	1	ns
**Mini**	**Fringe**	93	1	83.4	100.0	100.0	100.0	NA	NA	NA	NA	NA	NA
**Mini**	**ENC_3**	93	4	83.4	100.0	100.0	100.0	-0.4	-0.5	-0.3	0.3	NA	NA
**Control**	**Fringe**	20	1	60.0	100.0	64.5	100.0	NA	NA	NA	NA	NA	NA
**Control**	**ENC_3**	20	4	60.0	100.0	64.5	100.0	-0.8	-0.95	-0.6	0.1	NA	NA
**Fringe**	**Refseq**	1	17	100.0	51.1	100.0	52.8	NA	NA	NA	NA	NA	NA
**Fringe**	**ENC_1**	1	55	100.0	99.0	100.0	100.0	NA	NA	NA	NA	NA	NA
**Fringe**	**ENC_2**	1	31	100.0	100.0	100.0	100.0	NA	NA	NA	NA	NA	NA
**Fringe**	**ENC_3**	1	4	100.0	100.0	100.0	100.0	NA	NA	NA	NA	NA	NA
**Fringe**	**ENC_4**	1	14	100.0	100.0	100.0	100.0	NA	NA	NA	NA	NA	NA
**Refseq**	**ENC_3**	17	4	51.1	100.0	52.8	100.0	-1	-1	-1	0	NA	NA
**ENC_1**	**ENC_3**	55	4	99.0	100.0	100.0	100.0	-0.20	-0.31	-0.11	0.40	NA	NA
**ENC_2**	**ENC_3**	31	4	100.0	100.0	100.0	100.0	0	0	0	0.5	NA	NA
**ENC_3**	**ENC_4**	4	14	100.0	100.0	100.0	100.0	0	0	0	0.5	NA	NA
