# Supplementary material for: Small viruses reveal bidirectional evolution between HK97-fold viruses and encapsulins via procapsids
Source: Nat Commun. 2026 Oct 14;17:10571. doi: 10.1038/s41467-026-78136-1 (PMC13669383; doi:10.1038/s41467-026-78136-1)
Supplement: Supplementary file 2 [file 41467_2026_78136_MOESM2_ESM.pdf]

nature portfolio

Corresponding author(s): Antoni Luque

Last updated by author(s): Jun 24, 2026

Reporting Summary

Nature Portfolio wishes to improve the reproducibility of the work that we publish. This form provides structure for consistency and transparency in reporting. For further information on Nature Portfolio policies, see our Editorial Policies and the Editorial Policy Checklist.

Statistics

For all statistical analyses, confirm that the following items are present in the figure legend, table legend, main text, or Methods section.

n/a	Confirmed	
☐	☒	The exact sample size (*n*) for each experimental group/condition, given as a discrete number and unit of measurement
☐	☒	A statement on whether measurements were taken from distinct samples or whether the same sample was measured repeatedly
☐	☒	The statistical test(s) used AND whether they are one- or two-sided<br>*Only common tests should be described solely by name; describe more complex techniques in the Methods section.*
☒	☐	A description of all covariates tested
☐	☒	A description of any assumptions or corrections, such as tests of normality and adjustment for multiple comparisons
☐	☒	A full description of the statistical parameters including central tendency (e.g. means) or other basic estimates (e.g. regression coefficient) AND variation (e.g. standard deviation) or associated estimates of uncertainty (e.g. confidence intervals)
☐	☒	For null hypothesis testing, the test statistic (e.g. *F*, *t*, *r*) with confidence intervals, effect sizes, degrees of freedom and *P* value noted<br>*Give P values as exact values whenever suitable.*
☐	☒	For Bayesian analysis, information on the choice of priors and Markov chain Monte Carlo settings
☒	☐	For hierarchical and complex designs, identification of the appropriate level for tests and full reporting of outcomes
☐	☒	Estimates of effect sizes (e.g. Cohen's *d*, Pearson's *r*), indicating how they were calculated

Our web collection on statistics for biologists contains articles on many of the points above.

Software and code

Policy information about availability of computer code

Data collection	No new experimental data was generated in this study. Data collection consisted of retrieving and annotating existing public sequence and structure datasets. Public datasets included environmental viral contigs from IMG/M, MGnify, public viral/reference sequences from NCBI Viral Genomes/RefSeq, phage-inducible chromosomal island and encapsulin sequences from published sources, and structures from the Protein Data Bank.<br><br>The data collection workflow incorporated CheckV v1.1.1; Prodigal v2.6.3; VPF-PLM v2.0; VIBRANT v1.2.1; KEGG March 2019 release; Pfam v32; VOG release 94; HMMER v3.4; Pfam-A v37; NCBI Conserved Domains v3.18; PHROGs v4; HHpred; and the Protein Data Bank.
Data analysis	Data analysis used CheckV v1.1.1; Prodigal v2.6.3; VPF-PLM v2.0; VIBRANT v1.2.1; KEGG March 2019 release; Pfam v32; VOG release 94; HMMER v3.4; HHpred; MAFFT v6.240 and v7.520; IQ-TREE 2 v2.3.6; TreeCluster v1.0.3; iTOL v6; ChimeraX v1.10.1; AlphaFold2; FoldTree v1.1.0; TrimAl v1.4.rev15; Python 3.10 and R 4.5.2 with shiny 1.11.1, dplyr 1.1.4, ggplot2 4.0.0, mgcv 1.9-3, scales 1.4.0, hexbin 1.28.5, tidyverse 2.0.0, viridis 0.6.5, plotly 4.11.0, rlang 1.1.6, rstatix 0.7.3, FSA 0.10.1, and gridExtra 2.3.<br><br>Custom code and analysis workflows are available at https://github.com/luquelab/hk97-viruses-encapsulins-evolution and archived as version 1.0.0 on Zenodo at https://doi.org/10.5281/zenodo.20802501.

For manuscripts utilizing custom algorithms or software that are central to the research but not yet described in published literature, software must be made available to editors and reviewers. We strongly encourage code deposition in a community repository (e.g. GitHub). See the Nature Portfolio guidelines for submitting code & software for further information.

Data

Policy information about availability of data

All manuscripts must include a data availability statement. This statement should provide the following information, where applicable:

- Accession codes, unique identifiers, or web links for publicly available datasets
- A description of any restrictions on data availability
- For clinical datasets or third party data, please ensure that the statement adheres to our policy

The environmental viral genomes and associated biome metadata generated and compiled in this study (Data Files S1-S4) have been deposited in Figshare under DOI 10.6084/m9.figshare.30943028. The predicted open reading frames and protein annotation data (Data Files S5-S9) have been deposited in Figshare under DOI 10.6084/m9.figshare.30948863. The HK97-fold sequence, structure, and phylogenetic analysis data (Data Files S10-S17) have been deposited in Figshare under DOI 10.6084/m9.figshare.30948881. Public reference data used in this study are available from the NCBI Viral Genomes Resource, NCBI RefSeq, and the Protein Data Bank; relevant identifiers are provided in the Supplementary Information. Source data are provided with this paper.

Research involving human participants, their data, or biological material

Policy information about studies with human participants or human data. See also policy information about sex, gender (identity/presentation), and sexual orientation and race, ethnicity and racism.

Reporting on sex and gender	Sex and gender were not relevant to the analysis.
Reporting on race, ethnicity, or other socially relevant groupings	This study did not analyze human participants, participant-level human data, race, ethnicity, or other socially relevant groupings.
Population characteristics	No human participant population was studied.
Recruitment	No participants were recruited. The study used existing public datasets and public reference resources.
Ethics oversight	No new human participant research, human biological material collection, or identifiable human data analysis was conducted.

Note that full information on the approval of the study protocol must also be provided in the manuscript.

Field-specific reporting

Please select the one below that is the best fit for your research. If you are not sure, read the appropriate sections before making your selection.

☐ Life sciences ☐ Behavioural & social sciences ☒ Ecological, evolutionary & environmental sciences

For a reference copy of the document with all sections, see nature.com/documents/nr-reporting-summary-flat.pdf

Ecological, evolutionary & environmental sciences study design

All studies must disclose on these points even when the disclosure is negative.

Study description	This study is a comparative computational analysis of public environmental viral contigs and reference HK97-fold proteins. The main analytical units were viral contigs, annotated proteins, Pfam domains, and protein structures. The study combines hidden Markov model sequence-profile searches, functional annotation, protein-domain co-occurrence networks, statistical comparisons of domain coverage and encapsulin enrichment, and sequence- and structure-based phylogenetic analyses. There were no experimental treatments, organism manipulations, or prospective biological replicates.
Research sample	The research sample consists of existing public sequence and structure datasets. Environmental viral contigs were identified from public environmental omics data used in CheckV, including IMG/M and MGnify datasets. These data include metagenomic contigs from diverse biomes. The study also uses reference HK97-fold proteins from NCBI Viral Genomes/RefSeq, phage-inducible chromosomal islands, gene transfer agents, published encapsulin datasets, and experimentally determined structures from the Protein Data Bank. No new organisms were collected or experimentally manipulated. The sample is intended to represent public environmental viral sequence diversity enriched for small HK97-fold viral entities, together with relevant HK97-fold viral and encapsulin references.
Sampling strategy	This was a computational sampling strategy based on predefined filtering criteria. Environmental contigs were retained if they contained a direct terminal repeat window of at least 20 bp, contained at least one marker gene associated with tailed phages, and were 20 kb or shorter. Genome-length groups were defined as micro (<5 kb), mini (5-11.6 kb), twilight zone (<11.6 kb), and control (11.6-20 kb), as described in the Methods. Sample size was determined by the number of public sequences passing the pre-specified filters. The resulting datasets were sufficient for the reported comparative domain, network, and phylogenetic analyses.
Data collection	No new field sampling, laboratory experiments, or sequencing were performed. Data collection consisted of downloading, compiling, filtering, and analyzing existing public sequence, annotation, and structure datasets. Derived data were recorded as computational outputs by the authors using the bioinformatic pipelines, databases, and software described in the Methods. The original sequencing instruments and primary sample-collection procedures were those of the source studies and repositories.

Timing and spatial scale	No new field or laboratory sampling was performed, so prospective start and stop dates and sampling frequencies are not relevant to this study. Source-specific sampling dates, sampling frequencies, and geographic metadata are those reported by the original studies and databases. Biome/source metadata used in this study are provided in the deposited data files.
Data exclusions	Contigs were excluded if they lacked a qualifying direct terminal repeat, lacked tailed-phage marker genes, or exceeded the 20 kb length cutoff for the analyzed environmental contig set. Additional filtering was applied for specific analyses, including Pfam-domain E-value thresholds, HHpred probability thresholds, AlphaFold2 pLDDT confidence filtering for structural phylogenetics, and group-size or positive-value filters for statistical comparisons. No additional ad hoc exclusions were applied outside the stated criteria.
Reproducibility	Reproducibility is supported by public deposition of the generated and compiled datasets in Figshare, provision of Source Data with the paper, public availability of the code and analysis workflows in GitHub, and permanent archival of the publication code release in Zenodo. Phylogenetic trees, sequence files, structural files, annotation outputs, and statistical outputs are provided in the Supplementary Information, Source Data, Figshare records, and code repository as described in the Data Availability and Code Availability sections.
Randomization	Random allocation was not relevant because the study did not involve experimental treatments or prospective sample assignment. Samples were assigned to analytical groups using deterministic computational criteria, including genome-length category, environmental/reference source class, and Pfam domain composition.
Blinding	Blinding was not relevant because this was a computational analysis of public sequence and structure datasets. Group allocation was determined by deterministic, pre-specified criteria such as genome length, source/reference class, and detected Pfam domain composition.

Did the study involve field work? ☐ Yes ☒ No

Reporting for specific materials, systems and methods

We require information from authors about some types of materials, experimental systems and methods used in many studies. Here, indicate whether each material, system or method listed is relevant to your study. If you are not sure if a list item applies to your research, read the appropriate section before selecting a response.

Materials & experimental systems

n/a	Involved in the study
☒	☐ Antibodies
☒	☐ Eukaryotic cell lines
☒	☐ Palaeontology and archaeology
☒	☐ Animals and other organisms
☒	☐ Clinical data
☒	☐ Dual use research of concern
☒	☐ Plants

Methods

n/a	Involved in the study
☒	☐ ChIP-seq
☒	☐ Flow cytometry
☒	☐ MRI-based neuroimaging

Plants

Seed stocks	No seed stocks were used.
Novel plant genotypes	No novel plant genotypes were generated or used.
Authentication	No plants were used.
