# Supplementary material for: Small viruses reveal bidirectional evolution between HK97-fold viruses and encapsulins via procapsids
Source: Nat Commun. 2026 Oct 14;17:10571. doi: 10.1038/s41467-026-78136-1 (PMC13669383; doi:10.1038/s41467-026-78136-1)
Supplement: Supplementary file 3 [file 41467_2026_78136_MOESM3_ESM.pdf]

nature portfolio

Peer Review File

Small viruses reveal bidirectional evolution between HK97-fold viruses and encapsulins via procapsids

Corresponding Author: Professor Antoni Luque

This file contains all reviewer reports in order by version, followed by all author rebuttals in order by version.

Version 0:

Reviewer comments:

Reviewer #1

(Remarks to the Author)
Key results:
Authors retrieved small putative viral genomes from metagenome databases that contain a putative capsid protein similar to the canonical viral HK97 capsid protein. They then analyzed these sequences together with encapsulin proteins that have similar structures. Based on these analyses they propose evolutionary scenarios whereby encapsulins and HK97 virus capsid proteins have a common ancestor, which might be related to procapsids.

Validity:
The authors have convincingly discovered some interesting genomes that could be very important for understanding the evolution of encapsulins and HK97-like viruses. I am less convinced by the structural phylogenies and the evolutionary analysis, but I am not an expert in this area. Any evolutionary scenario, particularly for deeply diverging clades, such as these, will be at least to some extent speculative.

Significance:
The origin and evolution of viral capsids and viruses in general is one of the fundamental unanswered questions in Biology. I find this work to be very thought provoking and, crucially, provides clear directions for future study.

Data and methodology:
I am not very familiar with much of the methodology used in this manuscript, so I am not well-qualified to comment on the evolutionary analysis. However, the data used appear to be sound and appropriate.

Analytical approach:
Critical to the analysis presented in this manuscript is the use of structural phylogenies, unfortunately this is not my area of expertise. I am somewhat skeptical of the validity of these analyses and it is not clear to me how statistically significant these analyses are. The main citations cited in the methods are a pre-print from 2023, which apparently has not been published to date (Moi et al., 2023) and a publication that is critical of the methodology (Mutti et al., 2025).
Suggested improvements:
Sentence starting on L56: Suggest including HK-97 here instead of the next paragraph. I find this sentence hard to follow, particularly given the number of citations in the sentence.
L67: “(or tailed phages)” can be removed since it is controversial if viruses that infect archaea can be called phages.
L71: Please provide evidence for the antiquity of these metabolic functions or modify this statement, possibly “putative ancient” or “proposed ancient”.
L74-76: Please provide a citation (possibly Luque et al., 2020) for “the current evolutionary paradigm”. Koonin and Krupovic (2017) state it as “unresolved”.
L80: I disagree that “differences in structural complexity … were not considered” (see Koonin and Krupovic, 2017), but agree that they were not considered in such detail as described in this manuscript. Consider modifying this statement.
L127: There appears to be a typographical error, this should be Salmonella phage astrithr, instead of Astrid.
L99: Suggest repeating HK97 here, e.g. “two main compartments made of HK97-fold containing proteins” or equivalent.
L102: Suggest adding “known” before “Duplodnaviria genome”
L104: Interestingly phage HK-97 scaffolds itself (see Duda and Teschke, 2019). Whether this should be considered as “requiring scaffolding proteins” or not could be considered.

L113: Suggest replacing “Prokaryotic” with “Bacterial”
L138: Please define what is meant by “tailed-like phages”
L139: Please clarify “isolated counterparts”. Are these isolated genomes, genomes from isolated phages or something else?
L143: Please clarify what is meant by “tailed phages”.
L156: Please clarify what is meant by “instances of evolution”
Figure 2: I think that panels F and G could be in supplemental materials as there does not seem to be any major differences between biomes.
L291-292: Please clarify numbers. “58 proteins” does not seem to match (51), (30), (7) and (13).
L351: Consider adding “in viral metagenomes” or “in metagenome datasets” or potentially refer to methods for clarity of the sources of these “twilight zone” viral genomes for readers who do not read the methods.
L400: Consider putting “C) below the graph or repeat the labels to emphasize that the labels (“Environmental”/”Micro”/size) are the same for both panels B and C, if I understand correctly.
L418: I suggest “is predicted to be” instead of “is”.
L439: Description of panel A appears to be missing.
L467: Suggest restating how the genomic origins were determined.
Figure S3: Please clarify what is meant by “Colapsed Each”.
L528/L605: I find “the fact that” to be an overstatement.
L539/L582/L611/L643: I find “well-defined” to be an overstatement.
L591-593: This hypothesis could be more developed.
L606-607: Koonin and Krupovic, 2017 hypotheize that many virus capsid proteins evolved from enzymes. This could be discussed here.

Clarity and context
• Generally manuscript is very well-written. I find the use of “HK97-fold:” “capsid”, “virus/es”, “compartments”, “proteins”, “shells”, “viral genomes”, “fold-encoding genomes” and “Duplodnaviria” to be somewhat confusing. Possibly definitions earlier in the text or more consistency of usage would make this clearer.
• Consider using kbp instead of kb to differentiate these genomes from ssDNA viral genomes.
• I think that a clearer description of what constitutes a viral domain and an encapsulin domain would be very useful for readers. Possibly an extension of Figure 1A where particular regions are designated as “viral” and “encapsulin” or a combination of Figure 1A and Figure 5A. This could clarify the discussion of co-occurrence analysis (Lines 466-476).
• Please clearly define what is meant by “Clades” and “Families” for the structural phylogeny (Lines 484-503). Otherwise, this section is very hard to follow. Possibly a reference to Figure S3 or inclusion of this figure would make this section clearer.

References
References are very good and comprehensive, with the few exceptions noted above.

Your expertise
I am not qualified to review code and I am not an expert on structural phylogeny.

Secondary review from Early Career Researcher: (with which I completely concur).

Suggested Major Revision:
- Structure phylogeny of the HK97-fold proteins is not well supported. The authors claim that the evolution between encapsulins and capsid proteins is bidirectional by combining two complementary analyses: 1) co-occurrence of domains in capsids and encapsulins, and 2) structural phylogenetic analysis. While the co-occurrence of domains confirms the finding that small phages harbor more encapsulin domains than larger phages and strengthen the connections between viruses and encapsulins in their network analysis, the structural phylogeny seems to reflect co-occurrence of domains rather than a credible evolutionary history of these proteins. There is little evidence in the literature that structural phylogenies can be confidently used to infer the evolution of proteins, which may become more problematic with capsid proteins, as they are subject to additional spatial constraints. With the data presented here, it is hard to tell whether the “phylogenetic inference” is reflecting structural changes over evolutionary time rather than structural similarities between proteins that share a particular domain or set of domains.
The result of the presented structural phylogeny should, therefore, be confirmed using phylogenetic analysis of amino acid sequences, as long as sequence similarity allows it. The presence of different domains in capsid proteins and encapsulins may add some difficulty to these analyses. Because of this, a phylogenetic analysis of the most prevalent domains of HK97-fold proteins (e.g. Phage_capsid, Linocin_M18) could be done to confirm the tree topologies of bidirectional evolution obtained through structure comparison. Until this is done, the conclusions obtained from the structural phylogeny alone are not supported and should be reworded.
Suggested Minor Revisions:
- Figure 2G. “Ghost-zone” hasn’t been described in the manuscript. Please correct to “twilight zone”
- Fig. 2G. What do the darker/black bars represent? Please explain in caption. This perhaps explain why the graph does not intuitively show the high occurrence of twilight viral genomes in the human gut compared to other environments (line 357).
- Line 295. Missing T number for 4PT2 and 2E0Z
- Line 368. Why do the authors expect a decrease in amino-acid coverage for MCP from viruses with smaller genomes? This may not be obvious for many readers, are larger capsids assembled from larger MCP proteins? or do they take up too much genomic space? Please explain or remove “as expected”.
- Figure 3.
o The ‘C’ in panel ‘C’ should be bolded.

o Please indicate in the caption what the line in Fig. 3D represents.
- Line 419. Please define “encapsulin-enriched MCPs”, whether it refers to the presence of ≥1 encapsulin domains or a particular fraction.
- Lines 424-432. Much of these data can be found in Fig. 4C. I suggest making this paragraph more concise for clarity of reading.
- Figure 4. The caption seems to correspond to an earlier version of the figure without the tree, as Fig. 4A has no caption and lettering is shifted. Also, Fig.4B’s legend is on at the bottom, not the top right. Please revise the whole caption.
- Line 449. The presence of lysogenic genes suggests, but not definitely indicate, that the virus can be temperate. Please reword.
- Lines 471, 473 and 475. Many of the counts given in these lines do not match the numbers in Figure 5. Please revise and correct accordingly.
- Lines 481-483. Numbers don’t match Figure 5B (88 + 8 vs 84 + 11?). Please clarify
- Lines 485-486 / Figure 5C. The main text refers to 3 clades in 5C from the tree in Fig. S3. However, 4 clades appear in Fig. 5C, none of them labeled with a clade number. Please identify each of the clades in Fig. 5C with the numbers that are used in the main text and in Fig. S3, and refer to, explain or remove the clade with human-gut encapsulin-enriched viruses in Fig. 5C. Also, in the bottom clade, it is unclear whether DTR-named sequences are considered viral here, as one of them contains a Srp1-like encapsulin domain and has not been colored.
- Lines 532-533. This sentence needs rewording: Rep-encoding genetic elements seem to have transitioned between plasmids and viruses by gain-loss of jelly-roll capsids, according to Kazlauskas et al., 2019. The current phrase is a bit misleading.
- Lines 612-622. The protein called RepA in dsDNA phages is not homologous to the Rep protein of CRESS-DNA viruses nor to the protein A of microviruses. Please revise this paragraph considering a possible confusion.

Additional comments:
- Procapsid ancestor. It is indeed really interesting the nexus between encapsulins and capsid proteins through the procapsid, and the possible roles that procapsids may play (or have played) in cellular hosts. However, I find the concept of a procapsid being an “ancestor” problematic when there is no evidence that procapsids are not mere transition states in the formation of mature capsids. Until a stand-alone procapsid is found, it seems to me that they are either immature capsids, or just encapsulins. I think that using the term “ancestral state” would be more appropriate here, as we are unsure of the unique biological functions that a hypothetical “procapsid” alone would have.

(Remarks on code availability)
I am not qualified to evaluate the code.

Reviewer #2

(Remarks to the Author)
In this paper, the authors revisit the evolution of encapsulins, focusing on the protein that encodes encapsulin shells, the HK97-fold major capsid protein (MCP). To help with the task, the authors mined viral metagenomes for small viral genomes that may be packaged into small heads (a dataset that would be useful for researchers beyond this study). The main conclusion of the paper is that the evolution of the MCPs of viruses and encapsulins is intertwined and bidirectional, with the viral ancestor that had a procapsid-like capsid. This is an interesting result that is noteworthy of reporting, but it is, in my opinion, not as “paradigm-shifting” as the authors present it, for two reasons. First, the phylogenetic trees presented in the manuscript, which are based on amino-acid sequences and on structural elements of the MCPs, still support the secondary evolution of encapsulins from viruses, which is what the currently dominant hypothesis is. Second, multiple gene exchange/recombination events have been documented among viruses, as well as between viruses and virus-derived elements, such as GTAs (e.g., PMIDs 30065291 and 21414219), so an occasional mixed ancestry of encapsulins and viruses is not too surprising.

Comments on improving the narrative structure:
The paper is somewhat difficult to read at times, which can be improved by shortening and adjusting the structure of the paper.
1) Because of the Nature Communication article format, the Results section (rather than Methods) will appear after the Introduction section, and so the Results should briefly explain what exact data the authors discuss at a particular place, so that the reader does not need to constantly go to Methods section to try to figure out what dataset is used.
2) Some results are presented for the first time only in the Discussion section and should be moved to the Results section. In particular, the data for the main point of the paper (procapsids first) is presented only in Discussion.
3) The Discussion section could focus solely on the model presented in Figure 6A.

Areas for major revisions:
1) While phylogenetic analyses of the MCP amino acid sequences and structures are very important for the manuscript results and inferences, they are not well presented. First, what are the trees rooted with? After all, clades can only be defined on rooted trees. Second, the branches should be scaled with respect to substitutions. Third, bootstrap support values should be shown on the branches/nodes.

2) It is also not clear what diversity of MCPs does amino-acid-sequence-based tree represents in comparison to the structure-based tree. It would be nice to have taxa present in both datasets highlighted on the trees, especially given that structure-based methods are still being developed and validated (as discussed in the Mutti et al 2025 paper cited in the

manuscript). Congruence in the relationships of MCPs present both trees would increase reader's confidence of the inferences from the structure-based tree.

3) Figure 3C: the fraction of encapsulin domains in mini viruses seems bimodal, including the large number of them being fully encapsulin-like, thus the median number isn't too representative to claim the trend. Of 62 viruses whose MCPs are fully encapsulin-like, how do we know that they are viruses and not encapsulins? And where are these MCPs on the phylogenetic trees?

4) Figure 3B: the phage_coat coverages for environmental phage datasets have huge ranges and their distribution among the micro phages is clearly bimodal. So, I feel that the median values aren't representative. Also, is the reported decline in coverage in small viruses significant? (No statistics provided.)

5) The data about distribution of different twilight zone viruses and encapsulins across biomes seems to distract from the main message of the study. Plus, human gut samples are no doubt overrepresented in the public databases, so there is probably a representation bias that needs to be corrected for. Remove?

6) The supplementary data associated with the manuscript should be published in a data repository like FigShare and in someone's personal Google Drive.

Other comments:
1) Lines 90-94: The description of the T-number can go into Figure 1 legend, as it disrupts the flow of the narrative here. Also, either define h and k or drop T(h,k) formula.
2) Lines 128-133: why are the mentioned T=3 phages and phage-derived structures not included in Figure 1B among the viral capsid sizes?
3) Lines 134-136: I am not sure why the hypothesis of "encapsulins first" needs to be listed here, as this is not what authors test. If the authors wanted to refer to any earlier work that possibly explored this hypothesis, a citation should be provided.
4) Lines 154-155: what do the authors refer to when they say "molecularly related"? Aren't all the analyzed proteins here are homologous and thus "related"?
5) Figure 2D: What GTA proteins were included? The two references provided are from PICIs. There are two distinct MCPs in two described types of alphaproteobacterial GTAs (PMID: 36381234).
6) Figure 2G, "ghost-zone": did the authors mean to state "twilight zone"?
7) Lines 203-207, definition of the "twilight zone": On line 203, it is defined as 5-11.6 kb, but then divided into < 5kb (micro) and 5-11.6kb (mini) ones. So, should the "twilight zone" be defined as 0-11.6 kb?
8) Line 239: what is this dataset of 60 genomes? Unclear.
9) What statistical test was employed for the results shown in Figure 3C?
10) Figure 3D, the solid line: What does a polynomial function summarize across encapsulins and viruses combined? Not described in the figure legend or text.
11) In my opinion, the full tree topology (Figure S3) and not the tree snippets (Figure 5C) should be displayed in the main manuscript.
12) Line 334-335: GTAs are not taxonomically restricted to Rhodobacter (see PMIDs 28784044 and 36381234).
13) Line 384: what is the "fringe" zone?
14) It is confusing that clades are referred using Roman numerals in the text, while in the Figure S3 they are labeled with Arabic numerals.
15) Conclusions, line 643: which of the discussed clades do the authors refer to here?

(Remarks on code availability)
This is the first time I see the link to the code - it should be listed in the manuscript.

Reviewer #3

(Remarks to the Author)
I co-reviewed this manuscript with one of the reviewers who provided the listed reports. This is part of the Nature Communications initiative to facilitate training in peer review and to provide appropriate recognition for Early Career Researchers who co-review manuscripts.

(Remarks on code availability)
I reviewed the python code in the GitHub repository. It works quite ok and easily runnable from CLI. Some details about what each particular script does is lacking, but I for reproducibility purposes I found all information to be sufficient.

Reviewer #4

(Remarks to the Author)
I co-reviewed this manuscript with one of the reviewers who provided the listed reports. This is part of the Nature Communications initiative to facilitate training in peer review and to provide appropriate recognition for Early Career Researchers who co-review manuscripts.

(Remarks on code availability)

Version 1:

Reviewer comments:

Reviewer #1

(Remarks to the Author)
This review is in response to the revised version of the manuscript.

Thanks to the authors for their revisions, I think that it improved the manuscript.

By its nature, this work is somewhat speculative, but I think that the authors have made a strong argument and have presented some intriguing models for future research.

My only major comment is that the authors state in line 435 of the marked up manuscript "the tree topology differed slightly from that in the structure-based analysis" in regard to Figure S6. I disagree that the topology of the two trees is only slightly different, it appears quite different to me. Perhaps "somewhat" instead of "slightly" or omit "slightly" or reword this sentence.

Minor comments:
Throughout the manuscript be careful about use of italics. Virus names themselves should not be italicized according to the ICTV (https://ictv.global/faq/names), only formal taxonomic ranks (e.g. family, genus, species) should be italicized.

L123: "Dashed red box" appears to be missing from figure in the integrated PDF.
L166: There is an additional period after "viruses."
L367: Please check to make sure that analyzed is not removed in the revised text (it is both underlined and struck through in the marked up manuscript).
L518: The Helix-Turn-Helix motif (HTH) generally refers to a DNA-binding motif. These structures do not appear to be related to that motif, so consider using different terminology, 2 long alpha helicies, for instance.

(Remarks on code availability)
I am not qualified to assess the code.

Reviewer #2

(Remarks to the Author)
The revised manuscript has improved substantially. I have several follow-up comments that arose when the authors addressed some of my earlier concerns.

Major comments
1) Mid-point rooting, while convenient, does not necessarily root a tree at the most ancestral node, as the technique is very sensitive to differences in rates of evolution on different branches (something one expects in dataset that contains cellular and viral proteins). Unfortunately, that also means that the mid-point rooted tree is essentially should be treated as unrooted and thus does not allow to make inferences about ancestor-descendant relationships. This is important for two inferences in the manuscript:
a. To define “clades” one needs to know ancestor-descendant relationships on a tree. One solution would be to avoid term “clade” and use word “group”.
b. Figures 6B and 6D (the model) use mid-point rooted tree to make inferences about which group was ancestral.
2) Aside from mid-point rooting, the phylogenetic tree for which bootstrap support values are shown (Figure S4) demonstrates very poor resolution for most “clades” (and for the deepest branches on the tree), which is OK for the overall conclusions of the manuscript (the intertwined evolution of encapsulins and viruses), but not good for scenario shown on Figure 6B. Also, the structure-based tree (no statistical support provided) essentially has deep-branches of length 0, and thus suggests that there is no resolution among various groups on this tree, again making directional inferences on figures 6B and 6D speculative.
3) Comment on “RESPONSE 2.8 Trees in the revised manuscript display branch lengths proportional to the underlying distance metric: substitutions per site for sequence-based trees and structural distances for structure-based trees. For the sequence-based tree, bootstrap support values were included.”
a. The scale units of the trees should be listed in legends of figures S6 and Figure 5. Scales must have units! For the structure trees, what are the units? Did the authors use 3Di encodings to make the trees, or some other structural distance metric?
b. Statistical support values should be shown on all trees (Fig 4, Fig 5, Fig S6), including for structure-based tree. Based on Figure S6, the structural tree is so different from the amino-acid sequence tree, but I suspect this is because structure-based tree has very low resolution on group-defining branches.

Minor comments:
p. 12, line 238: typo - remove “while”.
p.20, line 412: “unrelated” should be replaced with “divergent” or “poorly conserved”, as unrelated means no common

ancestor and that contradicts the main argument of the paper (shared ancestry of encapsulins and viral capsids).
Figure 2C: Predicted T-numbers. Should any values between $1<T<3$ be assigned to T=3, as there are now capsids with fractional T number?

(Remarks on code availability)

Reviewer #3

(Remarks to the Author)
I co-reviewed this manuscript with one of the reviewers who provided the listed reports. This is part of the Nature Communications initiative to facilitate training in peer review and to provide appropriate recognition for Early Career Researchers who co-review manuscripts.

(Remarks on code availability)
The GitHub looks good now, the code works as expected.

Reviewer #4

(Remarks to the Author)
I co-reviewed this manuscript with one of the reviewers who provided the listed reports. This is part of the Nature Communications initiative to facilitate training in peer review and to provide appropriate recognition for Early Career Researchers who co-review manuscripts.

(Remarks on code availability)

Version 2:

Reviewer comments:

Reviewer #1

(Remarks to the Author)
Thank you for your modifications and clarifications.

(Remarks on code availability)

Reviewer #2

(Remarks to the Author)
All my concerns were satisfactorily addressed in the second revision.

(Remarks on code availability)

Reviewer #3

(Remarks to the Author)
I co-reviewed this manuscript with one of the reviewers who provided the listed reports. This is part of the Nature Communications initiative to facilitate training in peer review and to provide appropriate recognition for Early Career Researchers who co-review manuscripts.

(Remarks on code availability)

Reviewer #4

(Remarks to the Author)
I co-reviewed this manuscript with one of the reviewers who provided the listed reports. This is part of the Nature Communications initiative to facilitate training in peer review and to provide appropriate recognition for Early Career Researchers who co-review manuscripts.

(Remarks on code availability)

Open Access This Peer Review File is licensed under a Creative Commons Attribution 4.0 International License, which permits use, sharing, adaptation, distribution and reproduction in any medium or format, as long as you give appropriate credit to the original author(s) and the source, provide a link to the Creative Commons license, and indicate if changes were made.
In cases where reviewers are anonymous, credit should be given to 'Anonymous Referee' and the source.
The images or other third party material in this Peer Review File are included in the article's Creative Commons license, unless indicated otherwise in a credit line to the material. If material is not included in the article's Creative Commons license and your intended use is not permitted by statutory regulation or exceeds the permitted use, you will need to obtain permission directly from the copyright holder.
To view a copy of this license, visit https://creativecommons.org/licenses/by/4.0/

RESPONSE TO REVIEWERS

General note. The response to reviewers lists the specific changes made in the manuscript. The cited line numbers refer to the marked-up manuscript, which highlights changes in red and includes deleted text in strike-through. A clean version of the manuscript without highlighting has been provided to facilitate readability.

Reviewer #1 (Remarks to the Author):

Key results:

Authors retrieved small putative viral genomes from metagenome databases that contain a putative capsid protein similar to the canonical viral HK97 capsid protein. They then analyzed these sequences together with encapsulin proteins that have similar structures. Based on these analyses they propose evolutionary scenarios whereby encapsulins and HK97 virus capsid proteins have a common ancestor, which might be related to procapsids.

Validity:

The authors have convincingly discovered some interesting genomes that could be very important for understanding the evolution of encapsulins and HK97-like viruses. I am less convinced by the structural phylogenies and the evolutionary analysis, but I am not an expert in this area. Any evolutionary scenario, particularly for deeply diverging clades, such as these, will be at least to some extent speculative.

Significance:

The origin and evolution of viral capsids and viruses in general is one of the fundamental unanswered questions in Biology. I find this work to be very thought provoking and, crucially, provides clear directions for future study.

Data and methodology:

I am not very familiar with much of the methodology used in this manuscript, so I am not well-qualified to comment on the evolutionary analysis. However, the data used appear to be sound and appropriate.

Analytical approach:

REVIEWER 1.1
Critical to the analysis presented in this manuscript is the use of structural phylogenies, unfortunately this is not my area of expertise. I am somewhat skeptical of the validity of these analyses and it is not clear to me how statistically significant these analyses are. The main citations cited in the methods are a pre-print from 2023, which apparently has not been published to date (Moi et al., 2023. and a publication that is critical of the methodology (Mutti et al., 2025).

RESPONSE 1.1
We thank the reviewer for the thorough evaluation and feedback. The method used in the structure-based phylogeny, FoldTree, has now been peer-reviewed and published in Nature Structural & Molecular Biology (Moi et al., 2025). The publication addresses the critiques raised in Mutti et al. (2025). FoldTree performs comparably to state-of-the-art

sequence-based methods and shows a clear advantage for divergent proteins that share a conserved fold, as in our case for HK97-fold proteins. In fact, prior HK97-fold protein evolutionary studies virus attempted evolutionary reconstructions using structural comparisons, for example, using DALI Z-scores or HSF-derived structural distance trees (Andreas & Giessen, 2021; Krupovic & Koonin, 2017; Podgorski et al., 2023). However, FoldTree provides a better, principled structural phylogenetic framework that (i) retains sensitivity to fine-grained local structural differences and (ii) has been benchmarked to perform comparably to maximum-likelihood sequence methods on more closely related families while yielding more robust topologies for deeply divergent, structurally conserved lineages. Nonetheless, the revised manuscript also includes a sequence-based phylogeny of the same set of HK97-fold proteins analyzed with FoldTree. Both approaches recover virus-to-encapsulin and encapsulin-to-virus transitions.

CHANGES 1.1
The lines cited refer to the marked-up manuscript, which highlights changes in red and includes deleted text in strike-through. This applies to all the changes in the rebuttal.
Lines 398 to 400: “The large structural phylogenetic analysis was performed with FoldTree […] conserved structure (Moi et al., 2025).”
Lines 437 to 440, Results section: “Protein folds change more slowly than amino-acid sequences […] consistent with recent benchmarks of structural phylogenetics (Moi et al., 2025).”
Lines 917 to 929: New Methods subsection titled "Sequence-based phylogeny."
New Supplementary Figures: Figure S4 (sequence-based trees with branch support values), Figure S5 (structural- and sequence-based clades comprising viral and encapsulin proteins), and Figure S6 (structural- and sequence-based trees comparison) were added.

Suggested improvements:

REVIEWER 1.2
Sentence starting on L56: Suggest including HK97 here instead of the next paragraph. I find this sentence hard to follow, particularly given the number of citations in the sentence.

RESPONSE 1.2
We have streamlined the narrative of this fragment, describing the HK97-fold proteins as the protein lineage of interest, as suggested.

CHANGES 1.2
Lines 56 to 63, Introduction section: “One intriguing group of proteins are HK97-fold proteins [...] they have been overlooked in the debate about the origin of life.”

REVIEWER 1.3
L67: “(or tailed phages)” can be removed since it is controversial if viruses that infect archaea can be called phages.

RESPONSE 1.3
The revised manuscript uses "tailed prokaryotic viruses" rather than "tailed phages" when referring to *Duplodnaviria* infecting Archaea and Bacteria. The terminology adopted aligns with the ICTV-accepted nomenclature (Adriaenssens et al., 2025) and recent publications in the field (Evseev et al., 2023; Zhao et al., 2024). We have used the designation “HK97-

fold viruses" as a broader term for viral entities that encode capsid proteins adopting the HK97-fold.

CHANGES 1.3
Line 69: The term "tailed prokaryotic viruses" is used instead of "tailed phage", referring to *Duplodnaviria* viruses that infect Bacteria and Archaea.
Lines 153, 652, 736, 740, 844: The term "tailed phage" is used, referring to *Duplodnaviria* viruses that infect bacteria.
All instances of the term "tailed phages" have been reviewed. The designation "HK97-fold viruses" is now used whenever referring to viral entities that encode capsid proteins adopting the HK97-fold.

REVIEWER 1.4
L71: Please provide evidence for the antiquity of these metabolic functions or modify this statement, possibly "putative ancient" or "proposed ancient".

RESPONSE 1.4
The revised text uses the term "proposed ancient," as suggested. References supporting the ancient origins of the listed functions have been incorporated. We include some insights from these references below, but we have not included these explanations in the manuscript to keep it concise:

- Iron metabolism. Williams (2012) reviews the biological roles of iron since the prebiotic era, including iron storage proteins such as ferritin, which are related to encapsulins in Thermotoga maritima (LaFrance *et al.*, 2021).
- Oxidative stress response. It has been hypothesized that the Great Oxygenation Event (GOE) occurred ~2.3 billion years ago (Lyons *et al.,* 2014), drastically changing planetary conditions and triggering multiple adaptive processes.
- Sulfur metabolism. Mateos et al. (2023) provided evidence that the timing of the emergence of ancient sulfur-cycling genes is linked to the redox state of early Earth.

CHANGES 1.4
Lines 78 to 79: "These functions have been proposed as ancient (Cardenas et al., 2016; Kim et al., 2012; Mateos et al., 2023; R. J. P. Williams, 2012)."

REVIEWER 1.5
L74-76: Please provide a citation (possibly Luque et al., 2020. for "the current evolutionary paradigm". Koonin and Krupovic (2017. state it as "unresolved".

RESPONSE 1.5
The revised manuscript now states that Koonin and Krupovic 2017 indicated that the evolutionary relationship was unresolved. The revised text also clarifies that Luque et al. (2020) framed the evolutionary problem from a structural-complexity perspective, and that Andreas and Giessen (2021) compared novel encapsulin systems with HK97-fold viruses, suggesting that encapsulins evolved from HK97-fold viruses.

CHANGES 1.5
Lines 80 to 85: "Initial sequence and structural analyses [...] either inconclusive about their origin or favored viral HK97-fold proteins as ancestral, given their presence across more lineages (Krupovic & Koonin, 2017). A more recent and exhaustive phylogenetic and structural evolutionary study […] indicated that encapsulins are likely derived from HK97-fold viruses (Andreas & Giessen, 2021)."

REVIEWER 1.6

L80: I disagree that “differences in structural complexity … were not considered” (see Koonin and Krupovic, 2017), but agree that they were not considered in such detail as described in this manuscript. Consider modifying this statement.

RESPONSE 1.6

The revised manuscript notes that while Koonin and Krupovic (2017) compared HK97-fold proteins structurally, they did provide a model to explain how HK97-fold shells evolved metabolic functions from genomic ones or how complex HK97-fold viruses could have preceded encapsulins.

CHANGES 1.6

Lines 85 to 94: “However, the evolutionary model […] does not provide a rationale for how HK97-fold shells evolved metabolic functions from shells that carried genomic functions, or for how known HK97-fold viruses [...] would have emerged before encapsulins. Prior evolutionary models noted, but did not explicitly incorporate, the differences in structural complexity between viral capsids and encapsulins [...] which typically form small T=1 or T=3 shells (Andreas & Giessen, 2021; Krupovic & Koonin, 2017).”

REVIEWER 1.7

L127: There appears to be a typographical error, this should be Salmonella phage astrithr, instead of Astrid.

RESPONSE 1.7

The typographical error has been fixed.

CHANGES 1.7

Line 154: “*Salmonella phage astrithr*”.

REVIEWER 1.8

L99: Suggest repeating HK97 here, e.g. “two main compartments made of HK97-fold containing proteins” or equivalent.

RESPONSE 1.8

We have edited the sentence as recommended.

CHANGES 1.8

Lines 121 to 122: “The gray boxes describe the two main compartments made of HK97-fold proteins”.

REVIEWER 1.9

L102: Suggest adding “known” before “*Duplodnaviria* genome”

RESPONSE 1.9

The recommendation was implemented in the text.

CHANGES 1.9

Lines 129 to 130: “The vertical line marks the smallest known *Duplodnaviria* genome as a reference.”

REVIEWER 1.10
L104: Interestingly phage HK-97 scaffolds itself (see Duda and Teschke, 2019). Whether this should be considered as “requiring scaffolding proteins” or not could be considered.

RESPONSE 1.10
As the reviewer points out, the HK97 phage capsid protein's delta domain serves as a scaffold. This domain is cleaved during maturation, released, and acts as a scaffold protein (Duda et al., 1995; Huang et al., 2011). We have revised the text to clarify that, in viruses with HK97-fold capsid proteins, scaffolding can be provided either by dedicated scaffold proteins or by cleavable scaffolding domains, and to contrast this maturation with the simpler assembly of encapsulins.

CHANGES 1.10
Lines 108 to 113: “Unlike encapsulin assembly [...] the maturation of HK97-fold viruses is more complex [...], it involves scaffold proteins (sometimes fused with the HK97-fold), portal proteins to facilitate DNA translocation, a terminal complex to ensure packaging, and often [...] minor proteins reinforcing the structure (Dearborn et al., 2017; Duda et al., 1995; Fokine & Rossmann, 2014; Hardy et al., 2020; Morais et al., 2003; Podgorski et al., 2025).”

REVIEWER 1.11
L113: Suggest replacing “Prokaryotic” with “Bacterial and archaeal viruses”

RESPONSE 1.11
The revised text uses "Bacterial and archaeal viruses" instead of “Prokaryotic”.

CHANGES 1.11
Lines 137 to 138: “Bacterial and archaeal viruses”

REVIEWER 1.12
L138: Please define what is meant by “tailed-like phages”

RESPONSE 1.12
The term “tailed-like phage” was used in the original manuscript to describe the widespread entities identified in metagenomic studies that exhibit characteristics typical of bacterial and archaeal *Duplodnaviria* viruses. However, the revised text uses "HK97-fold viruses" instead of "tailed-like phages" to be more accurate.

CHANGES 1.12
Lines 170 to 172: “First, genomes associated with HK97-fold viruses are prevalent when sequencing environmental samples”

REVIEWER 1.13
L139: Please clarify “isolated counterparts”. Are these isolated genomes, genomes from isolated phages or something else?

RESPONSE 1.13
Yes, the phrase referred to genomes from isolated *Duplodnaviria* viruses. The revised sentence states that explicitly.

CHANGES 1.13

Line 170 to 174: “First, genomes associated with HK97-fold viruses are prevalent when sequencing environmental samples and display a much higher diversity than the genomes from isolated *Duplodnaviria* viruses (Cobián Güemes et al., 2016; Koonin et al., 2024; Paez-Espino et al., 2016; Wigington et al., 2016; Wommack & Colwell, 2000).”

REVIEWER 1.14

L143: Please clarify what is meant by “tailed phages”.

RESPONSE 1.14

In that context, we referred to a subset of genomes containing HK97-fold proteins in which Direct Terminal Repeats (DTRs) were used as a proxy to identify complete genomes. In the revised text, we used the more accurate term "HK97-fold viruses."

CHANGES 1.14

Lines 176 to 178: “Third, HK97-fold viruses can encode direct terminal repeats [...] providing a bioinformatic proxy for identifying complete genomes”

REVIEWER 1.15

L156: Please clarify what is meant by “instances of evolution”

RESPONSE 1.15

We revised the text to use the more common term “transition events,” similarly to Kazlauskas et al. (2019), which described comparable evolutionary processes as transitions: “Thus, transitions between the virus and plasmid states appear to be bidirectional” ~Kazlauskas et al (2019).

CHANGES 1.15

Lines 192 to 194: “The newly identified HK97-fold proteins revealed at least four transition events between encapsulins and HK97-fold viruses, suggesting bidirectional evolution.”

REVIEWER 1.16

Figure 2: I think that panels F and G could be in supplemental materials as there does not seem to be any major differences between biomes.

RESPONSE 1.16

We have moved Figures 2F and 2G to the supplementary materials as recommended.

CHANGES 1.16

Figures 2F and 2G are now part of Figure S1.

REVIEWER 1.17

L291-292: Please clarify numbers. “58 proteins” does not seem to match (51), (30), (7. and (13).

RESPONSE 1.17

The issue has been corrected. In the original submission, the total count reflected an earlier version of the encapsulin reference set. The revised manuscript now uses 104 encapsulin protein sequences to better match their known diversity across phyla. Running the phylogenetic analyses with this set did not change the qualitative results: Families I and III still form distinct clades, and all virus–encapsulin relationships described in the Results are preserved.

CHANGES 1.17
Lines 833 to 836: “In total, 104 encapsulin proteins were retrieved, ensuring representativity across different phyla [...] family I (55 proteins), family II (31), family III (4), and family IV (14).”

REVIEWER 1.18
L351: Consider adding “in viral metagenomes” or “in metagenome datasets” or potentially refer to methods for clarity of the sources of these “twilight zone” viral genomes for readers who do not read the methods.

RESPONSE 1.18
The revised text uses the term “metagenomic datasets” as recommended.

CHANGES 1.18
Lines 234 to 235: “We identified 4,730 putatively complete viral genomes in the twilight zone (< 11.6 kbp) from metagenomic datasets”

REVIEWER 1.19
L400: Consider putting “C. below the graph or repeat the labels to emphasize that the labels (“Environmental”/”Micro”/size. are the same for both panels B and C, if I understand correctly.

RESPONSE 1.19
We repeated the labels for panels B and C as recommended.

CHANGES 1.19
Line 264: Figure 3C.

REVIEWER 1.20
L418: I suggest “is predicted to be” instead of “is”.

RESPONSE 1.20
The text has been revised as suggested.

CHANGE 1.20
Lines 314 to 315: “The dominant clade of encapsulin-enriched viral genomes is predicted to be lysogenic and lacks a portal or tail apparatus”

REVIEWER 1.21
L439: Description of panel A appears to be missing.

RESPONSE 1.21
We have addressed this error and added the description of panel A.

CHANGES 1.21
Lines 353 to 354: “A) Phylogeny of environmental HK97-fold proteins. Branches in gray highlight groups containing encapsulin domains.”

REVIEWER 1.22
L467: Suggest restating how the genomic origins were determined.

RESPONSE 1.22
The results section now states that HK97-fold proteins were classified as viral or encapsulin based on the origin of the genome encoding each protein.

CHANGES 1.22
Line 373 to 376: “HK97-fold proteins encoded in viral genomes (environmental contigs passing the CheckV viral filters or, *Duplodnaviria* genomes, GTAs, or PICIs from RefSeq) were labeled as viral. Proteins encoded in curated encapsulin operons were labeled as encapsulins.”

REVIEWER 1.23
Figure S3: Please clarify what is meant by “Colapsed Each”.

RESPONSE 1.23
This erroneous wording has been removed. The full structural tree is now presented in Figure 5C.

CHANGES 1.23
Line 446: Figure 5

REVIEWER 1.24
L528/L605: I find “the fact that” to be an overstatement.

RESPONSE 1.24
The revised text now uses the term “observation” instead of “fact”.

CHANGES 1.24
Lines 530 to 532: “This bidirectional evolutionary paradigm is further supported by our observations indicating that HK97-fold protein domains [...] form a fully connected co-occurrence network”
Lines 594 to 595: “The observation that viral infections often generate empty capsids or procapsids (Laurenceau et al., 2020; Subramanian et al., 2025)”

REVIEWER 1.25
L539/L582/L611/L643: I find “well-defined” to be an overstatement.

RESPONSE 1.25
The revised text describes these sequences more neutrally, as a subset of mini genomes encoding encapsulin-enriched HK97-fold proteins and as candidates for follow-up work, without implying that they form a fully resolved lineage.

CHANGES 1.25
Lines 550 to 554: “This hypothesis is based on [...] and the presence of lysogenic genes in the subset of mini genomes encoding encapsulin-enriched HK97-fold proteins”
Line 604: “The group of encapsulin-enriched mini genomes reported here lacked a recognizable portal”

REVIEWER 1.26
L591-593: This hypothesis could be more developed.

RESPONSE 1.26
We expanded this section to make the hypothesis more explicit and mechanistically grounded, proposing an evolutionary biophysical trajectory from small, low-symmetry HK97-fold shells to T=1 and then to larger dsDNA capsids.

CHANGES 1.26
Lines 549 to 582, Discussion: "One compelling possibility is that the procapsid state observed in HK97-fold viruses has facilitated such transitions (Figure 6C). This hypothesis is based on the structural similarity of encapsulins and procapsids compartments (Figure 6A), the co-assembly and structural parallels of cargo and viral scaffold proteins (Figure 6C), and the presence of lysogenic genes in the subset of mini genomes encoding encapsulin-enriched HK97-fold proteins discovered in this study (Figure 4). The presence of lysogeny-associated genes indicates the potential for genomic integration into host chromosomes (Howard-Varona et al., 2017; Luque & Silveira, 2020). Integration increases the opportunity for recombination between viral HK97-fold shells and host-encoded cargo-like proteins, favoring the emergence of encapsulin compartments from viral ancestors. The same setting could also facilitate recombination events that link encapsulin shells to scaffolding and portal genes, allowing encapsulin compartments to reacquire genome-packaging functions and move back toward a virus-like lifestyle. Together with the shared HTH motif of cargo and scaffold proteins (Figure 6A), these observations support a mechanistic model in which the internal protein of the HK97-fold shell acts as a switch between biochemical and genetic roles. The observation that the few isolated *Duplodnaviria* viruses containing encapsulin domains also encode lysogeny-related genes further supports this scenario (Figure 3). Additionally, encapsulins and procapsids form shells that are more porous and display bulgier capsomers (pentamers and hexamers) than mature capsids (Giessen & Silver, 2017; Hryc et al., 2017; Selivanovitch et al., 2021; Wu et al., 2016). The pores enable encapsulins to transport reactants (ions or other molecules) necessary for biochemical functions (Chmelyuk et al., 2023; Ross et al., 2022; Williams et al., 2018). In fact, the pores in procapsids have facilitated their use as biochemical reactors in engineering applications (Selivanovitch et al., 2021; Wang & Douglas, 2022). The parallels between viral procapsids and encapsulins suggest a flexible, bidirectional model in which the identity of the internal functional (cargo/scaffold) protein helps determine the role of an HK97-fold shell (Figure 6C). If the internal partner is an enzymatic cargo, the compartment acts as a biochemical reactor, concentrating substrates and protecting intermediates. If it is a scaffold that favors the interaction with the portal proteins, the shell is instead expanded, the scaffold released, and a dsDNA genome can be packaged. Thus, switching the identity of the internal protein would determine if an HK97-fold compartment operates as a biochemical reactor (encapsulin-like) or a genome storage and delivery entity (virus-like). Under such a bidirectional regime, repeated switches between these states are expected to blur a simple, single-origin signal for either purely biochemical or purely genetic HK97-fold compartments."

REVIEWER 1.27
L606-607: Koonin and Krupovic, 2017 hypotheize that many virus capsid proteins evolved from enzymes. This could be discussed here.

RESPONSE 1.27
The revised text explicitly discusses the hypothesis of Koonin & Krupovic (2017) that many capsid proteins arose by recruitment from cellular enzymes and nanocompartment

proteins. The text explains how our proposed model (Figure 6E) is compatible with that view.

CHANGES 1.27
Lines 583 to 590: "This virus-encapsulin transition is consistent with [...]. It is also compatible with the proposal of Koonin and Krupovic (2017) that many capsid proteins evolved repeatedly by recruitment from cellular enzymes and nanocompartment proteins. In this context, the cargo/scaffold proteins provide a natural candidate for enzyme-derived precursors, whose activities can be redeployed in viral or cellular settings depending on how they interact with HK97-fold shells."

Clarity and context:

REVIEWER 1.28
Generally manuscript is very well-written. I find the use of "HK97-fold:" "capsid", "virus/es", "compartments", "proteins", "shells", "viral genomes", "fold-encoding genomes" and "*Duplodnaviria*" to be somewhat confusing. Possibly definitions earlier in the text or more consistency of usage would make this clearer.

RESPONSE 1.28
We reviewed the manuscript to ensure consistent use of key terms. We also included a fragment clarifying the definitions of some terms at the beginning of the Results section.

CHANGES 1.28
Lines 203 to 215, Key terminology: "The following terms are summarized here to facilitate the interpretation of the results [...] and are used here as encapsulin-like sequence signatures within HK97-fold proteins."

REVIEWER 1.29
Consider using kbp instead of kb to differentiate these genomes from ssDNA viral genomes.

RESPONSE 1.29
The revised text uses kbp when referring to the lengths of double-stranded DNA genomes. It uses kb only in contexts where ssDNA genomes are explicitly discussed.

CHANGES 1.29
Genome-length units were standardized across the main text, figure captions, and supplementary materials.

REVIEWER 1.30
I think that a clearer description of what constitutes a viral domain and an encapsulin domain would be very useful for readers. Possibly an extension of Figure 1A where particular regions are designated as "viral" and "encapsulin" or a combination of Figure 1A and Figure 5A. This could clarify the discussion of co-occurrence analysis (Lines 466-476).

RESPONSE 1.30
The revised manuscript clarifies that the term "domain" is used in two independent contexts: one structural and one sequence based. The structural domains of the HK97-fold (A-domain, P-domain, E-loop) are conserved across viruses and encapsulins. Protein domains, with respect to sequences, are those defined by hidden Markov models (HMMs).

The "viral" or "encapsulin" domains in this case refer to those originally identified in viruses and encapsulins before this study. We have revised the text accordingly to avoid confusion. We have also added a supplementary figure (Figure S2) to place the HMM domains in the context of the HK97-fold structural domains and to clarify the parallels between Figures 1A and 5A.

CHANGES 1.30
Figure S2 was added to the supplementary materials.
Lines 211 to 215, Key terminology: "Structural domains: regions of the HK97 fold defined from three-dimensional capsid architecture (Figure S2). Sequence domains: Domains identified in protein sequences by matches to hidden Markov model (HMM) profiles in the Pfam database (Figure S2); encapsulin domains are Pfam domains built from encapsulin shell proteins and are used here as encapsulin-like sequence signatures within HK97-fold proteins."

REVIEWER 1.31
Please clearly define what is meant by "Clades" and "Families" for the structural phylogeny (Lines 484-503). Otherwise, this section is very hard to follow. Possibly a reference to Figure S3 or inclusion of this figure would make this section clearer.

RESPONSE 1.31
In the manuscript, "Clades" refers to groups in the trees that share a common ancestor, following the standard operational interpretation in phylogenetic analysis (Wilkinson et al., 2007). The term "Family" is used to describe the principal categories of encapsulins, following the current nomenclature in the field Giessen et al., 2021. A brief description of the operational use of clades and the standard encapsulin Families I, II, III, and IV is included in the new fragment about terminology in the Results.

CHANGES 1.31
Lines 405 to 406: "Encapsulin families (I–IV) are referred to according to the classification proposed by Andreas & Giessen (2021)."

References:

REVIEWER 1.32
References are very good and comprehensive, with the few exceptions noted above.

RESPONSE 1.32
The missing references were added.

CHANGES 1.32
Sections 1.4, 1.5, and 1.6.

Your expertise:

I am not qualified to review code and I am not an expert on structural phylogeny.

Secondary review from Early Career Researcher (ECR):

Suggested Major Revision:

REVIEWER ECR 1.1
Structure phylogeny of the HK97-fold proteins is not well supported. The authors claim that the evolution between encapsulins and capsid proteins is bidirectional by combining two complementary analyses: 1. co-occurrence of domains in capsids and encapsulins, and 2. structural phylogenetic analysis. While the co-occurrence of domains confirms the finding that small phages harbor more encapsulin domains than larger phages and strengthen the connections between viruses and encapsulins in their network analysis, the structural phylogeny seems to reflect co-occurrence of domains rather than a credible evolutionary history of these proteins. There is little evidence in the literature that structural phylogenies can be confidently used to infer the evolution of proteins, which may become more problematic with capsid proteins, as they are subject to additional spatial constraints. With the data presented here, it is hard to tell whether the "phylogenetic inference" reflects structural changes over evolutionary time rather than structural similarities among proteins that share a particular domain or set of domains.

RESPONSE ECR 1.1
In the revised manuscript, we clarify that our structural analysis relies on FoldTree, a recent peer-reviewed method published by Moi et al. in Nature Structural & Molecular Biology (2025). This method introduces an explicit evolutionary model using a benchmarked structural alphabet. FoldTree performs comparably to state-of-the-art sequence-based approaches for closely related families and provides more reliable topologies for deeply divergent, structurally conserved lineages, as in the HK97-fold proteins in our study. Prior HK97-fold protein studies compared structures using DALI Z-score matrices or dendrograms. Our use of FoldTree offers a more principled and validated structural phylogenetic framework.

Nonetheless, we have added a sequence-based phylogeny built from the same HK97-fold protein dataset. The sequence-based analysis shows congruence with the structure-based tree, supporting bidirectional encapsulin-virus transitions. The revised text discusses the limitations of structure-based inference and includes a comparison with the sequence-based phylogeny (Figures S4, S5, and S6).

CHANGES ECR 1.1
The lines cited refer to the marked-up manuscript, which highlights changes in red and includes deleted text in strike-through. This applies to all the changes in the rebuttal.
Lines 398 to 400: "The large-scale structural phylogenetic analysis was performed with FoldTree […] conserved structure (Moi et al., 2025)."
Lines 437 to 440: "Protein folds change more slowly than amino-acid sequences, so structural information is typically more reliable for deeper relationships, whereas sequence-based trees provide finer resolution among recently derived lineages, consistent with recent benchmarks of structural phylogenetics (Moi et al., 2025)."
Lines 917 to 929: New Methods subsection titled "Sequence-based phylogeny."
New figures: Figures S4, S5, and S6

REVIEWER ECR 1.2
The result of the presented structural phylogeny should, therefore, be confirmed using phylogenetic analysis of amino acid sequences, as long as sequence similarity allows it. The

presence of different domains in capsid proteins and encapsulins may add some difficulty to these analyses. Because of this, a phylogenetic analysis of the most prevalent domains of HK97-fold proteins (e.g. Phage_capsid, Linocin_M18. could be done to confirm the tree topologies of bidirectional evolution obtained through structure comparison. Until this is done, the conclusions obtained from the structural phylogeny alone are not supported and should be reworded.

RESPONSE ECR 1.2
As suggested, we have performed a standard amino-acid sequence phylogenetic analysis using the same HK97-fold protein set as in the structural tree. The sequence-based tree recovers multiple mixed lineages of viral and encapsulin HK97-fold proteins. Most groups are congruent with the groups in the structural tree, supporting our main conclusion. The methods and results sections were updated accordingly, and new figures were added in the supplementary.

CHANGES ECR 1.2
Lines 432 to 445, Results section: “The sequence-based phylogeny was performed as an independent method to identify viral-encapsulin transitions [...] The sequence-based analysis also recovered three clades mixing viruses and encapsulins that were observed in the structure-based phylogeny [...]. The overall congruence between the structural and sequence-based trees strengthened the evidence for multiple bidirectional transitions across viral and encapsulin lineages”.
Lines 917 to 929, Methods section: “Sequence-based phylogeny. [...]. The resulting tree is available in Supplementary Figure S4 [...].”
New supplementary figures: Figures S4, S5, and S6.

Suggested Minor Revisions:

REVIEWER ECR 1.3
Figure 2G. “Ghost-zone” hasn’t been described in the manuscript. Please correct to “twilight zone”

RESPONSE ECR 1.3
The correct term is "twilight zone." We removed "Ghost-zone".

CHANGES ECR 1.3
Figure 2G was moved to Supplementary Figure S1 and was labeled as “Percentages of twilight-zone phage contigs across biomes.”

REVIEWER ECR 1.4
Fig. 2G. What do the darker/black bars represent? Please explain in caption. This perhaps explain why the graph does not intuitively show the high occurrence of twilight viral genomes in the human gut compared to other environments (line 357).

RESPONSE ECR 1.4
The darker bars show the fraction of contigs associated with micro-contigs (< 5 kbp). We have revised the caption to include this information and moved this panel to the Supplementary Materials, following another reviewer's suggestion (REVIEWER 1.16). It is now part of Figure S1.

CHANGES ECR 1.4
Figure S1 and associated caption.

REVIEWER ECR 1.5
Line 295. Missing T number for 4PT2 and 2E0Z

RESPONSE ECR 1.5
The T-numbers were added. Both structures display a T=3.

CHANGES ECR 1.5
Lines 838 to 839: “encapsulins forming [...] T=3 (4PT2, 2E0Z)”

REVIEWER ECR 1.6
Line 368. Why do the authors expect a decrease in amino-acid coverage for MCP from viruses with smaller genomes? This may not be obvious for many readers, are larger capsids assembled from larger MCP proteins? or do they take up too much genomic space? Please explain or remove “as expected”.

RESPONSE ECR 1.6
The HMM profiles associated with HK97-fold proteins in the Pfam phage_coat clan were built mainly from MCPs of isolated HK97-fold viruses, which typically have genomes longer than 20 kbp. MCPs from smaller, uncultured genomes are therefore more likely to be novel relative to these profiles and only partially matched by them, so a decrease in amino-acid coverage with decreasing genome size was expected. This rationale has been clarified in the Results section.

CHANGES ECR 1.6
Lines 255 to 259: “The HK97-fold proteins from the NCBI Reference Sequence Database (RefSeq) group displayed the most extensive coverage, with a median of 95% (Figure 3B). This was expected because the phage-coat Pfam clan has been historically built from proteins of isolated viruses in RefSeq, whereas HK97-fold proteins from uncultured viruses are more divergent and therefore less likely to be fully captured by existing Pfam profiles.”

REVIEWER ECR 1.7
Figure 3. The ‘C’ in panel ‘C’ should be bolded.

RESPONSE ECR 1.7
We have fixed this formatting issue.

CHANGES ECR 1.7
Line 264: Figure 3C.

REVIEWER ECR 1.8
Figure 3. Please indicate in the caption what the line in Fig. 3D represents.

RESPONSE ECR 1.8
The solid black line represents a nonparametric smoothing spline (generalized additive model) that shows the qualitative trend between the fraction of encapsulin domains and viral genome length. This has been described in the caption as requested.

CHANGES ECR 1.8
Line 281: “The black curve is a generalized additive model (thin-plate regression spline) used for a visual guide.”

Lines 862 to 866: “To visualize the relationship between encapsulin domain fraction [...] The smoother was used only as a descriptive aid and was not used for hypothesis testing.”

REVIEWER ECR 1.9
Line 419. Please define “encapsulin-enriched MCPs”, whether it refers to the presence of ≥1 encapsulin domains or a particular fraction.

RESPONSE ECR 1.9
We have clarified that “encapsulin-enriched MCPs” refers to MCPs containing one or more (≥1) encapsulin HMM profiles (domains) in their sequence.

CHANGES ECR 1.9
Line 294 to 295: “Their encapsulin-enriched MCPs, defined as those displaying one or more encapsulin domains”

REVIEWER ECR 1.10
Lines 424-432. Much of these data can be found in Fig. 4C. I suggest making this paragraph more concise for clarity of reading.

RESPONSE ECR 1. 10
We made the text more concise as requested. We now refer readers to panel Figure 4C and the Supplementary Material for the additional details.

CHANGES ECR 1. 10
Lines 326 to 329: “The only structural proteins identified were the HK97-fold MCP and scaffold protein [...] corresponded to a single open reading frame (DTR_497289_3), annotated as a tail tape measure protein.”

REVIEWER ECR 1.11
Figure 4. The caption seems to correspond to an earlier version of the figure without the tree, as Fig. 4A has no caption and lettering is shifted. Also, Fig.4B’s legend is on at the bottom, not the top right. Please revise the whole caption.

RESPONSE ECR 1.11
We have revised the figure caption to address this issue as requested.

CHANGES ECR 1.11
Lines 353 to 363: “Figure 4. [...] A) Phylogeny of twilight zone major capsid proteins. Branches in gray highlight groups containing encapsulin domains [...] The pie sections include the average percentages.”

REVIEWER ECR 1.12
Line 449. The presence of lysogenic genes suggests, but not definitely indicate, that the virus can be temperate. Please reword.

RESPONSE ECR 1.12
We have revised the wording as recommended.

CHANGES ECR 1.12

Lines 341 to 343: “The few *Duplodnaviria* viruses from the NCBI Reference Sequence Database (RefSeq) that contained encapsulin domains in their MCPs also encode lysogeny-related genes, suggesting they may be temperate.”

REVIEWER ECR 1.13
Lines 471, 473 and 475. Many of the counts given in these lines do not match the numbers in Figure 5. Please revise and correct accordingly.

RESPONSE ECR 1.13
The counts in the text and in Figure 5A have been corrected. The discrepancy came from using an intermediate version of the dataset when generating the original counts. In the revised version, the counts were regenerated using the final, non-redundant dataset, including an expanded encapsulin subset that better reflects the known distribution of encapsulin families across phyla. These updates do not change the qualitative patterns reported before.

CHANGES ECR 1.13
Lines 382 to 388: “Viral HK97-fold MCPs (2,321) formed one dominant group with only viral domains (n = 2,190 or 94%) and two minor groups: one with mixed viral and encapsulin domains (n = 69 or 3%) and the other containing only encapsulin domains (n = 62 or 3%). Encapsulin HK97-fold proteins (104) formed two groups: the dominant group displayed only encapsulin domains (n = 83, 80%), and the smaller one combined viral and encapsulin domains (n = 21, 20%). No encapsulin HK97-fold proteins were found to contain only viral domains.”
Figure 5A: Counts were updated.

REVIEWER ECR 1.14
Lines 481-483. Numbers don’t match Figure 5B (88 + 8 vs 84 + 11?). Please clarify

RESPONSE ECR 1.14
We have revised the numbers to ensure consistency between the text and Figure 5B. The correct numbers are 86 and 11.

CHANGES ECR 1.14
Lines 393 to 394: “In the full network, the *Linocin_M18* and *Srpl-like* encapsulin domains exhibited, respectively, 86 and 11 non-canonical connections with viral domains"

REVIEWER ECR 1.15
Lines 485-486 / Figure 5C. The main text refers to 3 clades in 5C from the tree in Fig. S3. However, 4 clades appear in Fig. 5C, none of them labeled with a clade number. Please identify each of the clades in Fig. 5C with the numbers that are used in the main text and in Fig. S3, and refer to, explain or remove the clade with human-gut encapsulin-enriched viruses in Fig. 5C. Also, in the bottom clade, it is unclear whether DTR-named sequences are considered viral here, as one of them contains a Srp1-like encapsulin domain and has not been colored.

RESPONSE ECR 1.15
We have updated the clade numbers and ensured they are consistent across the figures and text. DTR-named sequences are considered of viral origin based on the annotation of characteristic viral genes, but they contain HK97-fold proteins displaying encapsulin HMM profiles.

CHANGES ECR 1.15

Lines 407 to 431: " Structural Clade 1 grouped Family II encapsulins with terrestrial viral MCPs and isolated phages [...]; SrpI-like encapsulin domains occur in both encapsulins and viral MCPs, whereas GP7 domains are restricted to viral MCPs, consistent with an encapsulin-like ancestor and later GP7 recruitment. Clade 2 contained two virus–encapsulin mixtures [...], where the prevalence of phage_capsid domains suggests a viral ancestor for Family III encapsulins. Clade 9 grouped archaeal Family IV encapsulins with phages infecting Salmonella and Bordetella, with ubiquitous Linocin_M18 domains suggesting an encapsulin-like ancestor. MCPs from mini genomes with encapsulin fraction = 1 occur in structural clades 4 and 7 (Figure 5C), where they appear as viral leaves whose adjacent domain symbols consist exclusively of encapsulin domains."

REVIEWER ECR 1.16

Lines 532-533. This sentence needs rewording: Rep-encoding genetic elements seem to have transitioned between plasmids and viruses by gain-loss of jelly-roll capsids, according to Kazlauskas et al., 2019. The current phrase is a bit misleading.

RESPONSE ECR 1.16

The sentence was rewritten to align more closely with the mechanism described by Kazlauskas et al. (2019) and to avoid suggesting that Rep-encoding elements themselves directly "transition" between plasmids and viruses. The revised text now frames this as bidirectional or mixed ancestry between viruses and mobile elements and explicitly describes gain and loss of jelly-roll capsid genes in plasmids with Rep proteins related to those of ssDNA viruses.

CHANGES ECR 1.16

Lines 538 to 547: "Yet bidirectional or mixed ancestry between viruses and virus-derived elements is not unprecedented [...]. For example, plasmids with replication initiator proteins (Reps) closely related to those of ssDNA viruses have repeatedly given rise to new virus lineages through the acquisition of single jelly-roll capsid protein genes, while some plasmids have been proposed to derive from viruses via the loss of the capsid proteins (Kazlauskas et al., 2019). Thus, bidirectionality between HK97-fold viral and cellular compartments aligns with that observed in other viral capsid folds."

REVIEWER ECR 1.17

Lines 612-622. The protein called RepA in dsDNA phages is not homologous to the Rep protein of CRESS-DNA viruses nor to the protein A of microviruses. Please revise this paragraph considering a possible confusion.

RESPONSE ECR 1.17

The revised manuscript clarifies that the Rep/RepA-like proteins discussed belong to distinct families within the broader HUH endonuclease superfamily and should not be interpreted as a single homologous protein family. We described specific genes with conserved nicking and strand-transfer activities and framed them as candidate factors that could couple replication and DNA translocation in the absence of portals and tails, by analogy to ssDNA systems. The possibility of HK97-fold entities packaging ssDNA is described as a mechanistic hypothesis that requires experimental confirmation.

CHANGES ECR 1.17

Lines 612 to 628: “Several genomes encode proteins annotated as rolling-circle replication initiators (Rep/RepA-like). The proteins belong to the broader HUH endonuclease superfamily [...] via a Rep-mediated mechanism.”

Additional comments:

REVIEWER ECR 1.18

Procapsid ancestor. It is indeed really interesting the nexus between encapsulins and capsid proteins through the procapsid, and the possible roles that procapsids may play (or have played. in cellular hosts. However, I find the concept of a procapsid being an “ancestor” problematic when there is no evidence that procapsids are not mere transition states in the formation of mature capsids. Until a stand-alone procapsid is found, it seems to me that they are either immature capsids, or just encapsulins. I think that using the term “ancestral state” would be more appropriate here, as we are unsure of the unique biological functions that a hypothetical “procapsid” alone would have.

RESPONSE ECR 1.18

Following the reviewers’ observations, the revised manuscript presents a more detailed evolutionary model that examines the phenotype of ancestral HK97-fold shells, grounded in biophysical and structural observations. We introduced a “biochemical-first” scenario, in which small, low-symmetry shells initially functioned as metabolic nanocompartments, and a “genomic-first” scenario, in which the earliest HK97-fold shells were T=1 capsids. In this context, procapsids are not considered "ancestors"; rather, they are the nexus between encapsulins and viral capsids, facilitating bidirectional transitions between them.

CHANGES ECR 1.18

Lines 660 to 698, Discussion section: “Building on the procapsid nexus model (Figure 6D), which could explain the bidirectional transition between viruses and encapsulins, and the possibility of ssDNA HK97-fold twilight viruses (Figure 4), the final question concerns the ancestral form of HK97-fold compartments. A speculative biophysical scenario of increasing complexity is proposed in Figure 6D. In this scenario, HK97-fold shells may have emerged as small, low-symmetry tetrahedral or octahedral shells. It has been observed that single-point mutations in the HK97-fold protein of the T=3 (180-mer) encapsulin of *Myxococcus xanthus* can lead to the formation of T=1 (60-mer) and a tetrahedral (36-mer) architecture (Szyszka et al., 2024). Tetrahedral and octahedral capsids form local thermodynamic minima in biophysical models, and their reduced number of capsid proteins makes them kinetically advantageous (Aznar & Reguera, 2016; Luque et al., 2012; Zandi et al., 2004). Thus, the early evolution of tetrahedral or octahedral HK97-fold capsids is biophysically sound. However, such shells have a small internal volume; the structurally reconstructed tetrahedral HK97-fold shell displays an internal volume of ~300 nm3, which is ~6.5 times smaller than the equivalent T = 1 shells and 40 times less than the volume of the wild-type T =3 (Szyszka et al., 2024). At quasicrystal densities, such a tetrahedral shell could accommodate 150 bp of dsDNA or 300 nt of ssDNA (Luque et al., 2020). This could encode a protein, but it is at least three times smaller than the smallest DNA viral genome known (Campillo-Balderas et al., 2015; Díez-Villaseñor & Rodriguez-Valera, 2019). However, there is evidence of biochemical compartments with volumes below 300 nm3 that enclose Fe2+ oxidation (Hagen, 2024; Zhang & Orner, 2011), which is also present in encapsulins (Andreas & Giessen, 2021; He et al., 2016). Thus, low-symmetry HK97-fold compartments would likely have carried biochemical reactions. Larger capsids are thermodynamically more favorable (Zandi et

al., 2004). This, along with the formation of specialized biochemical domains in the interior-facing regions of HK97-fold proteins that later became cargo proteins ("proto-cargo" HK97-fold proteins), would have favored increases in size, yielding T=1 capsids with sufficient volume to enclose minimal genomes (Figure 6D). Consistent with patterns of protein domain evolution, catalytic domains within such ancestral proto-cargo proteins could then have emancipated as independent proteins, forming early encapsulin-like nanocompartments (Aziz & Caetano-Anollés, 2021; Toll-Riera & Albà, 2013). The internal T=1 volume may have simultaneously favored the translocation of single-stranded DNA, as in other T=1 capsid systems (Mankertz & Hillenbrand, 2001; Sonntag et al., 2011; Kejzar et al., 2022), leading to early HK97-fold twilight zone viruses. Eventually, scaffold-like proteins may have favored further increases in capsid size and interactions with portal proteins, giving rise to the more complex dsDNA *Duplodnaviria* viruses (Figure 6D). At the same time, because HK97-fold shells appear to have repeatedly switched between encapsulin- and virus-states, our current sampling cannot determine whether HK97-fold compartments for genome packaging or biochemical roles emerged first, and recurrent transitions may have partially eroded the signature of the earliest steps. This speculative yet parsimonious biophysical scenario for the ancestry and evolution of HK97-fold compartments integrates the various elements identified in this study, providing an evolutionary model with specific predictions and testable hypotheses for future studies."

Reviewer #1 (Remarks on code availability):

I am not qualified to evaluate the code.

Reviewer #2 (Remarks to the Author):

REVIEWER 2.1

In this paper, the authors revisit the evolution of encapsulins, focusing on the protein that encodes encapsulin shells, the HK97-fold major capsid protein (MCP). To help with the task, the authors mined viral metagenomes for small viral genomes that may be packaged into small heads (a dataset that would be useful for researchers beyond this study). The main conclusion of the paper is that the evolution of the MCPs of viruses and encapsulins is intertwined and bidirectional, with the viral ancestor that had a procapsid-like capsid. This is an interesting result that is noteworthy of reporting, but it is, in my opinion, not as “paradigm-shifting” as the authors present it, for two reasons.

RESPONSE 2.1

We thank the reviewer for the thorough review and for recognizing that our manuscript presents an interesting result worthy of reporting. Regarding the "paradigm-shifting" comment, we have revised our language and clarified that a key conceptual advance in the field concerns the evolutionary molecular mechanism (procapsid and scaffold/cargo exchange) to explain the bidirectional evolution of biochemical and genomic HK97-fold compartments.

CHANGES 2.1

The lines cited refer to the marked-up manuscript, which highlights changes in red and includes deleted text in strike-through. This applies to all the changes in the rebuttal.

Lines 546 to 549: "Thus, bidirectionality between HK97-fold viral and cellular compartments aligns with that observed in other viral capsid folds. These parallels make it relevant to ask which molecular mechanisms could enable HK97-fold shells to repeatedly transition between viral and encapsulin states.”

REVIEWER 2.2

First, the phylogenetic trees presented in the manuscript, which are based on amino-acid sequences and on structural elements of the MCPs, still support the secondary evolution of encapsulins from viruses, which is what the currently dominant hypothesis is.

RESPONSE 2.2

We agree that our phylogenetic trees include transitions compatible with scenarios in which encapsulins evolved secondarily from viruses, as previously reported. We have revised the text to clarify that. We also clarified in the manuscript that a key result is the observation of encapsulin to virus transitions, which, combined with the other transition, led to the interpretation of bidirectional transitions and the possible mechanism via procapsid (cargo/scaffold switch) as the nexus facilitating this transition.

CHANGES 2.2

Lines 366 to 371, Results section: “To infer the evolutionary relationship between encapsulins and HK97-fold viruses [...]. Our results suggest bidirectional evolution of HK97-fold viruses and encapsulins.”

Lines 419 to 423, Results section: “Clade 2 also grouped encapsulins from Family III, characteristic of soil bacteria, mini viral genomes [...] derived from a viral ancestor.”

REVIEWER 2.3

Second, multiple gene exchange/recombination events have been documented among viruses, as well as between viruses and virus-derived elements, such as GTAs (e.g., PMIDs 30065291 and 21414219), so an occasional mixed ancestry of encapsulins and viruses is not too surprising.

RESPONSE 2.3
We agree that horizontal gene transfer and recombination are pervasive in viral evolution. The revised manuscript places our work more clearly within this broader context. The novelty lies in evidence of bidirectional evolution, the specific molecular mechanisms fueling the transitions, and the potential functional implications of viral procapsids. This places HK97-fold shells in a continuous evolutionary and functional space linking mechanistically genome-packaging capsids and metabolically active compartments.

CHANGES 2.3
Lines 538 to 549: “Yet bidirectional or mixed ancestry between viruses and virus-derived elements is not unprecedented [...] These parallels make it relevant to ask which molecular mechanisms could enable HK97-fold shells to repeatedly transition between viral and encapsulin states.”
Lines 549 to 582: “One compelling scenario possibility is that the procapsid state observed in HK97-fold viruses has facilitated such transitions [...]. Under such a bidirectional regime, repeated switches between these states are expected to blur a simple, single-origin signal for either purely biochemical or purely genetic HK97-fold compartments.”

Comments on improving the narrative structure:

REVIEWER 2.4
The paper is somewhat difficult to read at times, which can be improved by shortening and adjusting the structure of the paper.
1. Because of the Nature Communication article format, the Results section (rather than Methods. will appear after the Introduction section, and so the Results should briefly explain what exact data the authors discuss at a particular place, so that the reader does not need to constantly go to Methods section to try to figure out what dataset is used.

RESPONSE 2.4
We revised the text to improve its readability. We have moved the Results section after the Introduction as recommended. The Results section now includes a brief description of “Key terminology” to facilitate the interpretation of the results.

CHANGES 2.4
Lines 204 to 215: "The following terms are summarized here to facilitate the interpretation of the results [...] encapsulin domains are Pfam domains built from encapsulin shell proteins and are used here as encapsulin-like sequence signatures within HK97-fold proteins."
Lines 234 to 235: “We identified 4,730 putatively complete viral genomes in the twilight zone (< 11.6 kbp) from metagenomic datasets”
Lines 247 to 250: “The working hypothesis [...]. This was tested by scanning HK97-fold proteins for the presence of encapsulin hidden Markov model (HMM) sequence domains (Figure 3 and S2).”
Lines 316 to 317: “To assess if encapsulin-enriched MCPs from environmental genomes comprise a single group, a sequence phylogeny was conducted.”

Lines 366 to 370: “To infer the evolutionary relationship between encapsulins and HK97-fold viruses, the HK97-fold proteins were analyzed using three complementary methods: [...] and sequence-based phylogenetic analysis.”
Lines 465 to 468: “To infer the potential mechanisms underlying the bidirectional evolution [...] structure of encapsulins were compared with those of viral HK97-fold capsids and their immature state, procapsids. This was possible thanks to the existence of reconstructed T=4 architectures for encapsulins, capsids, and procapsids (Figures 6A and S7).”

REVIEWER 2.5
2. Some results are presented for the first time only in the Discussion section and should be moved to the Results section. In particular, the data for the main point of the paper (procapsids first. is presented only in Discussion.

RESPONSE 2.5
Following the reviewer's recommendation, we have moved the text associated with the quantitative and structural comparisons that underpin the “procapsid” model into a subsection of the Results section. We have rearranged the panels in Figure 6 to be consistent with the description of the structural results before the evolutionary model, as addressed in the Discussion section.

CHANGES 2.5
Lines 867 to 875, Methods section: “Full compartment comparisons. To test whether encapsulin compartments resemble viral procapsids [...] bacteriophage *Φ29* scaffold protein dimer (PDB 8F2M).”
Lines 464 to 481: “Viral procapsids and encapsulins show close structural similarity [...] and facilitates scaffold protein release (Hua et al., 2017; Veesler & Johnson, 2012).”

REVIEWER 2.6
The Discussion section could focus solely on the model presented in Figure 6A.

RESPONSE 2.6
As recommended by the reviewer, the Discussion now focuses on the phylogenetic evolutionary model presented in Figure 6B (previously Fig. 6A). The evolutionary model has been updated and expanded in response to other reviewers' recommendations.

CHANGES 2.6
Lines 549 to 582, Discussion section: “One compelling possibility [...] Thus, switching the identity of the internal protein would determine if an HK97-fold compartment operates as a biochemical reactor (encapsulin-like) or a genome storage and delivery entity (virus-like) [...] for either purely biochemical or purely genetic HK97-fold compartments.”

Areas for major revisions:

REVIEWER 2.7
While phylogenetic analyses of the MCP amino acid sequences and structures are very important for the manuscript results and inferences, they are not well presented. First, what are the trees rooted with? After all, clades can only be defined on rooted trees.

RESPONSE 2.7
In the revised manuscript, both the structure-based and sequence-based HK97-fold trees are explicitly midpoint-rooted, because no suitable external outgroup is known for HK97-

fold proteins. In this setting, “clades” are defined on the midpoint-rooted trees, but the mixed virus–encapsulin groups highlighted in the Results are determined by local bipartitions and remain the same across reasonable rooting. The term “clade” was used to refer to groups in a rooted tree for which all members are more closely related to each other than any member is to any non-member (Wilkinson et al., 2007). The global root mainly affects directional statements (for example, which clade is inferred to be more ancestral), but the conclusions about multiple mixed virus–encapsulin groups do not rely on such statements. An arbitrary choice of a viral or encapsulin lineage as outgroup was therefore avoided, as it would have polarized the tree toward one direction of evolution, whereas midpoint rooting provides a neutral compromise.

CHANGES 2.7
Lines 454 to 455, Figure 5 legend: “C) Structural tree of HK97-fold proteins. Midpoint-rooted tree comparing encapsulins, twilight-zone viral MCPs, and RefSeq representatives.”
Lines 914 to 916: “Given the absence of an appropriate external outgroup for HK97-fold proteins, the resulting structural tree was rooted by midpoint rooting.”
Lines 927 to 929: “Because no clear external outgroup could be defined for the HK97-fold dataset, the maximum-likelihood tree was rooted using midpoint rooting.”

REVIEWER 2.8
Second, the branches should be scaled with respect to substitutions. Third, bootstrap support values should be shown on the branches/nodes.

RESPONSE 2.8
Trees in the revised manuscript display branch lengths proportional to the underlying distance metric: substitutions per site for sequence-based trees and structural distances for structure-based trees. For the sequence-based tree, bootstrap support values were included.

CHANGES 2.8
Figures 5, S4, and S6 include scaled branch lengths with scale bars.
Figure S4 (new sequence-based tree) includes support values.

REVIEWER 2.9
It is also not clear what diversity of MCPs does amino-acid-sequence-based tree represents in comparison to the structure-based tree. It would be nice to have taxa present in both datasets highlighted on the trees, especially given that structure-based methods are still being developed and validated (as discussed in the Mutti et al 2025 paper cited in the manuscript). Congruence in the relationships of MCPs present both trees would increase reader’s confidence of the inferences from the structure-based tree.

RESPONSE 2.9
The revised manuscript now makes a direct comparison between the structure-based and sequence-based tree (Figure S6), as both trees are inferred from the same set of HK97-fold proteins, so the diversity of MCPs represented is directly comparable. Figure 5C shows the complete midpoint-rooted structural tree, recomputed using the published FoldTree implementation (Moi et al., Nature Structural & Molecular Biology, 2025). The corresponding maximum-likelihood amino acid sequence tree for the same proteins is presented in Supplementary Figure S4. To highlight taxa present in both trees and to assess congruence, Figure S5 shows matched subsets of the structural and sequence

trees, and Figure S6 displays the full trees side-by-side with colored bands linking corresponding clades. Both trees recover the mixed virus–encapsulin groups and the same three key clades discussed in the Results (structural clades 1, 2, and 9), with most differences confined to tip-level branching order. This pattern is consistent with recent benchmarks showing that structure-based trees are more stable at deep divergence, whereas sequence-based trees provide finer resolution near the tips.

CHANGES 2.9
Lines 917 to 929, Methods section: "Sequence-based phylogeny. To provide an independent validation [...] we conducted a standard amino-acid sequence phylogenetic analysis using the same set of HK97-fold proteins [...] Branch support was calculated using ultrafast bootstrap approximation with 1,000 replicates [...] the maximum-likelihood tree was rooted using midpoint rooting."
Figure 5: Updated to display the full midpoint-rooted structural tree of HK97-fold proteins, calculated with the published FoldTree implementation (Moi et al., 2025)
Supplementary Figures S4, S5, and S6 (new figures).

REVIEWER 2.10
Figure 3C: the fraction of encapsulin domains in mini viruses seems bimodal, including the large number of them being fully encapsulin-like, thus the median number isn't too representative to claim the trend. Of 62 viruses whose MCPs are fully encapsulin-like, how do we know that they are viruses and not encapsulins? And where are these MCPs on the phylogenetic trees?

RESPONSE 2.10
Figure 3C was revised to better reflect the bimodal distribution of encapsulin fractions in mini genomes. Median bars were removed to emphasize the full distribution, and the trend is now supported using a Kruskal–Wallis test followed by Dunn's post-hoc pairwise comparisons with Benjamini–Hochberg correction. The adjusted p-values are reported in Supplementary Table S4.

For the MCPs from mini genomes whose phage_coat coverage is associated exclusively with encapsulin domains ("encapsulin fraction = 1"), the revised Results now clarify that they are treated as viral because they are encoded in contigs that (i) passed CheckV viral filters, (ii) carry additional *Duplodnaviria* hallmark genes, and (iii) do not occur in an encapsulin operon context (Figure 4B). As with any metagenomic dataset, classification of individual contigs is probabilistic, and this general limitation is now stated explicitly. The revised text indicates where these fully encapsulin-like MCPs fall in the phylogenies: in the structural tree (Figure 5C), they are located primarily in structural clades 4 and 7, as viral leaves, with adjacent domain symbols consisting exclusively of encapsulin domains.

CHANGES 2.10
Figure 3C: Significance bars with asterisk annotations for specific pairwise contrasts were added.
Lines 858 to 862: "Because the distribution of encapsulin fraction showed strong non-normality [...] Pairwise adjusted p-values are reported in Supplementary Table S4."
Supplementary Table S4 (new): Group differences and adjusted p-values.
Lines 429 to 431: "The MCPs from mini genomes with encapsulin fraction of 1 occur in structural clades 4 and 7 (Figure 5C), where they appear as viral leaves whose adjacent domain symbols consist exclusively of encapsulin domains."
Lines 287 to 290: Results section: "These MCPs are encoded on contigs that pass CheckV viral filters, carry additional *Duplodnaviria* hallmark genes, and lack the operon context

typical of encapsulins (Figure 4B). We therefore treated them as viral MCPs with encapsulin-like domains, while noting that some metagenomic elements may represent misclassified systems."

REVIEWER 2.11
Figure 3B: the phage_coat coverages for environmental phage datasets have huge ranges and their distribution among the micro phages is clearly bimodal. So, I feel that the median values aren't representative. Also, is the reported decline in coverage in small viruses significant? (No statistics provided.)

RESPONSE 2.11
The revised manuscript now includes formal statistics for Fig. 3B. Phage_coat coverage differences among groups were tested using Welch's one-way ANOVA, followed by Games–Howell post-hoc comparisons to account for unequal n. The results show a strong overall group effect (Welch ANOVA, $p < 2.2\times10^{-16}$) and highly significant reductions in phage_coat coverage in environmental groups relative to RefSeq (Supplementary Table S3). We removed lines above the median to emphasize the full distribution (violin).

CHANGES 2.11
Figure 3B: Significance bars with asterisk annotations for prespecified pairwise contrasts were added.
Lines 855 to 858: "The difference in phage-coat coverage between groups was assessed [...] Supplementary Table S3 report Games–Howell adjusted p-values."
Supplementary Table S3 (new): Includes group differences and adjusted p-values.

REVIEWER 2.12
The data about distribution of different twilight zone viruses and encapsulins across biomes seems to distract from the main message of the study. Plus, human gut samples are no doubt overrepresented in the public databases, so there is probably a representation bias that needs to be corrected for. Remove?

RESPONSE 2.12
The biome distribution data were moved from Figure 2 to Figure S1 in the Supplementary Information, as recommended.

CHANGES 2.12
Figure 2G was moved to Supplementary Figure S1 and was labeled as "Percentages of twilight-zone phage contigs across biomes."

REVIEWER 2.13
The supplementary data associated with the manuscript should be published in a data repository like FigShare and in someone's personal Google Drive.

RESPONSE 2.13
All supplementary datasets have been deposited in Figshare. The revised manuscript includes the links in the Data Availability section.

CHANGES 2.13
Lines 932 to 940: "All environmental viral genomes analyzed here and their associated biome metadata (Data Files S1–S4) are available in the Figshare collection

"Environmental viral genomes (sequences from JGI IMG/VR)" (https://doi.org/10.6084/m9.figshare.30943028). Predicted open reading frames and all protein-level annotation outputs (Data Files S5–S9) are available in "Protein function annotation (ORFs and annotation outputs)" (https://doi.org/10.6084/m9.figshare.30948863). HK97-fold sequence and structure resources and phylogenies (Data Files S10–S17) are available in "HK97-fold proteins evolutionary analyses (sequences, structures, and phylogenies)" (https://doi.org/10.6084/m9.figshare.30948881). Public reference data were obtained from the NCBI Viral Genomes Resource, NCBI RefSeq, and the Protein Data Bank (PDB)."

Other comments:

REVIEWER 2.14

Lines 90-94: The description of the T-number can go into Figure 1 legend, as it disrupts the flow of the narrative here. Also, either define h and k or drop T(h,k. formula.

RESPONSE 2.14

Following the reviewer's recommendations, the explanation of the T-number was moved from the main text to the Figure 1 caption. This includes the description of h and k (the steps on the hexagonal lattice joining neighboring pentamers in the icosahedral architecture).

CHANGES 2.14

Lines 122 to 128, Figure 1 caption: "Size range of HK97-fold compartments showcasing geometric capsid architectures (T-numbers). The T-number (or triangulation number) is the number of major capsid proteins in the asymmetric unit, defined by the equation $T = h^2 + h \cdot k + k^2$, where h and k are two non-negative integers counting the steps needed to join two nearby pentamers along the underlying hexagonal lattice. The h and k determine the sequence of possible icosahedral capsids, T=1, 3, 4, 7, 9, etc. The number of major capsid proteins is 60T, in a closed icosahedral shell (Caspar & Klug, 1962; Twarock & Luque, 2019)."

REVIEWER 2.15

Lines 128-133: why are the mentioned T=3 phages and phage-derived structures not included in Figure 1B among the viral capsid sizes?

RESPONSE 2.15

Figure 1B highlights the absence of HK97-fold viral capsids forming T=1 architectures. An isometric T=3 variant of phage phi29 was included in the revised version. The mentioned gene transfer agent (RcGTA) assembles a shortened, oblate T=3-like head and packages short dsDNA fragments derived from the host genome rather than its own coding sequence, which is 15 kbp in length and does not fit in its oblate T=3 capsid (Bárdy et al., 2020).

CHANGES 2.15

Lines 155 to 159, Introduction section: "The smallest HK97-fold capsid characterized in association [...] oblate T=3-like head (Bárdy et al., 2020)."

Line 118, Figure 1B: Includes the isometric T=3 structure.

REVIEWER 2.16
Lines 134-136: I am not sure why the hypothesis of “encapsulins first” needs to be listed here, as this is not what authors test. If the authors wanted to refer to any earlier work that possibly explored this hypothesis, a citation should be provided.

RESPONSE 2.16
The text was revised to clarify the conceptual tension discussed in the literature: the higher structural complexity of HK97-fold viral capsids relative to encapsulins and the lack of known small HK97-fold viruses. The revised text (i) explicitly attributes this argument to Luque et al. (2020), (ii) contrasts it with the most recent “viruses-to-encapsulin” paradigm (Andreas & Giessen, 2021), and (iii) clarifies that our working hypothesis is that simpler HK97-fold viruses remain under sampled and that identifying them can illuminate ancestral HK97-fold compartments, rather than representing the testing of an “encapsulins-first” scenario.

CHANGES 2.16
Lines 163 to 170, Introduction section: “The greater structural complexity of HK97-fold viral capsids compared with encapsulins [...] Regardless, finding such viruses or confirming their absence would help illuminate ancestral HK97-fold compartments.

REVIEWER 2.17
Lines 154-155: what do the authors refer to when they say “molecularly related”? Aren’t all the analyzed proteins here are homologous and thus “related”?

RESPONSE 2.17
In that context, “molecularly related” referred to the subset of twilight-zone genomes whose HK97-fold proteins display hidden Markov model profiles characteristic of encapsulins. We have removed the term "molecularly related" and explicitly stated the relationship.

CHANGES 2.17
Lines 191 to 192: “Among these small genomes, 93 encoded HK97-fold proteins with encapsulin hidden Markov protein profiles.”

REVIEWER 2.18
Figure 2D: What GTA proteins were included? The two references provided are from PICIs. There are two distinct MCPs in two described types of alphaproteobacterial GTAs (PMID: 36381234).

RESPONSE 2.18
The HK97-fold GTA proteins used in our analyses were those of *Rhodobacter capsulatus* (PDB:6TSU and NCBI: RCAP_RS08360), which belongs to the Alphaproteobacterial type I GTAs according to Kogay et al. 2022. These references were updated accordingly.

CHANGES 2.18
Line 217, Figure 2 panel A: Cites Bárdy et al., 2020 and Strnad et al., 2010 as the sources of GTAs.

REVIEWER 2,19
Figure 2G, “ghost-zone”: did the authors mean to state “twilight zone”?

RESPONSE 2.19

Yes, we fixed the error accordingly.

CHANGES 2.19
Figure S1 now displays the corrected version of Figure 2G.

REVIEWER 2.20
Lines 203-207, definition of the “twilight zone”: On line 203, it is defined as 5-11.6 kb, but then divided into < 5kb (micro. and 5-11.6kb (mini. ones. So, should the “twilight zone” be defined as 0-11.6 kb?

RESPONSE 2.20
In the revised manuscript, the twilight zone was defined as genomes with lengths < 11.6 kbp.

CHANGES 2.20
Lines 180 to 182: “It found 14 candidates encoding HK97-fold proteins in genomes below the isolated threshold of 11.6 kb, which we defined here as the twilight zone of HK97-fold viral genomes.”
Lines 204 to 206: “Twilight zone: metagenomically assembled genomes with lengths < 11.6 kbp; the threshold is defined by the smallest genome from isolated HK97-fold viruses.”
Lines 227 to 228: “The vertical line represents the genome length threshold associated with the smallest isolated *Duplodnaviria* genome, 11.6 kb, and the region below this value (the twilight zone)”

REVIEWER 2.21
Line 239: what is this dataset of 60 genomes? Unclear.

RESPONSE 2.21
These 60 viral genomes are a subset of manually curated representative genomes. This has been clarified in the text.

CHANGES 2.21
Lines 775 to 793: “Finally, HHpred (Söding, 2005) was used for targeted manual curation [...] Manual annotations took precedence over automated assignments when they conflicted. The HHpred annotations are provided in Data File S9.”

REVIEWER 2.22
What statistical test was employed for the results shown in Figure 3C?

RESPONSE 2.22
Encapsulin fraction among positives (>0) was evaluated with a Kruskal–Wallis omnibus test followed by Dunn’s post-hoc pairwise comparisons with Benjamini–Hochberg correction. The revised manuscript specifies the statistical test for Fig. 3C in the Methods section.

CHANGES 2.22
Figure 3C includes significance bars with asterisk annotations for prespecified pairwise contrasts.
Lines 858 to 862: “Because the distribution of encapsulin fraction [...] Pairwise adjusted p-values are reported in Supplementary Table S4.”

Supplementary Table S4 includes the group differences and adjusted p-values.

REVIEWER 2.23
Figure 3D, the solid line: What does a polynomial function summarize across encapsulins and viruses combined? Not described in the figure legend or text.

RESPONSE 2.23
The solid black line represents a nonparametric smoothing spline (generalized additive model) that shows the qualitative trend between the fraction of encapsulin domains and viral genome length. This has been described in the caption as requested.

CHANGES 2.23
Line 281: "The black curve is a generalized additive model (thin-plate regression spline) used for a visual guide."
Lines 862 to 866: "To visualize the relationship between encapsulin domain fraction [...] The smoother was used only as a descriptive aid and was not used for hypothesis testing."

REVIEWER 2.24
In my opinion, the full tree topology (Figure S3. and not the tree snippets (Figure 5C. should be displayed in the main manuscript.

RESPONSE 2.24
Following the reviewer's suggestions, the revised manuscript displays the full structural HK97-fold tree in Figure 5C.

CHANGES 2.24
Line 446, Figure 5C includes the complete structural tree.

REVIEWER 2.25
Line 334-335: GTAs are not taxonomically restricted to Rhodobacter (see PMIDs 28784044 and 36381234).

RESPONSE 2.25
We have updated the manuscript to eliminate the incorrect statement that GTAs are limited to *Rhodobacter*. We thank the reviewer for highlighting this issue.

CHANGES 2.25
Line 903: "1 MCP encoded in a gene transfer agent (GTA)"

REVIEWER 2.26
Line 384: what is the "fringe" zone?

RESPONSE 2.26
The "fringe zone" designates the genome-length range where isolated HK97-fold viruses and the small environmental contigs overlap (11.6–20 kb). It was used as the control region. This has been clarified in the text.

CHANGES 2.26
Lines 206 to 208: "Fringe zone: genomes with lengths between 11.6 kbp and 20 kbp, including both isolated and metagenomically assembled genomes; this region is used as a control."

REVIEWER 2.27
It is confusing that clades are referred using Roman numerals in the text, while in the Figure S3 they are labeled with Arabic numerals.

RESPONSE 2.27
The revised manuscript uses only Arabic numerals to refer to the clades (1-9), consistent across the text and figures.

CHANGES 2.27
Lines 407, 416, 419, 422, 425, 427, 430, and Figure 5

REVIEWER 2.28
Conclusions, line 643: which of the discussed clades do the authors refer to here?

RESPONSE 2.28
The clades referred to are those including viral HK97-fold MCPs displaying encapsulin profiles as a particular group of interest for follow-up work. This has been clarified in the text.

CHANGES 2.28
Lines 708 to 711: "In particular, the HK97-fold major capsid proteins displaying encapsulin-like domains in the twilight zone genome range provide a tractable set of candidates for follow-up theoretical and experimental work to probe their architecture, potential novel packaging strategies, and evolution of HK97-fold compartments."

Reviewer #2 (Remarks on code availability):

REVIEWER 2.29
This is the first time I see the link to the code it should be listed in the manuscript.

RESPONSE 2.29
The revised manuscript includes links to the GitHub repository in the Methods and Data Availability sections.

CHANGES 2.29
Line 718, Methods section: "https://github.com/luquelab/virus_encapsulin_2025."
Line 942, Data Availability section: "https://github.com/luquelab/virus_encapsulin_2025."

Reviewer #3 (Remarks to the Author):

I co-reviewed this manuscript with one of the reviewers who provided the listed reports. This is part of the Nature Communications initiative to facilitate training in peer review and to provide appropriate recognition for Early Career Researchers who co-review manuscripts.

Reviewer #3 (Remarks on code availability):

REVIEWER ECR 2.0
I reviewed the python code in the GitHub repository. It works quite ok and easily runnable from CLI. Some details about what each particular script does is lacking, but I for reproducibility purposes I found all information to be sufficient.

RESPONSE ECR 2.0
We thank the reviewer for checking and testing the code. Following the reviewer's suggestion, the repository now includes brief descriptions of the main scripts describing their purpose and usage.

CHANGES ECR 2.0
GitHub repository: Revised README files and in-script comments.

Reviewer #4 (Remarks to the Author):

I co-reviewed this manuscript with one of the reviewers who provided the listed reports. This is part of the Nature Communications initiative to facilitate training in peer review and to provide appropriate recognition for Early Career Researchers who co-review manuscripts.

RESPONSE 4.0
We thank the reviewer for their assistance during the review process.

RESPONSE TO REVIEWERS

Reviewer #1 (Remarks to the Author)

REVIEWER 1.0

This review is in response to the revised version of the manuscript. Thanks to the authors for their revisions, I think that it improved the manuscript. By its nature, this work is somewhat speculative, but I think that the authors have made a strong argument and have presented some intriguing models for future research.

RESPONSE 1.0

We thank the reviewer for the positive assessment of the revised manuscript and for noting that the revisions improved the work. We addressed the remaining comments below and revised the manuscript's text and figures accordingly. The line numbers in the response refer to the marked-up manuscript, which highlights added text in red and includes deleted text in blue with strikethrough. This applies to all changes in the rebuttal.

Major comments

REVIEWER 1.1

My only major comment is that the authors state in line 435 of the marked up manuscript "the tree topology differed slightly from that in the structure-based analysis" in regard to Figure S6. I disagree that the topology of the two trees is only slightly different, it appears quite different to me. Perhaps "somewhat" instead of "slightly" or omit "slightly" or reword this sentence.

RESPONSE 1.1

We agree that the original wording understated the topological differences between the two trees. To improve the comparison between the trees' topologies, we recalculated the structure-based tree using maximum-likelihood inference, as for the sequence-based tree. This provided a more direct comparison between the trees. It also enabled calculation of support values, clarifying uncertainties in deep branches, as requested by Reviewer 2. The revised structure-based tree is more similar to the sequence-based tree than the previous structure-based tree. It resolved several discrepancies, including the clustering of encapsulin Family IV, which is now grouped together in both trees (see Figure S6). Both analyses recover the three main mixed virus-encapsulin groups (Structural Groups 1, 4, 5) and the virus group containing encapsulin and viral domains (Structural Group 6) (see Figure S4), again indicating the robustness of our evolutionary finding. The remaining differences mainly involve deeper relationships among groups, likely reflecting the different information captured by structure- and sequence-based inference and the limited resolution of the deep branches (Figure S6). The revised text also states that the sequence-based tree does not recover three of the nine structure-based groups (Structural Groups 7, 8, and 9), which were formed exclusively by viruses. In the sentence highlighted by the reviewer, we now make this contrast explicit by stating that the two trees agree in the recovery of the main mixed virus-encapsulin groups, despite differences in their overall topology.

+ Lines 419 to 421 (Results): “Despite differing from the structure-based tree in overall topology, the sequence-based tree also recovered three mixed virus-encapsulin groups (Figure S5).”

+ Lines 427 to 430 (Results): “Conversely, the viral-exclusive Structural Groups 7,8, and 9 lacked counterparts in the sequence tree, likely reflecting the limited resolution of deeper relationships and the different information captured by structure- and sequence-based inference (Figure S6).”

REVIEWER 1.2

Throughout the manuscript be careful about use of italics. Virus names themselves should not be italicized according to the ICTV (https://ictv.global/faq/names), only formal taxonomic ranks (e.g. family, genus, species) should be italicized.

RESPONSE 1.2

We revised the use of italics to align with the ICTV nomenclature, as the reviewer noted. Italics were standardized throughout the manuscript: The formal ICTV taxonomic ranks *Duplodnaviria* and *Microviridae* remain italicized; the host-containing phrases *Staphylococcus aureus* pathogenic island 1, *Rhodobacter capsulatus* gene transfer agent, and *Myxococcus xanthus* cargo are now only partially italicized where appropriate; and virus names and informal virus group names are presented in roman type.

REVIEWER 1.3

L123: "Dashed red box" appears to be missing from figure in the integrated PDF.

RESPONSE 1.3

We revised the caption for Figure 1 to match the elements displayed. The reference to the dashed red box was removed. See line 113 (Figure 1 - caption).

REVIEWER 1.4

L166: There is an additional period after "viruses."

RESPONSE 1.4

We corrected this typographical error. See line 153 (Introduction).

REVIEWER 1.5

L367: Please check to make sure that analyzed is not removed in the revised text (it is both underlined and struck through in the marked-up manuscript).

RESPONSE 1.5

We corrected the track changes artifact. The intended wording is now preserved in the revised manuscript in lines 330 to 331.

REVIEWER 1.6

L518: The Helix-Turn-Helix motif (HTH) generally refers to a DNA-binding motif. These structures do not appear to be related to that motif, so consider using different terminology, 2 long alpha helices, for instance.

RESPONSE 1.6

We thank the reviewer for highlighting the nuance regarding the "helix-turn-helix." It was not our intention to suggest that the motif has a DNA-binding function. In the revised manuscript, we used the more general "alpha-helical hairpin" terminology to describe the

motif's structural topology (Du & Gai, 2006). See lines 488 (Results), 470 (Figure 6 caption), 526 (Discussion).

Reviewer #2 (Remarks to the Author)

REVIEWER 2.0

The revised manuscript has improved substantially. I have several follow-up comments that arose when the authors addressed some of my earlier concerns.

RESPONSE 2.0

We thank the reviewer for carefully re-evaluating the revised manuscript and for noting that it has improved substantially. The reviewer's follow-up comments prompted us to further revise the analysis of the structure-based tree. This revision strengthened the consistency of the manuscript's main results, as detailed below. The cited lines in the changes below refer to the marked-up manuscript, which highlights added text in red and shows deleted text in blue with strikethrough. This applies to all changes in the rebuttal.

Major comments

REVIEWER 2.1

1) Mid-point rooting, while convenient, does not necessarily root a tree at the most ancestral node, as the technique is very sensitive to differences in rates of evolution on different branches (something one expects in dataset that contains cellular and viral proteins). Unfortunately, that also means that the mid-point rooted tree is essentially should be treated as unrooted and thus does not allow to make inferences about ancestor-descendant relationships. This is important for two inferences in the manuscript: a. To define “clades” one needs to know ancestor-descendant relationships on a tree. One solution would be to avoid term “clade” and use word “group”. b. Figures 6B and 6D (the model) use mid-point rooted tree to make inferences about which group was ancestral.

RESPONSE 2.1

The revised text uses the term "group" instead of "clade", following the reviewer's suggestion that the lack of an optimal rooting strategy precludes inferences about ancestor-descendant relationships and the identification of clades. We also unrooted the schematic evolutionary model in Figure 6B to account for uncertainty at deeper branches, avoding ancestor-descendant claims. Additionally, we clarified that Figure 6D presents a biophysical evolutionary model that complements the consensus phylogenetic analysis of Figure 6B, using biophysical arguments grounded in observations to offer a plausible scenario and specific hypotheses for the early evolution of the HK97-fold protein. Figure 6D was revised to clarify key steps in the biophysical evolutionary model.

+ The term "group" was used instead of "clade" across the manuscript.
+ Figure 6B has been revised to reflect an unrooted tree.
+ Figure 6D has been revised to clarify the biophysical elements in the evolutionary model.
+ Lines 609 to 614 (Discussion): “The lack of an optimal rooting strategy, together with the low support values for the deep branches, prevents reliable inference of a virus-like or encapsulin-like ancestor and precludes strong directionality claims based on the phylogenetic analyses. However, we speculate a biophysically grounded evolutionary model of increasing complexity to complement the unresolved relationships from the phylogenetic analyses (Figure 6D).”

REVIEWER 2.2

2) Aside from mid-point rooting, the phylogenetic tree for which bootstrap support values are shown (Figure S4) demonstrates very poor resolution for most “clades” (and for the deepest branches on the tree), which is OK for the overall conclusions of the manuscript (the intertwined evolution of encapsulins and viruses), but not good for scenario shown on Figure 6B. Also, the structure-based tree (no statistical support provided) essentially has deep-branches of length 0, and thus suggests that there is no resolution among various groups on this tree, again making directional inferences on figures 6B and 6D speculative.

RESPONSE 2.2

To address low branch support in deep branches, we revised the structural tree calculation by using an alternative FoldTree workflow that converts structures into 3Di representations and infers the tree within a maximum-likelihood framework, enabling evaluation of bootstrap branch support values (Figures 5, S4, S6). The revised structural tree shows low support across deep branches, as in the sequence tree (Figure S6). Nonetheless, three mixed virus-encapsulin groups are still recovered, with support values ranging from 61 to 94. The schematic tree in Figure 6B was revised and simplified into a minimal consensus model based on inferences from the structural tree, the phylogenetic tree, and shared sequence domains across mixed groups. As mentioned in the prior response, this revised schematic tree was unrooted to avoid making directional claims and to highlight the lack of resolution at deep branches. The revised text clarifies that the biophysically grounded model (Figure 6D) aims to complement the unresolved ancestral relationships in the phylogenetic analyses. Figure 6D was revised to clarify key steps in the biophysical evolutionary model.

+ Lines 419 to 421 (Results): “Despite differing from the structure-based tree in overall topology, the sequence-based tree also recovered three mixed virus-encapsulin groups (Figure S5).”

+ Lines 427 to 430 (Results): “Conversely, the viral-exclusive Structural Groups 7, 8, and 9 lacked counterparts in the sequence tree, likely reflecting the limited resolution of deeper relationships and the different information captured by structure- and sequence-based inference (Figure S6).”

+ Revised Figures (structure-based tree updates): 5C, S4, and S6.

+ Lines 856 to 862 (Methods): "Foldseek-derived 3Di encodings (van Kempen et al., 2024) to represent local tertiary geometry as residue-level structural states, thereby avoiding issues associated with global structural metrics. The resulting 3Di alignment was analyzed in IQ-TREE under a maximum-likelihood framework using empirical 3Di-state frequencies and allowing a fraction of invariant sites (F+I), with 3000 bootstrap replicates."

REVIEWER 2.3

3) Comment on “RESPONSE 2.8 Trees in the revised manuscript display branch lengths proportional to the underlying distance metric: substitutions per site for sequence-based trees and structural distances for structure-based trees. For the sequence-based tree, bootstrap support values were included.” a. The scale units for the trees should be listed in the legends for Figures S6 and 5. Scales must have units! For the structure trees, what are the units? Did the authors use 3Di encodings to make the trees, or some other structural distance metric? b. Statistical support values should be shown on all trees (Fig 4, Fig 5, Fig S6), including for structure-based tree. Based on Figure S6, the structural tree is so different from the amino-acid sequence tree, but I suspect this is because structure-based tree has very low resolution on group-defining branches.

RESPONSE 2.3
The revised manuscript includes the tree scales and bootstrap support values for all trees. For the sequence-based tree, branch lengths are reported as expected substitutions per aligned site. The structure-based tree was revised using the FoldTree maximum-likelihood workflow, which uses Foldseek-derived 3Di structural encodings, replacing the previous pairwise-distance and neighbor-joining workflow, which did not estimate support values. The branch lengths in the revised structure-based tree are in units of expected substitutions per aligned 3Di site. The aligned sites are discrete residue-level structural states that encode local tertiary geometry. The support values for the revised structural tree were estimated in IQ-TREE from 3,000 bootstrap iterations, as we did previously with the phylogenetic tree. The revised structure-based tree resolved some of the prior discrepancies with the sequence-based tree, such as the grouping of encapsulin Family IV. The revised structural tree recovered the virus-encapsulin mixed groups of interest, as did the sequence-based tree, demonstrating the robustness of the results.
+ Lines 856 to 862 (Methods): Text shared in the prior response (Response 2.2).
+ Revised legends now explicitly indicate the units of the tree scales: 5C, S4, S5, and S6
+ Lines 360 to 360 (Figure 5C - caption): "Branch lengths are reported as expected substitutions per aligned 3Di site, and node labels indicate bootstrap support values." The caption of Figure S5 was revised accordingly.
+ Revised Figures now reporting support values: 4A, 5C, S4, S5, and S6.

Minor comments

REVIEWER 2.4
p. 12, line 238: typo - remove "while".

RESPONSE 2.4
We removed the typographical error. See line 241.

REVIEWER 2.5
p.20, line 412: "unrelated" should be replaced with "divergent" or "poorly conserved", as unrelated means no common ancestor and that contradicts the main argument of the paper (shared ancestry of encapsulins and viral capsids).

RESPONSE 2.5
We revised this sentence. It now uses "poorly conserved," as recommended by the reviewer. See line 454.

REVIEWER 2.6
Figure 2C: Predicted T-numbers. Should any values between $1<T<3$ be assigned to $T=3$, as there are now capsids with fractional T number?

RESPONSE 2.6
We have revised Figure 2C and the text to clarify this observation. The manuscript relies on the generalized icosahedral framework of Twarock and Luque (Nature Communications, 2019). In that framework, non-integer values are possible for icosahedral lattices that accommodate minor proteins, which were not described in the classical Caspar-Klug T-number series. Some of these alternative lattices, such as the

trihexagonal lattice, have been observed in HK97-fold viruses that encode minor proteins (Podgorski et al., 2025). We also indicate that these intermediate T-numbers could be interpreted as elongated "T=1" or shrunken "T=3" shells, consistent with the theory of elongated capsids (Luque and Reguera, Biophysical J. 2010; Luque et al., PNAS 2010) and with the observation of gene transfer agent (GTA) HK97-fold shells (Bardy et al., 2021). The text in the Methods section and Figure 2 was revised accordingly.

+ Lines 208 to 211 (Figure 2, caption): “Predicted T-numbers include both canonical icosahedral architectures and non-canonical icosahedral lattices from the generalized icosahedral framework (Twarock & Luque 2019), such as the trihexagonal (T≈1.33), snub hexagonal (T≈2.33), and rhombitrihexagonal (T≈2.49) lattices, which incorporate minor capsid proteins.”

+ Lines 695 to 700 (Methods): "Predictions were interpreted within the generalized icosahedral framework of Twarock and Luque (2019), which includes both canonical T=1 icosahedral capsids and non-canonical architectures associated with alternative lattices that accommodate minor proteins, such as the trihexagonal (T=4/3), snub hexagonal (T=7/3), and rhombitrihexagonal (T=5/2) lattices."

+ Lines 702 to 704 (Methods): "as elongated T=1 capsids (Luque et al. 2010; Luque & Reguera, 2010) or shrunken T=3 capsids, consistent with a reported gene transfer agent (GTA) HK97-fold capsid (Bárdy et al., 2020)."
